# The use of adenoviral vectors in gene therapy and vaccine approaches

**DOI:** 10.1590/1678-4685-GMB-2022-0079

**Published:** 2022-10-07

**Authors:** Natália Meneses Araújo, Ileana Gabriela Sanchez Rubio, Nicholas Pietro Agulha Toneto, Mirian Galliote Morale, Rodrigo Esaki Tamura

**Affiliations:** 1 Universidade Federal de São Paulo, Laboratório de Biologia Molecular do Câncer, São Paulo, SP, Brazil.; 2 Universidade Federal de São Paulo, Departamento de Ciências Biológicas, Diadema, SP, Brazil.; 3 Universidade Federal de São Paulo, Laboratório de Ciências Moleculares da Tireóide, Diadema, SP, Brazil.

**Keywords:** Adenovirus, gene therapy, monogenic diseases, cancer, vaccines

## Abstract

Adenovirus was first identified in the 1950s and since then this pathogenic group of viruses has been explored and transformed into a genetic transfer vehicle. Modification or deletion of few genes are necessary to transform it into a conditionally or non-replicative vector, creating a versatile tool capable of transducing different tissues and inducing high levels of transgene expression. In the early years of vector development, the application in monogenic diseases faced several hurdles, including short-term gene expression and even a fatality. On the other hand, an adenoviral delivery strategy for treatment of cancer was the first approved gene therapy product. There is an increasing interest in expressing transgenes with therapeutic potential targeting the cancer hallmarks, inhibiting metastasis, inducing cancer cell death or modulating the immune system to attack the tumor cells. Replicative adenovirus as vaccines may be even older and date to a few years of its discovery, application of non-replicative adenovirus for vaccination against different microorganisms has been investigated, but only recently, it demonstrated its full potential being one of the leading vaccination tools for COVID-19. This is not a new vector nor a new technology, but the result of decades of careful and intense work in this field.

## Introduction

Adenoviruses were first identified in 1953 after an analysis of tissue culture of tonsils and adenoids, that was aiming to identify unknown viruses from the respiratory tract that could cause acute respiratory diseases. Huebner *et al.* identified 13 new agents in surgically removed adenoids. Because the main symptoms presented by patients were acute pharyngitis and conjunctivitis, the authors proposed the term “adenoidal-pharyngeal-conjunctival agents” to designate this group of viruses, but posteriorly the name has changed to adenovirus, referring to the tissue of its first reported isolation (Robbins et al., 1950; Huebner *et al.,* 1954).

**Figure 1 -:**
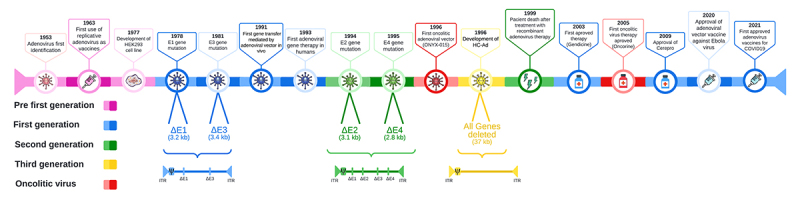
Timeline of adenoviral vectors generations. The highlights researches of adenovirus gene therapy development, from pre-first-generation experiments until the third generation including the first approved drugs for cancer treatment, oncolitic adenoviral vectos and adenoviral vaccines. HC-Ad: High-capacity adenovirus; delta: deleted.

Data provided from the Journal of Gene Medicine indicates that adenoviral vectors are the most used vector type for gene transfer, representing 17.5% of all gene therapy clinical trials (Gene Therapy Clinical Trials Worldwide - GTCT, 2021). They are most commonly employed in cancer therapies, but can also be applied in vaccinal approaches and treatment of monogenic diseases. Its extensive applications are due to intrinsic adenoviral vector characteristics: non-integration in the host genome and high capacity for gene transfer and storage. Although adenoviruses are pathogenic and associated with respiratory and gastrointestinal diseases, modifications of their genome have been made to turn the adenoviral vectors safe and to avoid adverse effects of the therapy. These genetic modifications on the viral genome generated a replication-defective vector, preventing a high viral load in the host body. The evolution of adenoviral vectors development is shown in Figure 1.

## First generation adenoviral vectors

In 1977, a cell line that is necessary for recombinant non-replicative adenoviral vectors was raised. The human embryonic kidney (HEK) cells were modified with human adenovirus type 5 (Ad5) DNA fragments and the particular clone 293 (HEK293) was transformed by the acquisition of 4 copies of the left end of Ad5 genome, a region that includes the E1 gene. Thus, HEK293 was the first established human cell transformed by an adenovirus (Graham et al., 1977), which made possible the development of the first generation of recombinant adenoviruses presenting deletions in the E1 and E3 genes, that are associated with the expression of all other genes involved in viral replication and inhibition of host immune system, respectively. 

The E1 region is divided into two parts: E1a and E1b. A group of mutants with deletions on region E1 was isolated and infected HEK293 cells *in vitro* to observe if adenoviruses were able of growing on it (Jones and Shenk, 1978, 1979a,b). The authors identified two mutants, one lacking E1a (deletion of 902 bp, around position 540-1620 bp of the genome) and other E1b (deletion of 2350 pb, around position 1260-3780 bp of the genome) that were able to replicate in HEK293 cell lines, but neither in HeLa nor HEK cells. Concluding that E1 gene was necessary for viral growth, which was only possible in HEK293 cells, that contains the E1 gene supporting viral replication of the mutant adenoviruses. Later, deletions with a maximum of 3 kb have been made in this region to generate E1 deleted adenovirus vectors with a capacity of insertion up to 5 kb (Rosenfeld et al., 1991). 

The E3 gene has its expression activated by E1a gene product and encodes proteins that counteract the attack of the immune system and prevents programmed cell death. Thus, the E3 gene products are not related to viral replication, and therefore no complementing cell line is necessary (Wold et al., 1995). Viral vector with deletion of the E3 gene (from 28kb to 30kb) was indistinguishable from wild type (WT) adenovirus in growth kinetics (Cladaras et al., 1985). Vectors deleted in the E1 and E3 genes have a storage capacity of approximately 8 kb.

## Second-generation adenoviral vectors

Although adenoviral vectors are successful in gene transferring and expression, some concerns were raised. *In vivo* delivery of recombinant adenoviral vector carrying the LacZ gene in the liver showed low levels of transgene expression and induction of cellular immune response, leading to destruction of genetically modified hepatocytes and repopulation with parental cells, without the transgene (Yang et al., 1994). This probably occurred due to the background expression and accumulation of viral late genes, leading to inflammation and destruction of transduced cells (Gilgenkrantz et al., 1995; Yang *et al.,* 1996). In view of that, new recombinant adenovirus needed to be developed, with new mutations on other viral genes. In this context, second generation adenoviral vectors were developed, including additional mutations or deletions of E2 and E4 genes. Both genes participate in the expression of late genes, and their absence reduced adverse effects caused by the expression of the late genes. Furthermore, there was an increased storage capacity, allowing it to accommodate up to 14 kb.

In 1994, the first modification of first-generation adenovirus was performed. Alongside E1 deletion, it incorporated a mutation into the E2a region, turning this gene temperature-sensitive (Engelhardt et al., 1994). E2a gene encodes a single-stranded DNA binding protein that is responsible for DNA synthesis. Recombinant adenovirus with E2a temperature-sensitive mutation has reduced late protein expression levels. In contrast, the E4 gene encodes two ORFs, ORF3 and ORF6, which participate in viral DNA synthesis and expression of late genes (Sandler and Ketner, 1989). ORF 6 gene product forms a complex with E1b and mediates the transport of viral messenger RNA from the nucleus to the cytoplasm and ORF 3 gene product acts in parallel with this complex to enable viral DNA replication. Deletion of the E4 gene blocks adenoviral replication and is lethal (Halbert et al., 1985). Differently of E2 temperature-sensitive mutation, deletion of the E4 gene entails the need of a cell line capable of complementing its absence, but without overexpressing cytotoxic late proteins. In 1995, a HEK293 cell line expressing E4 was established by introducing a full-length E4 region under control of the mouse alpha inhibin promoter, enabling the production of E1/E4-deleted adenovirus vectors (Wang et al., 1995). However, even with these new gene deletions, second-generation vectors still do not avoid completely *in vivo* immunogenicity (Lusky et al., 1998). 

Different barriers have been considered for safe and effective adenoviral-mediated gene therapy such as: (1) the severe innate and adaptative immune responses against vectors and transgenes that lead to severe adverse side effects (Tripathy et al., 1996; Harvey et al., 2002); (2) the high pre-existing immunity against adenovirus in the population (Barouch et al., 2011; Ye et al., 2018) that can hamper the efficacy of the treatment due to neutralizing antibodies that rapidly blocks the virus (Tripathy *et al.,* 1996; Kushwah et al., 2008; Parker et al., 2009); (3) the elimination of Ad vectors through liver and spleen after intravenous applications due to interactions between Ad vector and host proteins (Parker *et al.,* 2006); (4) the natural tropism of most adenovirus through the attachment of the Ad fiber knob protein with CAR, which is expressed in a huge range of tissues making it difficult to transduce only specific cells (Bewley et al., 1999; Einfeld et al., 2001). Therefore, additional alterations in the adenoviral vectors have been developed.

## Third-generation adenoviral vectors

To resolve some of these questions, the third and last generation of adenoviral vectors were created, also called gutless, helper-dependent (HD-Ad), or high-capacity (HC-Ad) vectors. This vector has all the viral genes deleted, keeping only the ITRs and the packaging sequence. Because of this modification, the HC-Ad vector needs a helper adenoviral vector encoding all viral genes. When both vectors are coinfected in a eukaryotic cell line, the helper adenovirus produces the structural proteins, which will be assembled into the capsid particle incorporating the HC-Ad genome. Due to the deletion of all viral genes, the helper-dependent adenovirus has a capacity of gene insertion up to 37 kb. The biggest limitation for its broader use is the incorporation of the helper virus genome into the capsid. Therefore, the final product is a mixture of HC-Ad and contaminating helper virus (Alba et al., 2005). The first strategy that tried to overcome this problem was developed by Mitani and colleaagues who used an Ad5 with a defective packaging signal as the helper virus, while the gutless vector had deletions of only L1, L2, VA, and TP genes. However, during viral vector production, both HC-Ad and helper virus were obtained (Mitani *et al.,* 1995). 

An important advance was the development of Ad helper virus containing the packaging signals flanked by*lox*P sites, which were excised by the Cre recombinase, rendering the helper virus genome unpackageable and producing high titers of the vector with very low quantities of contaminating helper virus, which was still present at a range around 0,1% - 10% (Parks et al., 1996). These high levels of contamination were due to the enzyme activity, that cannot remove 100% of packaging signals in the helper virus. Indeed, this system provides increased cloning capacity, safety, and reduced immunogenicity, but contamination by helper virus is still a problem. 

Since the development of this system, many similar techniques have been developed and they suffer from the same problems: the difficulty of vector production and the presence of helper virus contaminations. Another improvement in Cre/loxP system was based on the reversion of the packaging sequence of helper adenovirus. This system provided lower levels of helper contaminations, around 0,02 - 0,1%, and improved vector production (Palmer and Ng, 2003). Other recombinase systems were also explored, such as the FLP/frt system (Ng *et al.,* 2001) and the Vika recombinase system (Phillips et al., 2022). However, none of these approaches completely eliminates the presence of the helper virus.

The Helper-virus-free strategy involves the co-transfection of the HC-Ad with a helper plasmid. Using this approach, vectors expressing the human dystrophin and huntingtin genes were produced on large scale and efficiently delivered into cells and mouse models, showing therapeutic potential for Huntington’s disease and Duchenne muscular dystrophy (Lee et al., 2019). 

Comparing replicative, first-generation, and HC-Ad for vaccination purposes, it was observed that replicative and HC-Ad induced stronger humoral immune response, but not cellular immune response, while HC-Ad also induced lower ALT levels compared with replicative and first-generation adenoviral vectors, indicating a possible reduced liver toxicity (Weaver et al., 2009).

## Conditionally replicative adenoviral vectors

Besides all attempts and modifications involving replicative-defective adenovirus, a different approach maintains its replication capacity. Conditionally replicating adenoviruses have been employed as oncolytic adenoviruses, showing replicative potential only in tumor cells, destroying them in the process and continuously disseminating and replicating in cancer cells. One of the first examples is the Onyx-015, which has an alteration in the E1B-55K gene (Bischoff et al., 1996). Lack of E1B-55K inhibited late viral RNA export from the nucleus to the cytoplasm preventing expression of late genes in normal cells. However, in tumor cells, the viral RNA is exported independently of the presence of E1B-55K and viral proteins expression and replication occurs (O’Shea et al., 2004). Oncorine (Creative Biolabs, Inc., Shirley, NY) is similar to ONYX-015 and was the first oncolytic adenovirus approved for the treatment of nasopharyngeal carcinoma in China (Liang, 2018). Further examples are seen in Adenovirus in cancer gene therapy section.

## Investigation of other adenovirus types and modifications

All early adenoviral studies were conducted in type 2 and 5 human adenoviruses, therefore gathered knowledge is deeper in these types compared to other adenoviruses. However, the presence of neutralizing antibodies (Dudareva et al., 2009; Pilankatta et al., 2010; Barouch et al., 2011; Zhang et al., 2013b; Su et al., 2016; Zhao et al., 2018) may impair gene transfer mediated by them. Cotton rats previously infected with WT Ad5 had reduced immunization efficacy mediated by an Ad5 non-replicative vector (Papp et al., 1999a).

In order to overcome this problem, other adenoviruses have been evaluated. Ad35 has a low global prevalence and has been further studied (Gao et al., 2003; Vogels et al., 2003; Nwanegbo et al., 2004). It has a tropism to cells with CD46 receptor rather than cells expressing CAR (coxsackie and adenovirus receptor), but this can be overcome by construction of a chimeric Ad35 expressing the Ad5 fiber knob (Nanda et al., 2005). Several other adenoviruses with low seroprevalence have been engineered into non-replicative adenoviral vectors, such as: Ad11 (Holterman et al., 2004); Ad41 (Lemiale et al., 2007); Ad56 (Duffy et al., 2018); Ad19a, which transduces dendritic cells (Ragonnaud et al., 2018); Ad20-42-42, which is related to type 42 but with a penton base derived from type 20 and tropism to both CAR and CD46 receptors (Ballmann et al., 2021); Ad26, Ad48 and Ad50 are rare types, the Ad26 vector was shown to be the most immunogenic and more interesting in vaccine development (Abbink et al., 2007). Ad26 uses sialic acid as a primary target in the cell (Baker et al., 2019).

The Ad5 can be altered to reduce the binding of neutralizing antibodies. The adenoviral hexon protein is a major component that drives the host immune response. Replacing the hexon of Ad5 with the one from Ad3 reduced neutralization of viral particles (Yan et al., 2021). As well exchange of the hexon gene of Ad3 with the hexon from Ad14 generated a chimeric vector that was not neutralized by antibodies against Ad3 (Su et al., 2016). Modifications of hypervariable regions within the hexon gene could also impair antibodies against Ad5 binding. A chimeric hexon protein from Ad5, with replacement of some regions from Ad48, circumvented pre-existing immunogenicity (Roberts *et al.,* 2006; Teigler et al., 2014). Modification of a hypervariable region 2 of Ad5 with the region from Ad3 also reduced neutralization (Gu et al., 2016). Alteration of all hypervariable regions from Ad5 introducing the regions from Ad43 had the same effect (Bruder et al., 2012). Epitope modification in the 5^th^ hypervariable region of Ad5 also prevented antibody neutralization (Abe et al., 2009). Modification of both hexon and fiber proteins abrogated adenoviral vector neutralization (Bradley et al., 2012), and chimeric Ad5 with fiber from Ad35 escaped neutralization (Flickinger et al., 2020).

Additionally, several adenoviruses infecting other mammals and capable of infecting human cells have been investigated, including adenovirus from bovine type 3 (Mittal et al., 1995), chimpanzee (ChAd) type 68 (Xiang et al., 2002), types 5, 6, 7 (Roy et al., 2004), C1 (Tatsis et al., 2007a) and Y25 (Dicks et al., 2012), rhesus monkey types 51, 52 and 53 (Abbink et al., 2015), porcine type 3 (Bangari and Mittal, 2004) and simian type 21 (Roy *et al.,* 2006). Clinical trial data indicated that ChAd63 is safe and induces a strong immune response (O’Hara et al., 2012). A vector derived from ChAdY25 was obtained by removal of E1 and E3 genes and the E4 gene was modified to optimize growth rate in human cell lines, generating the ChAdOx1(Dicks *et al.,* 2012), making the same alterations in ChAd68 it was generated the ChAdOx2 (Morris et al., 2016). 

Even tough neutralization assays are important tools to evaluate inhibition of viral vector transduction efficiency, it was observed that a ChAd68 adenoviral vector modified in the hexon protein resisted neutralization by antisera of animals immunized with WT ChAd68, but failed to transduce target cells and express the transgene, suggesting that neutralization assay may not be a reliable test to predict vector transduction efficiency (Pichla-Gollon et al., 2009). Induction of antibody response against transgene expression mediated by Ad26 and ChAd6 and ChAd7 were lower in comparison with Ad5, suggesting that Ad5 is more efficient to induce high levels of gene expression and immune response (Chen et al., 2010). Another interesting data is that use of prime-boost regimens with combination of different adenoviral types did not improve immune response (Weaver et al., 2009). However, monkeys immunized with a combination of Ad26 and Ad5 expressing Gag protein of Simian Immunodeficiency Virus (SIV-Gag) showed increased cellular immune response and survival after SIV challenge (Liu et al., 2009). Ad5 vectors elicited higher memory T cell activation magnitude, but can also cause functional exhaustion and reduced potency after boost compared to Ad26, Ad35, and Ad48 vectors (Penaloza-MacMaster et al., 2013). This topic will be further discussed in the adenovirus modulation of the immune system session.

Components of the viral particle have also been modified. The introduction of the tripeptide arg-gly-asp (RGD) conferred altered tropism for the viral particle, making it capable of transducing dendritic cells (Worgall et al., 2004) and other cells expressing integrins. Replacement of the fiber protein with Sigma 1 from reovirus changed viral tropism to junctional adhesion molecule 1 (JAM1) and sialic acid (Weaver et al., 2012). The viral particle can also be covered with different compounds to avoid immunologic destruction, using for example alginate microspheres (Sailaja et al., 2002), or coating with non-immunogenic polymers, such as polyethylene glycol, which reduces vector immunogenicity and protect the virus against neutralizing antibody for persistent gene expression (Prill et al., 2011; Sun et al., 2019b). At the same time, adjuvant formulations may increase immunological response in vaccination protocols, and formulations including chitosan and glycol chitosan improve intranasal immunogenicity of Ad5 vector (Gogev et al., 2004). 

## Adenovirus gene therapy for monogenic diseases

### The early years

Since the seminal idea of Friedmann and Roblin (1972) proposing gene therapy to ameliorate human genetic diseases, several experiments have been conducted *in situ*, *in vivo*, and *ex-vivo* to introduce a functional gene or to modulate its expression in a target cell. The use of viral and non-viral vectors for gene delivery and gene editing for permanent correction of patient gene defects are being explored for decades and the promises are starting to become reality (Bulcha et al., 2021). 

Initially, recombinant adenoviral vectors were employed in therapies for common hereditary respiratory diseases, due to their capacity of infecting lung epithelium. The first *in vivo* therapy used a replication-deficient first-generation adenoviral vector to deliver the alfa-1 antitrypsin gene firstly in lung tissues and then in rat hepatocytes, showing that adenovirus can be used as a vector to treat diseases affecting other sites beyond the lung. The rationale was to convert homozygous mutated hepatocytes cells into heterozygotes, which would not manifest the disease phenotype (Crystal, 1990; Rosenfeld et al., 1991; Jaffe et al., 1992). Next, recombinant adenovirus (Ad/CFTR) was employed for gene therapy for cystic fibrosis (CF) through the delivery of the cystic fibrosis transmembrane conductance regulator (*CFTR*) cDNA. Studies in human bronchial cells (Rich et al., 1993), human bronchial xenograft model (Engelhardt et al., 1993b), and nonhuman primates (Engelhardt *et al.,* 1993a; Goldman et al., 1995) showed the feasibility and safety of this technology. Even though in the early 1990s there was limited knowledge regarding the safety and effectiveness of gene delivery by first-generation adenovirus vectors in humans, in 1993 it was performed the first clinical trial for human gene therapy with a recombinant adenovirus (AD2/CFTR) in three individulas. The treatment partially corrected the chloride transport defect characteristic of the CF without evidence of adverse effects (Zabner et al., 1993). In another study, Ad/CFTR was administrated to the nasal and bronchial epithelium of the CF patients. At a high dose, transient systemic inflammation was observed after administration without long-term adverse effects (Crystal *et al.,* 1994). At the same time, other clinical trials were initiated and showed similar results (Zabner *et al.,* 1993, 1996; Crystal *et al.,* 1994; Zuckerman et al., 1999).

These approaches of gene therapy for genetic diseases seemed promising until 1999 when a patient died after treatment with a second-generation Ad5 vector carrying the human ornithine transcarboxylase (*OTC*) cDNA for OTC deficiency (Raper et al., 2003). The 18 years old patient was the only one among other 17 OTC deficient patients who died 96 h after gene transfer due to a systemic inflammatory response syndrome. The other patients experienced only flu-like symptoms. A recent study showed that the presence of a complex of pre-existing Ad5 antibodies and the Ad-therapeutic vector could enhance vector transduction and activation of dendritic cells, which may have contributed to the systemic lethal inflammation of that patient (Somanathan et al., 2020). This adverse result rocked the gene therapy research and delayed advances for some time. 

The use of HC-Ad was able to overcome some of the limitations of first and second-generation vectors and was employed in some strategies. In nonhuman primate models, the expression of the baboon alpha-fetoprotein transgene delivered by a HC-Ad persisted up to 7 years without adverse effects, declining to about 10%/year (Brunetti-Pierri et al., 2013). In a mouse model of primary kidney disease hyperoxaluria type 1, HC-Ad transferred the alanine-glyoxylate aminotransferase gene under control of a liver-specific promoter, improving the clinical condition of the animals for at least 24 weeks (Castello et al., 2016). In another study, primary dystrophin-deficient mouse myoblasts were successfully transduced with an adenoviral vector carrying the full-length murine dystrophin cDNA under control of a muscle-specific promoter and a lacZ reporter construct (Kochanek et al., 1996). 

### Pre-clinical and clinical trials

Next, we present pre-clinical and clinical results of some monogenic disease therapies using adenoviral vectors. Hemophilia A and B gene therapy has been investigated since the 1990’s (High, 2003), they are X-linked genetic diseases caused by mutations in the coagulation factors XIII and IX genes, respectively. The portal infusion of a first-generation Ad with the canine factor IX gene transiently corrected (1-2 months) the canine hemophilia B (Kay et al., 1994). However, longer expression (5 months) of the beta domain of factor VIII was observed after lower doses of Ad administration to correct mice hemophilia A (Connelly et al., 1996). In following studies using a HC-Ad, the correction of canine hemophilia B and A without toxicity or thrombocytopenia was obtained (Chuah et al., 2003; Ehrhardt et al., 2003). Interestingly, mice neonatal gene therapy to express factor VIII lasted for one year, even with the quick decline of its levels. Despite re-administration of the HC-Ad was well tolerated, immunity to adenovirus persisted (Hu et al., 2011). An option for long-term expression of these genes was the use of a transposase for the therapeutic gene integration. For hemophilia B, a HC-Ad stabilized through the Sleepy beauty transposase (SB) showed sustained expression of human coagulation factor IX for more than six months in mice (Yant et al., 2002). A hyperactive SB (SB100X) corrected hemophilia B in mice and canine models by somatic integration in the liver (Hausl et al., 2010). 

Only one clinical study used a HC-Ad expressing the B domain of factor VIII under albumin promoter for liver-specific expression. The study was stopped because the first patient developed systemic side effects, probably due to the high production of inflammatory cytokines and the factor VIII levels were about 1% (Mannucci, 2002). 

Regarding CF gene therapy, in a CFTR-knockout mouse model, the *in-uterus* expression of *Cftr* mediated by an Ad vector did not improve the survival of the animals (Davies et al., 2008). One of the problems is that CAR localizes to the basal membrane of the airway epithelium, thus limiting the capacity of the Ad vector to transduce the target cells (Walters et al., 1999). The use of lysophosphatidylcholine (LPC) formulation during the application of HC-Ad to the lung facilitated access to CAR and improved the gene transfer efficiency of mice, pigs, and ferrets’ epithelia. (Yan et al., 2015; Cao et al., 2018). In another study, the treatment with the pharmacological drug cyclophosphamide was shown to overcome the immunological response to HC-Ad-CFTR allowing the sustained expression of *Cftr* when the vector was repeatedly delivered to the mouse airways. The treatment reduced the expression of T cells and their infiltration into mouse lung tissues, as well as adenovirus antibody and neutralizing activity (Cao *et al.,* 2020). The incorporation of the transposons piggyBac into the HC-Ad led to efficient expression of the transgene in pig´s lungs (Cooney et al., 2018). In 2015 the largest clinical trial liposomes-mediated delivery of the *CFTR* gene showed a modest stabilization of the lung function but not sufficient to improve lung function. Consequently, development of efficient vectors that are able to transduce lung cells and animal models for CF gene therapy are still needed (Alton et al., 2015; Yan Z *et al.,* 2019).

Gene therapy using adenovirus has been attractive for the treatment of liver diseases because of the many metabolic functions of the liver, the hepatocyte Ad tropism, and the high capacity to produce and secrete proteins in circulation (Maestro et al., 2021). In a model of neonatal bovine citrullinemia, an inborn error of metabolism caused by the deficiency of argininosuccinate synthetase (*ASS*) that leads to hyperammonemia, the systematic administration of a first-generation Ad human ASS allowed the liver transduction and partially corrected the defect (Lee et al., 1999). The deficiency of ornithine transcarbamylase (OTC) is another liver disease, X-linked, associated with the urea cycle that leads to hyperammonemia encephalopathy. A mouse model with an earlier Ad vector and CMV promoter corrected the Otc deficiency for two months (Ye et al., 1996). Combining HC-Ad, specific tissue promoter, and post-transcriptional enhancement sequences allowed overexpression of *Otc* and long-term correction of the deficiency in mice without toxicity (Mian et al., 2004). However, after the first clinical trials for OTC and the fatal outcome described above (Raper et al., 2003), no other clinical trials for OTC with adenovirus have been conducted.

Adenoviral vectors, more specially HC-Ads, are widely used as experimental therapeutic vectors, but in clinical trials for genetic diseases, most promisor gene therapy is by using adeno-associated virus or lentiviral vectors. Several phases 1, 2 and 3 clinical trials for replacement therapy have been concluded or are ongoing for hemophilia (Perrin et al., 2019; Batty and Lillicrap, 2021), CF (Guggino and Cebotaru, 2020), Pompe (Unnisa et al., 2022) and other diseases (see ClinicalTrials.gov).

### *Ex-vivo* and gene editing

The *ex-vivo* gene therapy consists of modified cells outside the body to express a therapeutic gene and subsequently implant them back into patients. This therapy has been useful for inherited rare blood disorders e.g beta-thalassemia, sickle cell disease (SCD), and other hematological diseases. In these cases, the patient’s hematopoietic stem cells (HSC) are collected, transduced with a vector carrying the therapeutic gene and injected back into the patients (Tambuyzer et al., 2020). This technique has several challenges including insufficient HSC obtained from the patient, genotoxicity and limitations of the viral vectors, loss of HSC multipotency during *ex vivo* manipulation, reduced number of transduced cells for reimplantation, technical complexity and high cost (Li and Lieber, 2019; Telen et al., 2019). However, in the last years great advances in this area have been made.

The *ex-vivo* Ad transduction into human conjunctival epithelium and cornea, showed sustained expression of reporter genes, interleukin 10 and others, suggesting that this strategy could be employed to suppress immune-mediated disorders (Oral et al., 1997; Shen et al., 2001; Qian et al., 2004). For Sickle cell disease (SCD), a monogenic disorder caused by a mutation in the beta-globin gene (beta S allele) compromising the production of normal adult hemoglobin (Vichinsky et al., 2000), gene therapy approaches include the *ex-vivo* transduction of the HSPC for expressing the intact beta-globin gene, anti-sickling beta-globin, or the fetal gamma-globin. In mice models, *ex-vivo* HSPC transduction of a HC-Ad5/35 vector carrying SB100x transposase-mediated gamma-globin gene and transplantation into irradiated mice reached 95% of gamma-globin-positive peripheral red blood cells (Wang et al., 2020). This result complemented the *in vivo* model that resulted in an incomplete correction of the thalassemia phenotype in mice (Wang H *et al.,* 2019; Wang *et al.,* 2020). 

Gene-addition strategies have been optimized over the past few decades and the genome editing tools based on clustered regularly interspaced short palindromic repeats (CRISPR), transcription activator-like effector nucleases (TALENs) and zinc-finger nucleases (ZFNs) are being widely used for modifying HSC and other cells genome for gene therapy (Maggio et al., 2016; Yin et al., 2017; Stephens et al., 2019; Bandara et al., 2021). These strategies precisely target the gene of interest and can fix or “cure” the disease. However, a hurdle to be overcome is the off-target activities on unintended sites. A study showed a low scarless homology-directed genome editing of the modified cells by applying these nucleases together with an Ad donor DNA delivery compared with lentiviral or non-viral vectors templates (Holkers et al., 2014). 

The reactivation of the fetal hemoglobin HbF coded by the gamma-globin gene by knocking out its repressor BCL-11A (Brendel et al., 2016; Li et al., 2018a) or the binding sites in the globin gene (Traxler et al., 2016), and the correction of the beta S mutation has been the strategy for the CRISPR/Cas9-mediated gene therapy for SCD (Dever et al., 2016). Recently, a HC-Ad5/35 vector expressing the CRISPR/Cas9 platform (Li *et al.,* 2018b) repressed the binding region within the gamma-globin promoter after transduction of HSPCs from a thalassemic mice models (Li *et al.,* 2021). The transplantation of the modified HSCs into the irradiated animal as well as the *in vivo* intravenous injection of the vector into the mice showed efficient target site disruption and relevant switch from human beta- to gamma-globin expression that was sustained after a secondary transplantation of HSPCs, without observed hematological abnormalities in the long-term follow up (Li *et al.,* 2021).

In an *ex-vivo* approach for hemophilia B, a HC-Ad5 vector containing an inducible gene-specific CRISPR/Cas9 system together with an adeno-associated virus containing the modified donor, and a HC-Ad5 vector with all the components were used to transduce liver cell lines stably expressing mutated canine factor IX gene (carrying a point mutation). Interestingly, the single vector showed 6% of efficiency, which was superior to the two-vector strategy, thus CRISPR/CAS9 viral vector delivery is promising for the correction of mutated factor IX in disease models (Gao et al., 2019). 

Despite some hurdles, advances in *in-vivo*, *ex-vivo*, and genome edition using adenovirus-delivery as a single vector (Palmer et al., 2020) or combined (Lino et al., 2018) with other vectors are promising for genetic diseases gene therapy and may provide more gene therapy products in the near future.

## Adenovirus in cancer gene therapy

**Figure 2 -:**
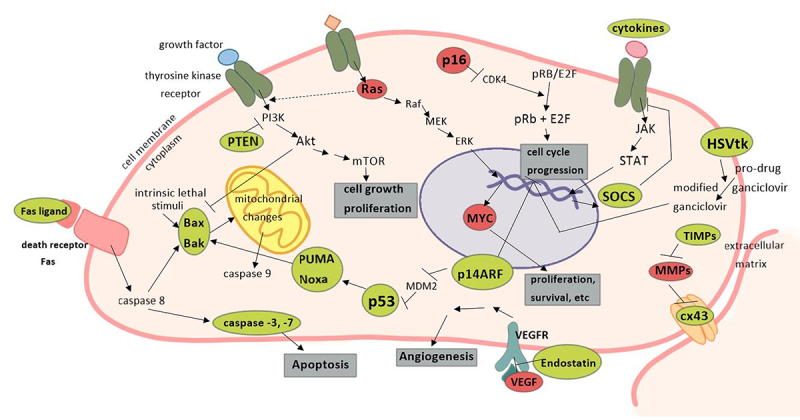
Adenovirus gene therapy targets in cancer.** **The use of adenovirus in cancer gene therapy has employed several molecular targets involving important cellular pathways that regulate cell growth, proliferation, cell cycle, survival, angiogenesis, etc. Here, we point out examples of induced (green) and downregulated (red) genes by adenovirus in cancer therapy. Phosphatase and tensin homolog (PTEN); Phosphoinositide 3-kinases (PI3K); protein kinase B (AKY); mammalian target of rapamycin (mTOR); p53 upregulated modulator of apoptosis (PUMA); Mitogen-activated protein kinase (MEK); Extracellular signal-regulated kinases (ERK); murine doble minute 2 (MDM2); Cyclin-dependent kinase 4 (CDK4); retinoblastoma protein (pRB); vascular endothelial growth factor (VEGF); vascular endothelial growth factor receptor (VEGFR); Janus kinases (JAK); signal transducer and activator of transcription proteins (STAT); Suppressor of cytokine signaling (SOCS); herpes simplex virus thymidine kinase (HSVtk); tissue inhibitors of metalloproteinases (TIMPs); metalloproteinases (MMPs); connexin 43 (cx43).

Cancer is a disease characterized by genetic alterations, uncontrolled cell functions, and loss of original cell characteristics (Hanahan and Weinberg, 2000). In 2020, it was estimated 19.3 million new cases and 10 million cancer deaths worldwide (Sung et al., 2021), it is considered one of the leading causes of death in the world (Bray et al., 2021). Despite all acquired knowledge, cancer treatment is still a challenge for several types of tumors (Wang et al., 2018). Conventional therapy based on chemo- and radiotherapy alone are not always successful (Wang *et al.,* 2018). Hanahan and Weinberg have described the hallmarks of cancer and each one of them is a relevant factor in tumor development and are key targets for cancer therapy (Hanahan and Weinberg, 2000, 2011; Hanahan, 2022). Gene therapy using adenoviral vectors appears to be an interesting option for treating cancer as it can restore or inhibit pathways that were lost or modified during tumorigenesis (Sun *et al.,* 2019a). Most of the approaches described in this section employed first generation adenoviral vectors or oncolytic adenoviruses. In Figure 2 we show a summary view of different approaches of gene therapy in cancer treatment. 

### Targeting cell proliferation and growth suppressors evasion

One of the most remarkable tumor cell characteristics is the ability of uncontrolled proliferation (Hanahan and Weinberg, 2011). Modulation of pathways and genes that control processes involved in cell cycle, proliferation, growth, and survival are commonly seen in cancer, mainly related to tumor suppressors’ inhibition and oncogenes activation (Park et al., 2020). 

The search for reestablishing phosphatidylinositol 3-kinase/protein kinase B (PI3K/AKT) pathway normal regulation is one of the adenovirus gene therapy aims. This pathway in normal cells induces cell growth and proliferation and inhibits apoptosis (Fresno Vara et al., 2004). Different alterations contribute to PI3K/AKT pathway constitutive activation in cancer, including rat sarcoma virus proto-oncogene (*RAS*) constitutive activation, and loss of the pathway negative regulator, phosphatase and tensin homolog (*PTEN*) (Fresno Vara *et al.,* 2004; Hanahan and Weinberg, 2011; Santarpia et al., 2012; Park et al., 2020). 

Induction of *PTEN* expression mediated by adenovirus (Ad-*PTEN*) in several types of cancer demonstrated to be effective to downregulate PI3K/AKT pathway, consequently contributing to apoptosis induction, migration, and growth inhibition in tumor cell lines and tumor suppression *in vivo* (see summary data in Table S1). Nonetheless, this effect is more effective in cell lines with loss or mutated *PTEN* in comparison to tumor cells carrying WT *PTEN* (Tanaka and Grossman, 2003; Hamada et al., 1999; Tanaka *et al.,* 2005; Rosser et al., 2004). Different studies analyzed the combination of Ad-*PTEN* with other therapeutic agents to potentialize its antitumor effect. Ad-*PTEN* enhanced the doxorubicin efficacy in bladder and prostate cancer (Tanaka and Grossman, 2003; Tanaka *et al.,* 2005) and sensitized tumor cells to cisplatin (Li D et al., 2013; Wu *et al.,* 2015), docetaxel (Liu Z et al., 2012), to a PI3K inhibitor (Ren et al., 2012), radiotherapy (Pappas et al., 2007; Rosser *et al.,* 2004), to TIMP-2 (Lu et al., 2004) and caffeine (Saito et al., 2003). To provide specificity to tumor cells, *PTEN* has been conjugated to the epithelial cell adhesion molecule (EpCAM), which resulted in better antitumor effects in liver cancer *in vivo* and *in vitro* (Liu Z *et al.,* 2018). Oncolytic adenoviruses expressing *PTEN* under control of a specific promoter to prostate cancer have conferred almost complete tumor regression and high specificity in prostate cancer *in vitro* and in a murine model (Ding et al., 2012). 

Meanwhile, different studies have focused on adenoviral gene therapy for *RAS* blockage. The *RAS* gene family (*H-RAS, K-RAS,* and *N-RAS*) is one of the most altered genes in cancer (Zinatizadeh et al., 2019), which is involved in proliferation, survival, angiogenesis, and cell motility (Santarpia et al., 2012). Several strategies using adenovirus have been employed in an attempt to decrease *RAS* activity in cancer, such as the expression of neutralizing anti-*RAS* antibody (van Etten et al., 2002; Yang et al., 2016), gene silencing using antisense and small interference RNAs (siRNA) (Nakano et al., 2001; Chen et al., 2005; Zhang et al., 2006), induction of a dominant-negative mutant form of *RAS* (Senmaru et al., 1998; Watanabe et al., 2001; Stoll et al., 2005), and ribozymes against *RAS* (Irie et al., 1999; Tsuchida et al., 2000; Zhang *et al.,* 2000; Wang et al., 2002) resulting in antitumor effects *in vivo* and *in vitro* (Table S1). Combinatory treatment using cytokines and *RAS*-targeted therapy resulted in synergistic tumor inhibition, as seen in pancreatic cancer using Interferon-alpha (IFN-α) (Hatanaka et al., 2004) and in colon cancer, with Interleukin-27 (IL-27) (Lebedeva et al., 2007). Additionally, oncolytic adenovirus expressing anti-*RAS* antibody conferred specificity and high antitumor efficacy in cell lines from different cancer types (Pan et al., 2017). Another approach used for targeting tumor cells was the use of cytokine-induced killer (CIK) cells as vehicles for adenoviral delivery. CIK cells carrying adenovirus expressing anti-*RAS* antibody guaranteed tumor specificity in glioma, lung, and colon cancer (Liu et al., 2018; Lin et al., 2019; Qian et al., 2021). Although, in a liver cancer model, CIK cells delivery did not demonstrate tumor specificity and adenoviruses were detected in different organs, even though antitumor activity was achieved (Dai et al., 2021). 

The retinoblastoma pathway (pRb) has also been a target of adenovirus cancer gene therapy. pRb inhibits proliferation by direct interaction with the transcription factor, E2 promoter binding factor (E2F). It releases E2F to trigger the cell cycling when it is phosphorylated and *P16*, known to be a tumor suppressor gene, prevents pRb phosphorylation and therefore cell cycle progression (D’Arcangelo et al., 2017). *P16* is found to be mutated or deleted in different types of cancer (Yang Z et al., 2016) and the restoration of its expression mediated by adenovirus (Ad-*P16*) resulted in antitumor effect in different tumor cell lines with functional pRb protein, but none or reduced activity in cell lines with mutated or null pRb (Grim et al., 1997; Craig et al., 1998; Campbell et al., 2000) (Table S2). Ad-*P16* also increased radiotherapy efficiency in head and neck cancer (Rhee et al., 2003) but conferred chemoresistance to cisplatin and paclitaxel in a *P16*-negative bladder cancer cell line (Grim *et al.,* 1997). 

Directly modulating pRb expression, it was observed that the induction of WT pRb only had an antitumor effect in cell lines that lost the *RB* gene (Fueyo et al., 1998) or in a heterozygous *RB* (+/-) mouse (Riley et al., 1996), but had no relevant effect in cervical cancer cells with inactivated pRb caused by Human Papillomavirus (HPV) infection (Ip et al., 2001). On the other hand, the adenoviral induction of a hypo-phosphorylated pRb variant resulted in tumor suppression in WT pRb cell lines (Roig et al., 2004), demonstrating that pRb-based therapy should consider not just the presence but also functionality of pRb in the tumor. 

Besides *P16*, the same gene *locus* INK4A/ARF also encodes *P14ARF*, another tumor suppressor that leads to cell cycle arrest and indirectly promotes p53 activation (Deng et al., 2002; Agrawal et al., 2006). The induction of *P14ARF* expression by adenoviral vectors (Ad-*p14ARF*) demonstrated promising results, but the presence of the *TP53* WT gene appears to be essential for its higher antitumor efficacy (Yang et al., 2000; Deng *et al.,* 2002; Kim et al., 2004). The combination of Ad-*p14ARF* with an adenovirus expressing p53 synergistically increased the cytotoxic effect even in null *TP53* cell lines (Lu et al., 2002; Tango et al., 2002), indicating that this strategy may be a good alternative for tumors lacking p53. 

Interestingly, another relevant pathway altered in cancer is the Janus kinases/signal transducer and activation of transcription (JAK/STAT). It is activated by cytokines and can control immune signaling, growth, apoptosis, tissue repair, hematopoiesis, etc (Lin, 2010; Owen et al., 2019). STAT3 is considered an oncogene and the JAK/STAT pathway is often constitutively activated in cancer (Lin, 2010). The use of adenovirus expressing suppressors of cytokine signaling (SOCS) induces a negative feedback control leading to this pathway inactivation (Liu et al., 2013b). This strategy was effective against several types of cancer cells, in addition to improve radiosensitivity (Lin, 2010; Sugase, et al., 2018; Liu *et al.,* 2013b).

*MYC* is another important gene found frequently altered in cancer (Dang,2012), which participates in cell growth regulation (Stine et al., 2015). In tumors, *MYC* is usually amplified, leading to its constitutive activation (Stine *et al.,* 2015). Different strategies have been developed for *MYC* inhibition, such as adenovirus expressing antisense c-*MYC* (Chen et al., 2001; Xie et al., 2009) or shRNA anti-*MYC* (Li Y et al., 2013) leading to tumor inhibition *in vivo* and *in vitro* (Table S1). Other targets involved in cell proliferation and survival employed in adenovirus gene therapy include survivin inhibition (Fei *et al.,* 2008; Shen et al., 2009), Ki-67 silencing (Zheng et al., 2009; Liu J et al., 2012), and epidermal growth factor receptor (EGFR) expression (Yan et al., 2020). 

These data suggest that it is important to take advantage of altered genes that are contributing to the uncontrolled proliferation and survival phenotype. One main problem is that the same therapy is not necessarily effective against tumors harboring different alterations of a pathway or even mutations of the same gene. In this case, the status of the target gene should always be considered. 

### Inducing tumor cell death and suicide gene therapy

Evading cell death is an important tumor hallmark and loss of death regulators is frequent in cancer (Hanahan and Weinberg, 2011). Several studies have focused on restoring death activators in an attempt to induce tumor cell death. Adenovirus expressing tumor necrosis factor receptor superfamily member 6 (FAS) ligand (Ad-FASL) contributed to cell death induction in different types of tumors (Zheng et al., 2005; Sudarshan et al., 2005; ElOjeimy et al., 2006). Interestingly, the expression of caspase or pro-caspase 3 mediated by adenovirus did not have an effect on apoptosis induction in glioma (Shinoura et al., 2000), liver (Yamabe et al., 1999) and prostate cancer (Li et al., 2001). High death rates were only achieved in combination with Ad-FASL (Shinoura *et al.,* 2000) or the chemotherapy etoposide as a death stimulus (Yamabe *et al.,* 1999). In contrast, pro-caspase 7 induction resulted in cell death but only in two of five cell lines tested (Li *et al.,* 2001). Overexpression of B-Cell CLL/Lymphoma 2 (BCL-2) pro-apoptotic family members such as BCL2 Antagonist/Killer 1 (BAK) and BCL2 Associated X (BAX) also demonstrated effective antitumor capacity through apoptosis induction (Table S3) and improvement of radio- and chemotherapy sensibility after Ad administration (Arafat et al., 2000; Tsuruta et al., 2001). Nonetheless, Ad-BAK was not able to induce cell death in a breast caspase-3 deficient-cell line (Pataer et al., 2000). Exploring tumor specificity, Ad-BAX under control of vascular endothelial growth factor (VEGF) promoter conferred a higher antitumor effect under hypoxic conditions in lung cancer (Kaliberov et al., 2002). In prostate (Lowe et al., 2001) and ovarian cancer (Tai et al., 1999), Ad-BAX under control of specific promoters conferred specificity and high cytotoxicity. The effect of BAX expression was also studied in combination with IL-24 (Li *et al.,* 2010) through an adenovirus expressing the TNF-related apoptosis-inducing ligand (TRAIL) and with the chemotherapeutic agent Gemcitabine (Wack et al., 2008). In all cases, the antitumor effect was improved synergistically.

P53 is another important protein that controls cell apoptosis, inducing cell death in response to stressful stimuli, besides several other processes related to tumor suppression. P53 is the tumor suppressor most frequently mutated in cancer (Bieging et al., 2014) and its restoration has been extensively used in gene therapy mediated by adenovirus (Ad-P53) in different types of cancer (reviewed before by Tazawa et al., 2013). In prostate cancer, for example, several studies have employed Ad-P53 gene therapy and showed antitumor activity (Tamura et al., 2018). Adenovirus expressing P53 under control of a P53-responsive promoter demonstrated effective tumor suppression (Tamura *et al.,* 2016), which is potentialized when the arginine-glycine-aspartic acid (RGD) motif is incorporated in the adenoviral fiber protein, leading to a higher tumor cell death effect (Tamura *et al.,* 2017) and higher chemotherapy sensitivity (Tamura *et al.,* 2020). In colon cancer, the same strategy, Ad-P53 containing RGD and P53-responsive promoter, was only effective in P53 WT or null cell lines and in a mutant *TP53* tumor cell, the combination with IFNβ was necessary to induce cell death (Del Valle et al., 2021). Several clinical trials employed adenovirus-expressing P53 for treating different types of cancer and for safety confirmation (See on clinicaltrials.gov). Currently in 2002, a phase II clinical trial is combining Ad-P53 with an approved immune checkpoint inhibitor in a cohort of 40 head and neck cancer patients and other tumors. 

Such studies resulted in Gendicine® (Shenzhen SiBiono GeneTech, Guangdong, China), an adenoviral p53 gene therapy approved in China for treating Head and neck cancers in 2003. It was also the first approved gene therapy drug, which confers higher survival rates and improved treatment compared to conventional therapies (radio and chemotherapy) with no severe side effects (Zhang et al., 2018a). Besides head and neck, gendicine can also be used for treating lung, ovarian, liver and other cancers (Zhang *et al.,* 2018a). Other two adenoviral vectors expressing p53 have been investigated by pharmaceutical companies, Advexin® (Introgen Therapeutics, Multivir, Inc, both of Houston, TX) and SCH58500 (Merck & Co, Schering-Plough, Kenilworth, NJ). 

Downstream targets of P53 have been investigated as well, including adenoviral vectors expressing P53 upregulated modulator of apoptosis (PUMA) and NADPH oxidase activator (NOXA), other two members of the BCL-2 family that participate in P53-mediated apoptosis (Agrawal et al., 2006; Elmore, 2007), in different tumor types (Table S3).

A different method for inducing cell death is through the expression of suicide genes in tumor cells (Düzgüneş, 2019). The best described is the herpes simplex virus thymidine kinase/ganciclovir (HSVtk/GCV) system, in which HSVtk converts the prodrug ganciclovir into a nucleoside analog consequently occasioning cell cycle arrest and cell death (Beltinger et al., 1999). The use of adenovirus carrying HSVtk in tumor cells has shown a relevant antitumor effect with high cytotoxicity to ganciclovir in a variety of pre- clinical trials (Table S3). Clinical trials using adenovirus-expressing HSVtk in combination with GCV demonstrated its safety and efficacy in liver cancer (Sangro et al., 2010) and glioma patients in combination with radio- and chemotherapy (Chiocca et al., 2011; Ji et al., 2016). The system cytosine deaminase /5’-Fluorocytosine (CD/5’-FC) was also explored in adenovirus gene therapy. In this case, CD converts the prodrug 5’-FC into a toxic molecule (Düzgüneş, 2019). The combination between the systems TK and CD carried to tumor cells by adenoviruses led to a synergistic antitumor effect in gastric cancer (Luo et al., 2012). In pancreatic cancer preclinical studies, AdHSVTk/CD increased the radiotherapy effect (Freytag et al., 2007) and a phase I clinical trial demonstrated tolerability in combination with gemcitabine chemotherapy (Lee et al., 2020). HSVtk/GCV and CD/5’-FC in prostate cancer also appeared to be safe in phase I clinical trials (Freytag *et al.,* 2003; Barton et al., 2008). 

Restoring the expression of tumor suppressors or inducing cell death by different means is essential in any strategy to destroy the tumoral cell mass. Therefore, it is natural that the first available product and several clinical trials assays are intended to promote direct tumor cell death and recruitment of the immune system to eliminate any remaining cells.

### Inhibiting angiogenesis

Angiogenesis is the construction of new blood vessels coming from pre-existing vessels, which is induced by tumor cells signaling and essential for tumor growth and metastasis dissemination (Chen et al., 2000). The most utilized anti-angiogenic proteins in gene therapy are statins as endostatin and angiostatin (Chen *et al.,* 2000). Both molecules are natural fragments of larger proteins (endostatin from XVIII collagen and angiostatin from plasminogen) and their anti-angiogenic capability may be due to VEGF downregulation, a well-known molecule responsible for angiogenesis induction (Hajitou et al., 2002). Different studies evaluated the antitumoral ability of adenovirus expressing endostatin, angiostatin, and different fragments of plasminogen, demonstrating to be effective against the angiogenic phenotype of endothelial cells *in vitro* and tumor suppression *in vivo*, influencing mainly tumor vessels formation, cell migration, invasion, and metastasis (Table S4). 

Several mechanisms using adenovirus were proposed to decrease VEGF expression in cancer: antisense-VEGF (Im et al., 2001), soluble forms of VEGF receptor (VEGFR/Flt-1 or VEGFR2/Flk-1) (Kong et al., 1998; Takayama et al., 2000; Hoshida et al., 2002; Yoshimura et al., 2004; Schmitz et al., 2005; Wu et al., 2006) and the vascular endothelial growth inhibitor (VEGI) fused with endostatin (Pan et al., 2004) are examples of molecules used in gene therapy that showed a reduction on neovascularization, increase in apoptosis and tumor suppression *in vivo* (Table S4). 

In addition, hepatocellular growth factor (HGF) plays a role in tumor malignant phenotype (Saimura et al., 2002). Several studies using Ad carrying its antagonist Nk4 showed anti-proliferative and anti-angiogenic activity in different types of cancer (Table S4). Other examples of adenoviral gene therapy focusing on antiangiogenic mechanisms includes the expression of pigment epithelium-derived factor (PEDF) (Mahtabifard et al., 2003; Wang et al., 2003; Merritt et al., 2004; Guan et al., 2007); endothelium-specific receptor tyrosine kinase (Tie2) (Lin et al., 1998; Popkov et al., 2005); fragments and alterations of thrombospondin 1 (Liu et al., 2003); angiotensinogen (Bouquet et al., 2006); human 16k PRL (Nguyen et al., 2007); amino-terminal fragment of urokinase (ATF) (Li *et al.,* 1999); fibroblast growth factor receptor (FGFR) (Compagni et al., 2000) and platelet factor 4 (PF4) (Tanaka et al., 1997). 

All mechanisms mentioned above indicated that gene therapy using antiangiogenic molecules provides high anticancer efficacy in pre-clinical assays, resulting in tumor growth suppression in almost all cell lines and *in vivo* models studied. It is also important to note that this strategy does not depend on a specific mutation or is restricted to a specific type of cancer. The expression of pro-angiogenic factors and stimulation of tumor blood vessel formation are frequently found in cancer, being an interesting target for cancer treatment of solid tumors. 

### Focusing on invasion and metastasis

The tumor malignant phenotype is also characterized by adjacent tissue invasion and metastasis to distant sites (Jiang et al., 2015a). These mechanisms are regulated mainly by the degradation of molecules responsible for cell to cell and cell to matrix adhesion, stimulus of cell migration, and through epithelium-mesenchymal transition (EMT) (Jiang *et al.,* 2015b). Adenoviruses expressing extracellular matrix (ECM) compounds like connexin 43 (Cx43) (Liu et al., 2015) or downregulating CD44 via short hairpin (sh) RNA (Lee et al., 2017) contributed to the reduction in invasiveness capability in cancer cells *in vitro* (Table S5). Additionally, several studies evaluated different mechanisms to inhibit matrix metalloproteinases (MMPs) that are responsible for ECM degradation. Using adenovirus expressing siRNA against MMP2 (Chetty et al., 2006; Tsung et al., 2008) or a ribozyme against MMP-13 mRNA (Ala-Aho et al., 2004) resulted in its downregulation in tumor cells, consequently leading to reduced invasion and migration. Natural inhibitors of MMPs, like tissue inhibitors of MMPs (TIMPs), were also explored. Adenovirus expressing TIMP-1, -2, or -3 demonstrated high antitumor effect mainly by reducing angiogenesis, invasion, and metastasis (Table S5). Other different methods for MMPs inhibition include the expression of cystatin C (Kopitz et al., 2005) and the urokinase plasminogen activator receptor (uPAR) (Lakka et al., 2001, 2003; Rao et al., 2005). 

Focusing on EMT as a therapeutical target, one important protein is Mothers against decapentaplegic homolog 4 (Smad4), which is involved in cell differentiation and is found mutated in several cancers (Duda et al., 2003; Xiao et al., 2020). Its overexpression mediated by an adenoviral vector in pancreatic tumor cells did not affect proliferation *in vitro* but resulted in tumor growth and angiogenesis inhibition *in vivo* (Duda *et al.,* 2003). In colon cancer, using oncolytic adenovirus, Smad4 expression promoted cell proliferation inhibition *in vivo* and *in vitro*, and reduced spheroids formation efficiency (Xiao *et al.,* 2020).

Similar to targeting angiogenesis, invasion and metastasis are common features of cancer, seen in almost all types of tumors. Adenovirus gene therapy using key molecules involved in these processes, such as TIMPs or certain ECM compounds, seems to be another intelligent strategy for reducing the tumor malignant phenotype without limitations regarding tumor type, mutations, or alterations in important pathways that diverge among tumors. 

### Modulating immune signaling

Tumor cells are modulated by both adaptive and innate immune systems. Induction of inflammation may promote tumor progression by secretion of growth and survival signaling molecules, and other factors that contribute to tumor establishment. In contrast, by immune surveillance, immune cells can destroy cancer cells (Hanahan and Weinberg, 2011). Cancer cells can evade immune destruction in the tumor microenvironment and support pro-malignant inflammation (Hanahan and Weinberg, 2011). 

One of the aims of adenovirus gene therapy is inducing the expression of immune components in the tumor microenvironment, such as inflammatory cytokines, that can regulate important cell pathways or activate the immunologic response, consequently triggering cell death or immune-mediated destruction (Waldmann, 2018). Different cytokines are in clinical trials and some of them are already approved for cancer treatment. Although, one important implication involving cytokines in cancer therapy is the low concentration of these molecules in the tumor site and that a large quantity of systemic cytokines usually provokes high toxicity (Waldmann, 2018). The use of adenovirus to target tumor cells may be an optimist alternative to increase the efficiency of cytokine delivery in cancer and reduce systemic toxicity.

Examples of adenovirus immunotherapy include the expression of tumor necrosis factor family members (TNF), like TNFα and *TRAIL*. These molecules are capable of inducing tumor growth suppression when expressed in tumor cells by oncolytic or non-replicative adenovirus (Table S6). In some cases, oncolytic adenoviruses carrying *TRAIL* had a higher cytotoxic effect in comparison to virotherapy alone (Shim et al., 2010; Cao et al., 2011b; Yang et al., 2015; Zhou et al., 2017), and improved chemotherapy treatment in bladder cancer (Mao et al., 2014). In addition, transduction of *TRAIL* gene to mesenchymal stem cells (MSCs) in co-culture with esophageal cancer cell lines promoted tumor cell apoptosis (Li et al., 2014). 

The interferon (IFN) cytokine family is also used for cancer treatment. Induction of IFNα, -β, or -γ expression in tumor cells demonstrated a high antitumor effect in several types of cancer *in vivo* and *in vitro* (Table S6). Interestingly, treating cancer with Ad-IFNα resulted in higher IFNα concentration in tumors than in systemic circulation (Ohashi et al., 2005), and promoted regression of non-treated distant tumors as well, also inducing T-cells and natural killer cells recruitment to tumor site (Hara et al., 2007). IFNβ in an oncolytic adenovirus improved treatment (He et al., 2008; Park et al., 2010) and adenovirus expressing IFNγ showed low systemic toxicity (Xie et al., 2013; Zhao et al., 2007). 

Interleukins, such as IL-24, induce apoptosis and suppress growth in several tumor types (Chang et al., 2011). Ad-IL-24 promoted tumor suppression (Chang *et al.,* 2011) and had its antitumor effect enhanced by radiotherapy in nasopharynx and breast cancer (Liu et al., 2013a; Zhao et al., 2013). Furthermore, combination of IL24 and Oncostatin M (OSM) increased antitumor activity in comparison to isolated treatment in melanoma (Xu et al., 2014) and liver cancer, combining two different oncolytic adenoviruses expressing IL-24 or SOC3S resulted in higher tumor suppression when compared to alone treatments or with an empty oncolytic adenovirus (Cao et al., 2011a). 

IL-12 is another important cytokine that acts as an important mediator for cancer immune destruction as it can activate NK and T cells, but it is toxic when administered systemically (Mirlekar and Pylayeva-Gupta, 2021). Several studies using adenovirus encoding IL-12 alone demonstrated a potent antitumor effect in pre-clinical and clinical trials (reviewed before by Hernandez-Alcoceba et al., 2016). The combination of IL-12 oncolytic adenovirus in CIK cells in liver cancer generated higher cytotoxic effect than each separated treatment (Yang et al., 2012) and combination with a TGFβ inhibitor in melanoma cells promoted increased antitumor immune response as well, leading to CD4+, CD8+ T and NK cells activation and IFNγ secretion in the tumor site (Jiang et al., 2017). Importantly, the combination of IL-12 with suicide gene therapy, such as HSVtk/GCV and CD/5’-FU seems to enhance the antitumor effect in pre-clinical and clinical studies in comparison to suicide gene therapy or IL-12 alone, increasing the presence of IL-12, IFNγ, in serum and tumor and inducing a specific antitumor immune response by NK cells and cytotoxic T cells activation in mouse model and in a phase I clinical trial (Freytag et al., 2013; Barton et al., 2021). Moreover, using IL-12 oncolytic adenovirus with selective replication in hypoxic conditions generated better antitumor response against pancreatic cancer in comparison to non-replicative adenovirus (Bortolanza et al., 2009). 

Differently from IL-24 and IL-12, IL-2 has already been approved by the Food and Drug Administration (FDA) for cancer treatment. It has also been demonstrated to be effective when delivered by an adenoviral vector in breast cancer using a specific promoter (Chaurasiya et al., 2016). Other interleukins have also been tested in gene therapy against cancer, such as IL-15 oncolytic adenovirus in breast carcinoma (Yan Y et al., 2019), and AdIL-3 in prostate cancer in combination with radiotherapy (Oh et al., 2004).

Several clinical trials evaluated the use of adenoviruses expressing cytokines for cancer treatment. A phase I clinical trial using Ad-IL12 for advanced digestive tumors demonstrated low toxicity, but only 29% of the patients presented disease stabilization and partial remission of the tumor in one patient. In addition, tumor immune infiltrate (CD4+ and CD8+ T cells) was observed in four of ten patients (Sangro et al., 2004). In advanced cancer patients, Ad-IL-24 was able to induced apoptosis in all tumors (Tong et al., 2005). 

Using IL-2, adenoviral gene therapy in prostate cancer patiets was well tolerated; inducing tumor lymphocytic infiltration, increase in IFNγ and IL-4 secretion within the tumor, and decrease in prostate specific antigen (PSA) levels (Trudel et al., 2003). In melanoma and other solid tumors patients, Ad-IL-12 also induced tumor lymphocytic infiltration (Dummer et al., 2008). Another phase I study demonstrated safety, no severe adverse side effects and no presence of systemic IL-2. However, only 24% of the metastatic breast cancer and melanoma patients resulted in tumor regression and tumor lymphocytic infiltration (Stewart et al., 1999). Additionally, an oncolytic adenovirus expressing TNFα and IL-2 is being currently (2022) tested in phase I clinical trials (See in clinicaltrials.gov). 

Different approaches for inducing an immune response against tumor cells using adenoviral vectors include the expression of CD40 ligand to promote the activation of adaptive immune response (Hanyu et al., 2008; Vardouli et al., 2009; Iida et al., 2010); NF-κB inhibition through the expression of its inhibitor, IκBα (Sumitomo *et al.,* 1999; Mukogawa et al., 2003); and the expression of pathogen-associated molecular patterns (PAMPs) to trigger innate immune responses (Tosch et al., 2009). Oncolytic adenovirus expressing immunomodulatory genes like GM-CSF has the potential to destroy tumor cells and at the same time modulate the immune system in the tumor microenvironment, having been evaluated in clinical trials (Ranki et al., 2016).

Therapeutic targets that involve the activation of the immune response within the tumor microenvironment may contribute to the activation of important pathways that lead to immune cell death and amplification of the destruction of the tumor. The use of adenovirus as gene carriers solves the problem of systemic contamination that leads to non-desired immune effects. Adenoviruses can increase the cytokine levels within the tumor, and decrease the systemic circulation. However, the use of non-replicative adenovirus expressing cytokines alone was not always successfull, but the combination with oncolytic adenovirus, radio-, chemotherapy or other therapies potentialized the antitumor effect. 

## Use of adenoviral vectors as vaccines

Replicative adenovirus was firstly used in the 1960s as a vaccine against respiratory disease in an enteric-coated tablet to elicit immune response in the intestinal tract, this way avoiding respiratory symptoms (Couch et al., 1963). Since these vaccines showed to be safe in humans, recombinant adenovirus started to be considered as a possible tool for vaccine development against other viral infections, like Hepatitis B virus and Human Immunodeficiency virus (HIV) (Morin et al., 1987).

### Adenovirus against HIV

The first articles proposing adenoviral vectors for HIV were published in the 1990s and parallel studies on Simian Immunodeficiency virus (SIV) were performed, however, in this review, we are focusing only on HIV results. 

Initially, replicative Ad4, Ad5, and Ad7 were used to carry the sequence of HIV-1 envelope glycoprotein gene (env), or gag-protease gene (Chanda et al., 1990; Natuk et al., 1992; Natuk *et al.,* 1993). These vectors were evaluated in dog, rhesus monkey and chimpanzee models and were capable of eliciting neutralizing antibodies against HIV (Natuk *et al.,* 1992 *,* 1993; Lubeck et al., 1994; Casimiro et al., 2003a). Alternatively, using the same vectors with HIV gp160 sequence, chimpanzees were protected against virus challenge (Lubeck *et al.,* 1997). Interestingly, cellular response was also achieved using non-replicative Ad5 to deliver HIV1 gag gene, a safer vaccine model (Casimiro *et al.,* 2003b). After this, replicative-defective adenoviruses were extensively used and the results discussed next are from these first-generation vectors.

Using a vaccination protocol of DNA vaccine prime and Ad5 or Ad5/35 expressing env gene as a boost in mice, the authors observed it induced high levels of IFNγ-secreting cells (Takakura et al., 2005), neutralizing antibodies (Mascola et al., 2005) and protection against a recombinant HIV-vaccinia virus (Xin et al., 2005). Similar results were obtained using other heterologous systems, such as Ad5/poxvirus vectors with HIV gag in rhesus macaques (Casimiro et al., 2004); Ad5 or Ad7 followed by HIV gp120 protein immunization in chimpanzees (Gómez-Román et al., 2006); DNA/Ad5/protein in rhesus macaques (Vinner et al., 2006) or guinea pigs (Shu et al., 2007); DNA/Ad5/Sendai virus carrying HIV gag tested in mice and rhesus macaques (Yu et al., 2008) and lentivirus/Ad5 in mice (Asefa et al., 2010).

After tests in several animal models, the first phase I clinical trials started to show results, healthy adults were inoculated with a mixture of 4 recombinant Ad5 for 3 different clades of HIV1 (Catanzaro et al., 2006) or Ad5 with HIV-1 Clade B gag/pol/nef (Priddy et al., 2008) and there were no major concerns about safety. Around 2007 some clinical trials resulted in no protection against HIV, while other clinical trials indicated possible problems involving previous Ad5 infection, emerging evidence showed that previous exposure to adenovirus could impair vaccine efficacy (Steinbrook, 2007; Quirk et al., 2008; Sekaly, 2008; Yu et al., 2008). Even worse, Ad5 seropositive individuals vaccinated could have a more permissive environment for HIV infection (Perreau et al., 2008; Benlahrech et al., 2009; D’Souza and Frahm, 2010; Hu et al., 2014), despite some controversial results (O’Brien et al., 2009; Curlin et al., 2011; Kaner et al., 2012). In spite of the increased susceptibility to HIV infection, later analysis showed no difference in disease progression between Ad5 vaccinated and placebo groups (Fitzgerald et al., 2011). One explanation for such increased susceptibility for HIV infection in the Ad5 vaccinated individuals is that stimulus with Ad5 in preexistent Ad5-seropositive individuals may trigger expansion of a specific HIV susceptible CD4+ population with increased CCR5 expression, the co-receptor used by HIV to infect the cells (Benlahrech *et al.,* 2009) and that have a Th17-like phenotype (Hu *et al.,* 2014).

Because of the negative results, alternatives were investigated and other types of adenoviruses in animal models started to be employed (Michael 2012), for example using chimpanzee adenovirus (ChAd) (Santra et al., 2009); ovine adenovirus (OAd) (Bridgeman et al., 2009); Ad26 expressing HIV-1 Gag, Pol, and Env antigens (Barouch et al., 2010); Ad4-Env (Alexander et al., 2013) or even edited/mutated Ad5 (Gabitzsch et al., 2009; Hidajat et al., 2010). Other prime-boost regimens were developed, like combinations of Bacillus Calmette-Guérin (BCG)/OAd/poxvirus (Hopkins et al., 2011); Ad26/Ad35 (Barouch *et al.,* 2012; Kaufman et al., 2012); ChAd/DNA/modified Vaccinia virus Ankara (MVA) (Roshorm et al., 2012); Ad35/MVA (Ratto-Kim et al., 2012). Additionally, second-generation adenoviruses were also used, based on Ad5 (Thomas et al., 2013). The delivery route of vaccination could be an alternative as well, as respiratory aerosolization delivery (Kaufman *et al.,* 2010) or sublingual vaccination appeared to enhance CD8^+^ T cells activation, especially in mucosal sites (Appledorn et al., 2011).

Clinical trials in healthy adults were conducted for Ad35 containing multiple HIV genes (Keefer et al., 2012; Kopycinski et al., 2014; Omosa-Manyonyi et al., 2015); Ad26-Env (Baden et al., 2013, 2015; Barouch et al., 2013; Esparza, 2013); Ad5 modified with Ad48 hexon expressing HIV env (Ad5HVR48-Env) (Baden *et al.,* 2014); a heterologous system with DNA, followed by ChAd and MVA, all carrying a fusion of all HIV conserved antigens (Hayton et al., 2014); a regimen of prime-boost with Ad35/Ad5 (Fuchs et al., 2015; Crank et al., 2016; Walsh et al., 2016b); Ad26/Ad35 (Baden *et al.,* 2016) or Sendai virus/Ad35. In a phase 3 trial, DNA prime with Ad5 boost showed no efficacy in a high risk for HIV1 infection population (Nyombayire et al., 2017), however high levels of specific CD8^+^ T cells were described to be associated with a lower risk of HIV infection (Janes et al., 2017). Recently, an Ad26 expressing Env/Gag/Pol in a 2b Clinical trial failed to confer high protection against HIV, showing about 25% of vaccine efficacy (CISION PR Newswire, 2022). Several other studies with other prime-boost formulations are underway and may provide better results. 

### Adenovirus against coronavirus

Another beneficiary of adenoviral vectors development is the vaccine against coronavirus. The first adenoviral vector that provided protection against a coronavirus was tested in pigs in 1994. The non-replicative Ad5 was used to carry the glycoprotein S (Spike) of porcine respiratory coronavirus (PRCV) and elicited mucosal immunity in pigs (Callebaut et al., 1993). The animals produced neutralizing antibodies against PRCV (Callebaut and Pensaert, 1995; Callebaut *et al.,* 1996). Another adenoviral vector was constructed carrying haemagglutinin-esterase (HE) of bovine coronavirus (BCV) and tested in cotton rats; it induced systemic and mucosal immune responses after immunization (Baca-Estrada et al., 1995). In the following years, other adenoviral vectors expressing coronavirus proteins from different animal species were tested including transmissible gastroenteritis coronavirus (TGEV) (Torres et al., 1995; Torres *et al.,* 1996).

The outbreak of severe acute respiratory syndrome coronavirus (SARS-CoV-1) in 2003 stimulated researchers to find effective vaccines against coronavirus infecting humans. Pre-clinical studies in mice and rats showed potent immune responses against the nucleocapsid (N) and Spike (S) proteins of SARS-CoV-1 after their delivery by an Ad5 vector (Liu et al., 2005; Zakhartchouk et al., 2005; See et al., 2006; Shim et al., 2012). Chimpanzee adenovirus C7 (ChAdC7) vector was also tested and elicited immune response against SARS-CoV1 in mice (Zhi et al., 2006). 

In 2012, a new outbreak was caused by the Middle East respiratory syndrome coronavirus (MERS-CoV). Since then, there have been several studies showing neutralizing antibodies produced in mice immunized with human Ad5, Ad26, Ad41 or ChAd carrying the S gene (Kim et al., 2014; Guo *et al.,* 2015; Alharbi et al., 2017; Jung et al., 2018; Jia et al., 2019; Dolzhikova et al., 2020). ChAd-S was later tested in camels, the natural host of MERS-CoV, and induced production of neutralizing antibodies (Alharbi *et al.,* 2019).

ChAd vectors, including ChAdOX1 developed by researchers from Oxford University, were then redirected to be used against the new pandemic coronavirus, SARS-CoV-2 in 2020. In rhesus macaques, mice, hamster and ferret’ models, the ChAd vectors carrying the S gene were able to induce robust immune responses and protect animals from pneumonia, results that greatly contributed to fast-track vaccines to the first clinical trials (van Doremalen et al., 2020; Hassan et al., 2020; 2021a,b; Marsh et al., 2021; Bricker et al., 2021). The same effects were observed in rhesus macaques and hamsters using human Ad26 as a carrier for the S gene (Mercado et al., 2020; Tostanoski et al., 2020; He et al., 2021), and in mice and macaques using Ad5 (Wu et al., 2020; Feng et al., 2020; Kim et al., 2021; King et al., 2021). In parallel, other vectors have been tested, such as simian adenovirus types 23 and 49 (Luo et al., 2021), gorilla adenovirus 32 (Capone et al., 2021) and rhesus adenovirus type 52 (Tostanoski *et al.,* 2021).

Clinical trials were done in healthy adult volunteers using ChAd against MERS-CoV (Folegatti et al., 2020a). In order to protect from SARS-CoV-2 and prevent development of COVID19 disease, Ad5 (Zhu et al., 2020; Guzmán-Martínez et al., 2021; Wu et al., 2021) (CT1, CT8, CT12), ChAdOx-1 (Folegatti *et al.,* 2020b; Ramasamy et al., 2020), Ad26 (Tukhvatulin et al., 2021), or Ad26 and Ad5 as a prime-boost system (Logunov et al., 2020) have been employed; also ChAd was tested in health care workers (Benning et al., 2021; Havervall et al., 2021; Lee et al., 2021); Ad5 was evaluated in children above 6 years old (Zhu *et al.,* 2021); and ChAd in heart transplanted individuals (Tanner et al., 2022). Showing its safety and effectiveness.

In phase 3 clinical trials, the heterologous prime-boost system using Ad26 and Ad5 as vectors presented an efficacy of 91,6% against COVID-19 (Logunov et al., 2021); injection of Ad26 alone, showed efficacy of 81.7% against severe-critical COVID-19 after 28 days of immunization (Sadoff *et al.,* 2021). Using one dose of Ad5 alone, efficacy against symptomatic infection was 57.5% (Halperin et al., 2022).

After vaccines roll out in the real world, some safety concerns emerged in rare cases of adverse events. There is some evidence that intramuscular adenovirus application can induce thrombotic thrombocytopenia in susceptible individuals (McGonagle et al., 2021), and other similar blood disorders in rare cases after ChAd and Ad26 vaccination (Lundstrom et al., 2021; Sorensen et al., 2021; Trogstad et al., 2021; Walter et al., 2021). This started a series of researches involving adenovirus modifications and changes in the route of application, like intranasal to overcome such events.

### Adenovirus against tuberculosis and other bacteria

The adenovirus system has been tested and used against several other pathogens, not just for viruses. One example is tuberculosis (TB). For several years, alternative vaccines against TB have been studied, because the protection mediated by Bacillus Calmette-Guérin is not sufficient to control TB spread. Initially tested in mice models, recombinant adenoviral vaccines carrying immunogenic epitopes of *Mycobacterium tuberculosis* (AdAg85A) appeared to be a good option. It showed better immune protection administered intranasally when compared to BCG (Wang et al., 2004), it worked as a booster also for BCG prime alone (Santosuosso et al., 2006; Li et al., 2015), as well as when followed by modified Vaccinia virus Ankara vectors (You et al., 2012; Betts et al., 2012; Stylianou et al., 2015; Kou et al., 2018). 

Going further in other animal models that are susceptible to *Mycobacterium* infection, a recombinant adenovirus expressing multiple antigens (Ag85A, TB10.4, TB9.8 and Acr2) increased BCG protection after *M. caprae* challenge (Pérez De Val et al., 2013); in guinea pigs after *M. tuberculosis* exposure, AdAg85A boost led to increased survival compared to BCG administration alone (Xing et al., 2009). A similar result was observed in cattle using BCG as prime, AdAg85A as a booster and tested against *M. bovis* challenge (Dean et al., 2014). Interestingly, in rhesus macaques using BCG as prime and a boost of Ad5 vector caring TB antigens (M72, ESAT-6/Ag85b, or ESAT-6/Rv1733/Rv2626/RpfD) showed no enhanced protection against infection compared to BCG used alone (Darrah et al., 2019), and the same result was observed using a regimen of prime-boost strategy with ChAd3 and MVA (Vierboom et al., 2020).

Clinical trials using an Ad35 deficient vector carrying a fusion protein of three *M. tuberculosis* antigens (Ag85A, Ag85B and TB10.4) were performed on several target groups and showed to be safe in healthy volunteers (Hoft et al., 2012; Sheehan et al., 2015; Tameris et al., 2015); infants 6-9 months (Kagina et al., 2014) and in subjects with TB latent infection (Walsh et al., 2016a; van Zyl-Smit et al., 2017). In addition, a phase 1b trial using Ad5-Ag85A in healthy volunteers showed higher levels of mucosal immune cells by aerosol administration than by muscle injection (Jeyanathan et al., 2022). No results about efficacy are available yet.

The adenoviral delivery system carrying bacterial proteins has also been used in the research of vaccines for other bacteria of medical importance. Against *Bacillus anthracis*, the agent of Anthrax, an Ad5 was constructed and tested via intranasal or intramuscular in mice and rabbits showing high survival rates after challenge (Tan et al., 2003; Kasuya et al., 2005; McConnell et al., 2006; Zhang et al., 2013a; Wu et al., 2014; Krishnan et al., 2015). Ad5 was used against *Haemophilus influenza* as well and tested in chinchillas (Winter and Barenkamp, 2010), for *Leptospira interrogans* Ad5 was tested in gerbils (Branger et al., 2001), for *Listeria monocytogenes* (Christensen et al., 2013), *Pseudomonas aeruginosa* (Worgall et al., 2005) and *Yersinia pestis* (Kilgore et al., 2021) the tests were done in mice, all showing immune response activation.

### Prevention of other diseases

The possibilities for adenoviral vectors usage are endless, several other studies are underway for malaria (Ewer et al., 2015; Hollingdale et al., 2017); Ebola (Matz et al., 2019) and Marburg virus (Dulin et al., 2021), Influenza virus (Kerstetter et al., 2021), Dengue virus (Khanam et al., 2009; George and Eo, 2011), Chikungunya virus (Campos et al., 2019; Folegatti et al., 2021) and Zika virus (Bullard et al., 2020; López-Camacho et al., 2020), Hepatitis B virus (HBV) (Zhang et al., 2018b; Chinnakannan et al., 2020), Hepatitis C virus (HCV) (Agrawal et al., 2019; Hartnell et al., 2020), Human Respiratory Syncytial virus (HRSV) (Gomi et al., 2018; Cicconi et al., 2020; Williams et al., 2020), Nipah virus (NiV) (van Doremalen et al., 2019), Human Papillomavirus (HPV) (Li et al., 2016; Wu et al., 2018), Rotavirus (Xie et al., 2015) and many more. Adding to this, veterinary application, against pathogens like Foot-and-mouth disease virus (FMD) (Diaz-San Segundo et al., 2017), Rift Valley fever virus (Stedman et al., 2019), Rabies virus (Wang et al., 2019b), Rabbit hemorrhagic disease virus (RHDV) (Jiang et al., 2018), African Swine Fever virus (Lokhandwala et al., 2017), Porcine Reproductive and Respiratory Syndrome virus (Zhu et al., 2014), Feline Immunodeficiency virus (Gonin et al., 1995) and much more. In addition, for Ebola, several formulations are in advanced clinical trials and some are already approved by many health regulatory agencies, including vaccines based on Ad26, Ad5 and ChAd3 (Woolsey and Geisbert, 2021).

### Modulation of immune system by adenovirus

One interesting aspect of adenovirus usage is the capability of immune modulation by choosing the inoculation route and by virus modifications. For example, mice immunized intraperitoneally with a replication-defective adenovirus elicited an IgGa immune response against the hexon, while intravenous application triggered an antibody isotype variation (Gahéry-Ségard et al., 1997). Adenoviral intranasal immunization induced higher levels of specific IgA on airway mucosa, higher systemic IgG1/IgG2a ratio and lower levels of IFN-γ secreting cells compared to subcutaneously application (Papp et al., 1999b). Surprisingly, orally administered adenovirus, elicited systemic immune response rather than mucosal (Oomura et al., 2006). Moreover, combination of different routes can help to obtain a stronger immune response. In a study model for HRSV, a regimen of oral prime and intranasal Ad vaccine boost was able to enhance immune response compared to each individually (Fu et al., 2010).

Intramuscular injection generated high levels of CD8^+^ T cell, probably due to adenovirus’ ability to express considerable high quantities of antigens (Yang T et al., 2003) plus its costimulatory effects in antigen-presenting cells (APCs), promoting activation and maturation of dendritic cells (DC) and inducing prolonged CD8^+^ T lymphocytes activation (Rea et al., 1999; Hensley et al., 2005; Tatsis et al., 2007b). The same advantage of adenovirus to induce high expression levels can have a downside effect, this presentation for long periods can provoke T cells exhaustion, a state of dysfunctional role common in processes of chronic inflammation, fortunately, CD8^+^ T cells appeared to still work against virus challenge despite the exhausted phenotype (Yang *et al.,* 2006). There is a possibility that the exhausted T cells are related to the immunization route since systemic immunization induced impaired T cells but the same was not observed in the peripheral route (Holst et al., 2010). Furthermore, in an HBV vaccination mice model, repeated vaccination with short intervals for a long period did not inhibit T cells induction, leaving a doubt if the exhausted phenotype is really impairing immune response (Boukhebza et al., 2014).

Another aspect of adenovirus infection and immune responses is controlled by the different receptors that each subtype preferably interacts with. Most Ad types use CAR to enter cells, others CD46, a receptor presented in DC cells that contribute to its infection and together with Toll Like Receptor 9 (TLR9) activation induces these cells to produce IFN-α (Iacobelli-Martinez and Nemerow, 2007; Perreau et al., 2012). Therefore, the choice of adenovirus type is important. For example, Ad3 can be found in the liver and lung, while Ad37 in the spleen after intravenous administration; Ad3 and Ad4 can even be toxic for the liver (Appledorn et al., 2008). Additionally, responses can be type-specific, Ad28 and Ad35 are more efficient in infecting and activating DC cells than Ad5, but they also induce more IFN-α and that can reduce their effectiveness *in vivo*, which can be overcome by higher Ad doses to increase the duration of CD8^+^ T cells response (Johnson et al., 2012).

Regarding innate immunity, it is important to emphasize that it is not a single TLR that is responsible for immune activation, apparently, multiple pathways are being activated by Ad, since knock out of each TLR individually did not change CD8^+^ response but absence of Myd88, an adaptor of TLR pathway, reduced it (Rhee et al., 2011). In addition, specific activation of TLR4 is described as an important step to trigger an effective humoral immune response by Ad vectors (Li R et al., 2018). TLR4 agonists can also enhance activation of CD4^+^ and CD8^+^ T cells and pro-inflammatory cytokines when used as an adjuvant to Ad vaccination (Lebedeva et al., 2018).

Cytokine production has different modulatory activities depending on Ad type; Ad26, Ad35 and Ad48 use CD46 receptor induce more IFN-γ, 10-kDa gamma interferon-induced protein (IP-10), interleukin 1 receptor antagonist (IL-1RA) and IL-6, all related to a proinflammatory pattern, compared to Ad5, a type that uses CAR receptor (Teigler et al., 2012). 

An additional layer to consider when using adenoviral vectors is related to previous immune responses to the specific type used, since prior exposure to the Ad can interfere with its capability to induce an immune response, especially in homologous prime-boost regimens (Yang Z et al., 2003; Schuldt et al., 2012). However, usage of different types of adenoviruses can overcome this problem, as exemplified by researchers’ experiments with isotypes of defective chimpanzee adenovirus applied in a heterologous prime-boost immunization; they showed induction of a high frequency of specific CD8^+^ T cells (Pinto et al., 2003). Moreover, usage of rare Ad types can contribute to activation of phenotypically different T cells triggering polyfunctional immune responses (Liu et al., 2008). In conclusion, to use the full potential of Ad vectors, all aspects of their interaction with the immune system need to be evaluated and extensively studied, especially considering the different applications in gene therapy, cancer treatment and vaccine development.

## Conclusion

**Figure 3 -:**
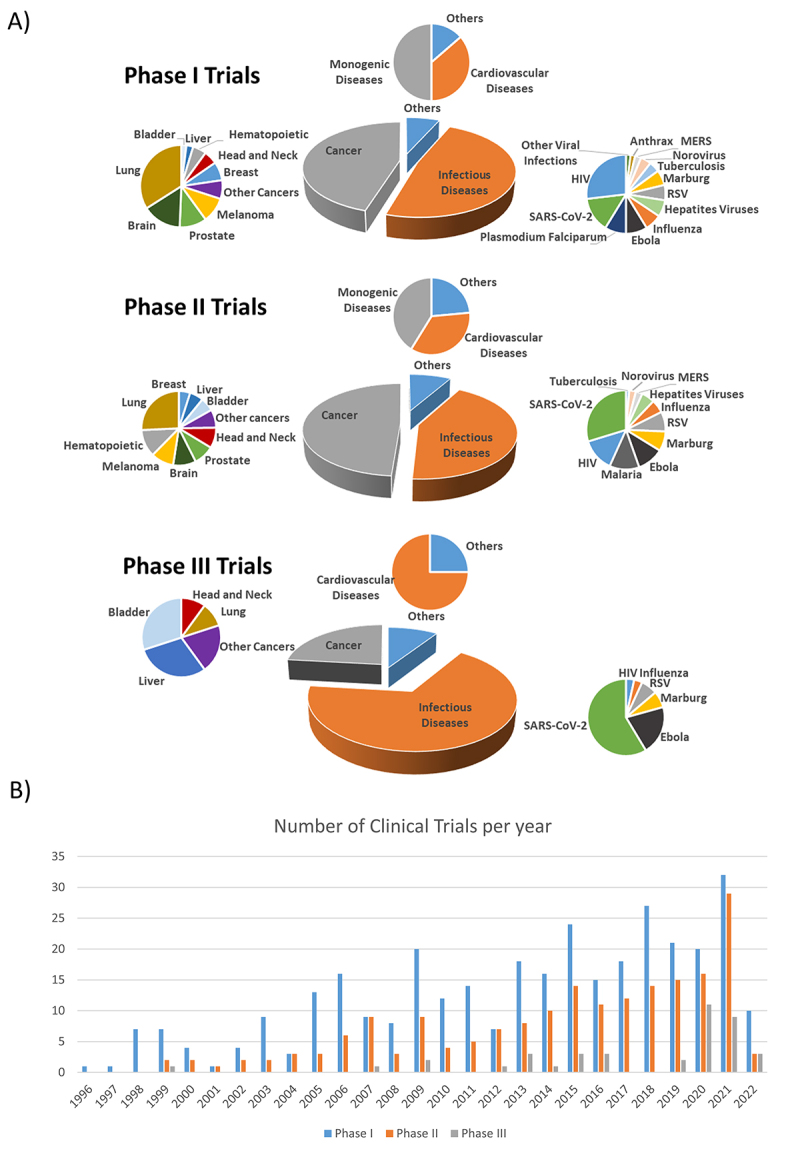
Clinical trials involving adenovirus technology. A) Proportion of ongoing and finished clinical trials of phases I, II and III divided by application on cancer treatment, infectious diseases prevention and other therapies, as of 2022. B) Number of clinical trials presented by year of beginning.

Adenovirus is a double-stranded DNA virus that does not integrate the host genome, remaining episomal. Gene transfer mediated by adenoviral vectors is not sustained for long periods, unless it has been altered to be able to integrate. This limits its application for monogenic diseases treatment. However, for cancer gene therapy, transgene expression has to last only long enough to mediate the tumor cells death, the choice of the transgene has to take into account the tumor cell and microenvironment, limiting its nutritional supply, preventing proliferation, inducing cell death and recruiting and activating immune cells capable of destroying the tumor cells and averting dissemination to other sites. The recent and broad use of adenoviral vectors as vaccination tools in the COVID-19 crisis has put this technology in the spotlight and overall, it had success, there are some issues to be solved and questions to be answered, like if the individuals vaccinated with adenoviral vectors will develop neutralizing antibodies that will impede its future use. In Figure 3 we discuss the distribution of clinical trials involving adenoviral technology. It has been a long and bumpy road along the way. But the continuous effort in this research field may warrant new successful therapies and vaccines.

## Supplementary material

The following online material is available for this article:

Table S1 - Adenoviral vectors modulating proliferation and survival of tumor cells.

Table S2 - Adenoviral vectors inducing growth suppressors.

Table S3 - Adenoviral vectors inducing cell death.

Table S4 - Adenoviral vectors modulating tumoral angiogenesis.

Table S5 - Adenoviral vectors modulating invasion and metastasis.

Table S6 - Adenoviral vectors modulating immune system.
