# Supplementary material for: The use of adenoviral vectors in gene therapy and vaccine approaches
Source: Genet Mol Biol. 2022 Oct 7;45(3 Suppl 1):e20220079. doi: 10.1590/1678-4685-GMB-2022-0079 (PMC9543183; doi:10.1590/1678-4685-GMB-2022-0079)
Supplement: Table S1 - [file 1415-4757-GMB-45-3-s1-e20220079-s1.pdf]

Genetics and Molecular Biology
https://doi.org/10.1590/1678-4685-GMB-2022-0079

Supplementary Material to "The use of adenoviral vectors in gene therapy and vaccine approaches"

Table S1 - Adenoviral vectors modulating proliferation and survival of tumor cells.

Reference	Genes	Tumor type	Cell lines/models	Results
Chen and Yang, 2010	PTEN	Lung	A549; A549 tumor-bearing mice (*in vivo*);	Reduction in cell viability and proliferation; increase of cell apoptosis; tumor growth suppression *in vivo*; increase of Bax, caspase 3 and p16 expression; decrease of CD34, VEGF and p53;
Stewart et al., 2002	PTEN	Melanoma	A375-S2, MeWo, WM35 (WT PTEN);	Apoptosis induction; growth, migration, angiogenesis, and Akt phosphorylation inhibition; E-cadherin expression increase;
Tanaka and Grossman, 2003	PTEN	Bladder	UM-UC-3 (PTEN deleted), UM-UC-6dox (WT PTEN, doxorrubicin-resistent), UM-UC-14 (WT-PTEN) tumors in nude mice;	Apoptosis induction, tumor growth suppression in UM-UC-3 and UM-UC-dox tumors; Higher tumor suppression in UM-UC-3 tumors (complete regression) in comparison to UM-UC-14 tumors (transient suppression); VEGF downregulation; decreased tumor microvessel density; enhancement of doxorubicin effect in UM-UC-dox, but not completely in UM-UC-14;
Hang et al., 2005	PTEN	Gastric	MGC-803, SGC-7901 (all WTPTEN); xenograft model in mice;	Apoptosis induction and growth suppression in tumor cell lines but not in normal gastric cell lines; Akt downregulation; MAPK and focal adhesion kinase dephosphorylation; cell cycle arrest in G2/M; tumor growth inhibition *in vivo*;
Saito et al., 2003b	PTEN	Colon	DLD-1, HT29, SW480 (all WT PTEN); xenografts tumors in mice (HT-29 and SW480);	Cell growth suppression, apoptosis induction in colon tumor cells but not in normal fibroblast cells; Akt downregulation; focal adhesion kinase and MAPK dephosphorylation; G2/M cell cycle arrest; tumor suppression *in vivo*;
Hamada et al., 1999	PTEN	Endometrial	AN3CA (WT PTEN), HEC1-A (WT PTEN), KLE (WT PTEN), RL95-2 (mutated PTEN), SK-UT-1B (mutated PTEN), Ishikawa 3 H 12 (mutated PTEN);	Growth suppression and apoptosis induction in Ishikawa 3 H 12 and RL95-2; tumor formation inhibition *in vivo* in Ishikawa 3 H 12; No effect in all different cell lines *in vivo*.
Minaguchi et al., 1999	PTEN	Ovarian	Caov-3, ES-2, MDAH 2774, NIH:OVCAR-3, OV-1063, SK-OV-3, SW 626, MCAS, TYK-nu (all WT PTEN);	Proliferation decreased only in MDAH 2774, TYK-nu, SW 626, NIH:OVCAR-3, OV-1063 and SK-OX-3;
Zhou et al., 2010	PTEN	Esophageal	Eca-109, TE-1; xenografts tumors in mice;	Growth suppression and apoptosis induction; BCL-2 downregulation; tumor growth inhibition *in vivo*;
Wu et al., 2006b	PTEN	Bladder	T24, 253J;	Growth suppression and apoptosis induction; survivin downregulation; caspase activation;
Ren et al., 2012	PTEN + LY294002 treatment	Breast	MCF-7;	Sensitization to LY294002; the combination had higher effect in reducing cell viability and invasion, lower AKT and GSK3β phosphorylation, decrease in nuclear β-catenin, Fra-1, Tcf-4 and c-Myc;

Reference	Genes	Tumor type	Cell lines/models	Results
Li et al., 2013a	PTEN + cisplatin	Lung	NCI-H446 (PTEN null); xenografts tumors in mice;	The combination increases the growth suppression, cell cycle arrest and apoptosis induction *in vitro*; higher up regulation of p53, p21, p27, Bax, and cleaved caspase 3 and downregulation of Bcl-2 and survivin *in vitro* and *in vivo*; higher reduction in CD34 expression and tumor microvessel density;
Wu et al., 2015	PTEN + cisplatin	Ovarian	CI3K mice model of human ovarian cancer;	Enhancement of proliferation inhibition, apoptosis induction and growth suppression *in vivo*;
Tanaka et al., 2005	PTEN	Prostate	PC-3, LNCaP (PTEN deleted); DU-145 (WT-PTEN);	Growth suppression in PC-3 and LNCaP, but not in DU-145; AdPTEN increased the effect of doxorubicin in PC-3 and DU-145;
Liu et al., 2012b	PTEN mannan-modified + docetaxel	Liver	murine H22 model of hepatocellular carcinoma;	Tumor growth suppression; apoptosis induction; higher death effect in combination with docetaxel in comparison with isolated treatments;
Pappas et al., 2007	PTEN + radiation	Lung	H1299	Enhancement of apoptosis induction using combinatory treatment;
Rosser et al., 2004	PTEN + radiation	Prostate	PC-3-Bcl-2 (Bcl-2 overexpression, null PTEN), PC-3-Neo (WT Bcl-2, null PTEN), LNCaP (Bcl-2 overexpression, null PTEN), BU-145 (WT Bcl-2, WT PTEN);	AdPTEN resulted in higher surviving reduction in PC-3-Bcl-2 and in LNCaP; The combination improved treatment only in PC-3-Bcl-2 cells;
Liu et al., 2018b	PTEN conjugated with EpCAM	Liver	HepG2; xenograft mouse model;	The conjugation resulted in higher anti-proliferative and anti-migratory effects; selectivity to EpCAM-positive HepG2 cells *in vivo*;
Lu et al., 2004	PTEN + TIMP-2	Glioma	U87;	The combination resulted in higher invasiveness inhibition *in vitro* than with isolated treatments;
Saito et al., 2003a	PTEN + caffeine	Colon	SW480, DLD-1 (both WT PTEN); HCT116 (p53 WT), HCT116 (negative p53) (both PTEN WT);	Synergistic effect in cell growth suppression, apoptosis induction in tumor but not normal cells; G2/M cell cycle arrest; AKT downregulation; p44/42 MAPK pathway modulation;
Ding et al., 2012	PTEN oncolytic adenovirus under control of DD3 promoter	Prostate	DU145, 22RV1, CL1, LNCaP, PC3; in comparison to non-prostate lineages: T-24, HeLa, HFL-I, L-02, WISH; 22RVI and CL1 xenograft murine models;	Apoptosis induction *in vitro*; specificity to prostate cancer lines; growth suppression *in vivo*;
van Etten et al., 2002	anti p21-Ras scFv	Colon	CC53I; liver metastasis model;	Inhibition of proliferation *in vitro*; only intra tumoral injection was effective in comparison to intravenous; growth inhibition in all animals; tumor complete regression in three of eight animals;
Pan et al., 2017	anti-p21Ras oncolytic adenovirus under control of hTERT promoter	Different types of cancer	HepG II, HCT116, MDA-MB-231, MCF-7; MDA-MB-231 tumor xenograft murine model;	Specificity to tumor cell lines; growth suppression *in vitro*; tumor growth inhibition and apoptosis induction *in vivo*;
Yang et al., 2016a	anti-p21Ras scFv	Different types of cancer	SW480, MDA-MB-231, OVCAR-3, BEL-7402 (high	Growth suppression and cell cycle arrest in cells with high expression of p21Ras; tumor growth inhibition in tumors cells expressing high levels of p21Ras *in vivo*;

Reference	Genes	Tumor type	Cell lines/models	Results
			expression of p21Ras); SKOV3 (low expression of p21Ras); BEL-7402, SW480 and SKOV3 tumor xenografts murine models;	
Lin et al., 2019	anti p21Ras scFv	Lung	A549; A549 tumor-bearing mice;	Cytokines induced killer (CIK) cells as delivery of adenovirus; growth inhibition *in vivo*, specificity to tumor site;
Liu et al., 2018a	anti-p21Ras scFv	Colon	SW480; SW480 tumor-bearing mice;	Cytokines induced killer (CIK) cells as delivery of adenovirus; antitumor effect *in vitro* and *in vivo* (Adenovirus alone or induced by CIK cells); specificity to tumor site;
Dai et al., 2021	anti-p21Ras scFv	Liver	Huh7; Huh7 xenograft mice model;	Cytokines induced killer (CIK) cells as delivery of adenovirus; adenovirus alone promoted migration, proliferation and invasion inhibition and apoptosis induction; detected in different organs *in vivo*;
Qian et al., 2021	anti-p21Ras scFv	Glioma	U251; U251 xenograft mouse-model;	CIK cells enhanced Ad-antip21 activity;
Nakano et al., 2001	K-ras antisense	Colon	HT-29, WiDr, SW1116, HCR-15; HCT-15 tumor model in SCID mice;	Reduction of K-ras p21 protein; growth suppression in cell lines with positive and negative K-ras mutation status; no effect in normal cells tested *in vitro*; growth suppression *in vivo*;
Zhang et al., 2006	siRNA K-Ras	Lung	H441 (mutated Ras); H1650 (without relevant Ras mutation); H441 tumor mice model;	Higher proliferation inhibition in H441 in comparison to H1650; inhibition of tumor establishment in mice; pre-established tumor regression;
Chen et al., 2005	siRNA K-Ras	Pancreatic	Panc-1	Apoptosis induction *in vitro*;
Stoll et al., 2005	RASN17 RAS negative dominant	Pancreatic	Panc-1, HPAF; HAPAH mouse tumor model;	Proliferation inhibition, apoptosis induction, PI3K pathway inhibition; tumor growth suppression *in vivo*;
Watanabe et al., 2001	H-Ras negative dominant	Bladder	KU-7, UMUC-2; KU-7 and UMUC-2 orthotropic mouse model;	Growth suppression, apoptosis induction *in vitro*; tumors size and number reduction *in vivo*;
Senmaru et al., 1998	mutant Ras negative dominant	Esophageal	TE8, SGF3, SGF7 (WT Ras); HEC46 (mutant RAS);	Higher sensitivity in WT Ras cell lineages; growth suppression *in vivo*;
Wang et al., 2002	H-Ras ribozyme	Laryngeal	Hep-2 (WT H-Ras); Hep-2 mouse model;	Induction of apoptosis in both extrinsic and intrinsic pathways; growth inhibition *in vivo* and *in vitro*;
Irie et al., 1999	H-Ras ribozyme	Bladder	EJ (H-ras mutation); EJ mouse model;	Tumor regression *in vivo*;
Tsuchida et al., 2000	K-Ras ribozyme	Pancreatic	Capan-1	Growth inhibition, apoptotic morphological changes, Bcl-2 inhibition;
Zhang et al., 2000	K-Ras ribozyme	Lung	H441 , H1725 (both with mutated K-Ras); H1650 (no relevant mutation); H444, H1725 tumor mouse models;	Growth inhibition in mutated cell lines but decreased effect in H1650; tumor growth suppression *in vivo*; inhibition of subcutaneous tumors establishment in mice;
Hatanaka et al., 2004	antisense K-Ras + IFNα	Pancreatic	AsPC-1, MIAPaCa-2,	Anti-sense K-Ras enhanced IFNα induced death; enhancement of growth suppression;

Reference	Genes	Tumor type	Cell lines/models	Results
			Panc-1, PSN-1, 766T; AsPC-1 mouse model;	
Lebedeva et al., 2007	anti-Ras antibody, Ras antisense + IL-24	Colon	HCT-15, HCT 1166, SW620, HT29;	Combination with IL24 increased the antitumor effect in cells with mutated K-ras, but not in WT Ras cells.
Chen et al., 2001	antisense c-Myc	Gastric	SGC7901;	Cell growth inhibition, apoptosis induction, proliferation inhibition, prevents tumor formation in mice, tumor suppression in mouse model;
Li et al., 2013b	shRNA MYCN	Neuroblastoma	LA1-55N (p53 null and MYCN amplified); LA1-55N tumor bearing mice;	Downregulation of MYCN, proliferation inhibition, tumor growth suppression *in vivo*;
Xie et al., 2009	antisense c-Myc	Osteosarcoma	MG-63;	Apoptosis induction, proliferation inhibition; enhancement of cisplatin effect; combination of Ad-c-MYC, caffeine and cisplatin have the major antitumor effect in comparison to the treatments alone;
Fei et al., 2008	ribozyme anti survivin	Liver	SMMC-772, PLC;	Apoptosis induction; tumor growth inhibition *in vivo*;
Shen et al., 2009	shRNA anti survivin	Colon	SW480;	Apoptosis induction, cell growth inhibition *in vivo* and *in vitro*;
Zheng et al., 2009	shRNA ki67 oncolytic adenovirus	Renal	786-0, ACHN;	ki67 silencing, apoptosis induction *in vitro* and *in vivo*;
Liu et al., 2012a	RNAi ki67 oncolytic adenovirus	Renal	786-0, ACHN;	Oncolytic adenovirus with G250 promoter; higher cytotoxic effect and proliferation inhibition in 786-0;
Maoxiao et al., 2020	EGFR miRNA and reverse caspase 3	Liver	HEP-2;	Cell growth inhibition, apoptosis induction, apoptosis enhancement in combination with cisplatin;

References:

Chen JP, Lin C, Xu CP, Zhang XY, Fu M, Deng YP, Wei Y and Wu M (2001) Molecular therapy with recombinant antisense c-myc adenovirus for human gastric carcinoma cells in vitro and in vivo. J Gastroenterol Hepatol (Australia) 16:22–28.

Chen L-M, Le H-Y, Qin R-Y, Kumar M, Du Z-Y, Xia R-J and Deng J (2005) Reversal of the phenotype by K-ras val12 silencing mediated by adenovirus-delivered siRNA in human pancreatic cancer cell line Panc-1. World J Gastroenterol 11:831–838.

Chen Z and Yang J (2010) Growth suppression of human lung cancer cells and implanted tumors by adenovirus-mediated transfer of the PTEN gene. J Huazhong Univ Sci Technolog Med Sci 30:149–154.

Dai F, Zhang PB, Feng Q, Pan XY, Song SL, Cui J and Yang JL (2021) Cytokine-induced killer cells carrying recombinant oncolytic adenovirus expressing p21Ras scFv inhibited liver cancer. J Cancer 12:2768–2776.

Ding M, Cao X, Xu H neng, Fan J kai, Huang H ling, Yang D qin, Li Y hua, Wang J, Li R and Liu XY (2012) Prostate cancer-specific and potent antitumor effect of a DD3-controlled oncolytic virus harboring the PTEN gene. PloS One 7:e35153.

Fei Q, Zhang H, Fu L, Dai X, Gao B, Ni M, Ge C, Li J, Ding X, Ke Y *et al.* (2008) Experimental cancer gene therapy by multiple anti-survivin hammerhead ribozymes. Acta Biochimica et Biophysica Sinica 40:466–477.

Hamada H, Yokoyama T, Furukawa T, Sato S, Yajima A, Sato M, Fujimura S and Horii A (1999) Adenovirus-mediated delivery of the PTEN gene inhibits cell growth by induction of apoptosis in endometrial cancer. Int J Oncol 15:1069-1074

Hang Y, Zheng Y-C, Cao Y, Li Q-S and Sui Y-J (2005) Suppression of gastric cancer growth by adenovirus-mediated transfer of the PTEN gene. World J Gastroenterol 11:2224–2229.

Hatanaka K, Suzuki K, Miura Y, Yoshida K, Ohnami S, Kitade Y, Yoshida T and Aoki K (2004) Interferon-α and antisense K-ras RNA combination gene therapy against pancreatic cancer. J Gene Med 6:1139–1148.

Irie A, Anderegg B, Kashani-Sabet M, Ohkawa T, Suzuki T, Halks-Miller M, Curiel DT and Scanlon1 KJ (1999) Therapeutic Efficacy of an Adenovirus-Mediated Anti-H-ras Ribozyme in Experimental Bladder Cancer. Antisense Nucleic Acid Drug Dev 9:341-349.

Lebedeva I V., Su ZZ, Emdad L, Kolomeyer A, Sarkar D, Kitada S, Waxman S, Reed JC and Fisher PB (2007) Targeting inhibition of K-ras enhances Ad.mda-7-induced growth suppression and apoptosis in mutant K-ras colorectal cancer cells. Oncogene 26:733–744.

Li D, Zhang Y, Xie Y, Xiang J, Zhu Y and Yang J (2013a) Enhanced tumor suppression by adenoviral PTEN gene therapy combined with cisplatin chemotherapy in small-cell lung cancer. Cancer Gene Ther 20:251–259.

Li Y, Zhang B, Zhang H, Zhu X, Feng D, Zhang D, Zhuo B, Li L and Zheng J (2013b) Oncolytic adenovirus armed with shRNA targeting MYCN gene inhibits neuroblastoma cell proliferation and in vivo xenograft tumor growth. J Cancer Res Clin Oncol 139:933–941.

Lin XR, Zhou XL, Feng Q, Pan XY, Song SL, Fang H, Lei J and Yang JL (2019) CIK cell-based delivery of recombinant adenovirus KGHV500 carrying the anti-p21Ras scFv gene enhances the anti-tumor effect and safety in lung cancer. J Cancer Res Clin Oncol 145:1123–1132.

Liu FR, Bai S, Feng Q, Pan XY, Song SL, Fang H, Cui J and Yang JL (2018a) Anti-colorectal cancer effects of anti-p21Ras scFv delivered by the recombinant adenovirus KGHV500 and cytokine-induced killer cells. BMC Cancer 18:1087.

Liu J, Fang L, Cheng Q, Li L, Su C, Zhang B, Pei D, Yang J, Li W and Zheng J (2012a) Effects of G250 promoter controlled conditionally replicative adenovirus expressing Ki67-siRNA on renal cancer cell. Cancer Sci 103:1880–1888.

Liu Z, Li J, Li J, Huang J, Ke F, Qi Q, Jiang X and Zhong Z (2012b) Mannan-modified Ad5-PTEN treatment combined with docetaxel improves the therapeutic effect in H22 tumor-bearing mice. Int J Nanomedicine 7:5039–5049.

Liu Z, Sun X, Xiao S, Lin Y, Li C, Hao N, Zhou M, Deng R, Ke S and Zhong Z (2018b) Characterization of aptamer-mediated gene delivery system for liver cancer therapy. Oncotarget 9: 6830-6840.

Lu W, Zhou X, Hong B, Liu J and Yue Z (2004) Suppression of invasion in human U87 glioma cells by adenovirus-mediated co-transfer of TIMP-2 and PTEN gene. Cancer Lett 214:205–213.

Maoxiao Y, Chen J, Hua J, Yuqiong X, Chunchun L, Lihong C, Beibei Y, Jiang C (2020) Effective inhibition of cancer cells by recombinant adenovirus expressing EGFR-targeting artificial microRNA and reversed caspase-3. PLoS One 15:1-13.

Minaguchi T, Mori T, Kanamori Y, Matsushima M, Yoshikawa H, Taketani Y and Nakamura Y (1999) Growth Suppression of Human Ovarian Cancer Cells by Adenovirus-mediated Transfer of the PTEN Gene. Cancer Res 59:6063-6067.

Nakano M, Aoki K, Matsumoto N, Ohnami S, Hatanaka K, Hibi T, Terada M and Yoshida T (2001) Suppression of colorectal cancer growth using an adenovirus vector expressing an antisense K-ras RNA. Mol Ther 3:491–499.

Pan XY, Liu XJ, Li J, Zhen SJ, Liu DX, Feng Q, Zhao WX, Luo Y, Zhang YL, Li HW *et al.* (2017) The antitumor efficacy of anti-p21Ras scFv mediated by the dual-promoter-regulated recombinant adenovirus KGHV300. Gene Ther 24:40–48. doi: 10.1038/gt.2016.74

Pappas G, Zumstein LA, Munshi A, Hobbs M and Meyn RE (2007) Adenoviral-mediated PTEN expression radiosensitizes non-small cell lung cancer cells by suppressing DNA repair capacity. Cancer Gene Ther 14:543–549

Qian J, Yang M, Feng Q, Pan XY, Yang LL and Yang JL (2021) Inhibition of glioma by adenovirus KGHV500 encoding anti-p21Ras scFv and carried by cytokine-induced killer cells. Exp Biol Med 246:1228–1238.

Ren Y, Zhou X, Qi Y, Li G, Mei M and Yao Z (2012) PTEN activation sensitizes breast cancer to PI3-kinase inhibitor through the β-catenin signaling pathway. Oncol Rep 28:943–948.

Rosser CJ, Tanaka M, Pisters LL, Tanaka N, Levy LB, Hoover DC, Barton Grossman H, McDonnell TJ, Kuban DA and Meyn RE (2004) Adenoviral-mediated PTEN transgene expression sensitizes Bcl-2-expressing prostate cancer cells to radiation. Cancer Gene Ther 11:273–279.

Saito Y, Gopalan B, Mhashilkar AM, Roth JA, Chada S, Zumstein L and Ramesh R (2003a) Adenovirus-mediated PTEN treatment combined with caffeine produces a synergistic therapeutic effect in colorectal cancer cells. Cancer Gene Ther 10:803–813.

Saito Y, Swanson X, Mhashilkar AM, Oida Y, Schrock R, Branch CD, Chada S, Zumstein L and Ramesh R (2003b) Adenovirus-mediated transfer of the PTEN gene inhibits human colorectal cancer growth in vitro and in vivo. Gene Ther 10:1961–1969.

Senmaru N, Shichinohe T, Takeuchi M, Miyamoto M, Sazawa A, Ogiso Y, Takahashi T, Okushiba S, Takimoto M, Kato H *et al.* (1998) Suppression of Erk Activation and in vivo growth in esophageal cancer cells by the dominant negative Ras mutant, N116Y. Int J Cancer 78:366-71

Shen W, Wang CY, Wang XH and Fu ZX (2009) Oncolytic adenovirus mediated Survivin knockdown by RNA interference suppresses human colorectal carcinoma growth in vitro and in vivo. J Exp Clin Cancer Res 28:81.

Stewart AL, Mhashilkar AM, Helena Yang X, Ekmekcioglu S, Saito Y, Sieger K, Schrock R, Onishi E, Swanson X, Mumm JB *et al.* (2002) PI3K Blockade by Ad-PTEN Inhibits Invasion and Induces Apoptosis in Radial Growth Phase and Metastatic Melanoma Cells.

Stoll V, Calleja V, Vassaux G, Downward J and Lemoine NR (2005) Dominant negative inhibitors of signalling through the phosphoinositol 3-kinase pathway for gene therapy of pancreatic cancer. Gut 54:109–116.

Tanaka M and Grossman HB (2003) In vivo gene therapy of human bladder cancer with PTEN suppresses tumor growth, downregulates phosphorylated Akt, and increases sensitivity to doxorubicin. Gene Ther 10:1636–1642.

Tanaka M, Rosser CJ and Grossman HB (2005) PTEN gene therapy induces growth inhibition and increases efficacy of chemotherapy in prostate cancer. Cancer Detect Prev 29:170–174.

Tsuchida T, Kijima H, Hori S, Oshika Y, Tokunaga T, Kawai K, Yamazaki H, Ueyama Y, Scanlon KJ, Tamaoki N *et al.* (2000) Adenovirus-mediated anti-K-ras ribozyme induces apoptosis and growth suppression of human pancreatic carcinoma. Cancer Gene Ther 7:373-383.

van Etten B, ten Hagen T, de Vries M, Ambagtsheer G, Huet T and Eggermont A (2002) Prerequisites for effective adenovirus mediated gene therapy of colorectal liver metastases in the rat using an intracellular neutralizing antibody fragment to p21-Ras. Br J Cancer 86:436–442.

Wang C-H, Tsai L-J, Tsao Y-P, Hsieh J-T, Chien W-W, Liao C-L, Wang H-W, Liu H-S and Chen S-L (2002) Recombinant adenovirus encoding H-ras ribozyme induces apoptosis in laryngeal cancer cells through caspase-and mitochondria-dependent pathways. Bioochem Biophys Res Commun 298:805-814.

Watanabe T, Shinohara N, Sazawa A, Takimoto M, Hashimoto A, Koyanagi T and Kuzumaki N (2001) Adenovirus-mediated gene therapy for bladder cancer in an orthotopic model usind a dominant negative H-Ras mutant. Int J Cancer 92:712-717.

Wu H, Wang K, Liu W and Hao Q (2015) Recombinant adenovirus-mediated overexpression of PTEN and KRT10 improves cisplatin resistance of ovarian cancer In Vitro and In Vivo. Genet Mol Res 14:6591–6597.

Wu ZX, Song TB, Li DM, Zhang XT and Wu XL (2006b) Overexpression of PTEN suppresses growth and induces apoptosis by inhibiting the expression of survivin in bladder cancer cells. Tumor Biol 28:9–15.

Xie XK, Yang DS, Ye ZM and Tao HM (2009) Enhancement effect of adenovirus-mediated antisense c-myc and caffeine on the cytotoxicity of cisplatin in osteosarcoma cell lines. Chemotherapy 55:433–440.

Yang JL, Pan XY, Zhao WX, Hu QC, Ding F, Feng Q, Li GY and Luo Y (2016a) The antitumor efficacy of a novel adenovirus-mediated anti-p21Ras single chain fragment variable antibody on human cancers in vitro and in vivo. Int J Oncol 48:1218–1228.

Zhang Y-A, Nemunaitis J, Scanlon KJ and Tong AW (2000) Anti-tumorigenic effect of a K-ras ribozyme against human lung cancer cell line heterotransplants in nude mice. Gene Ther 7:2041-2050.

Zhang Z, Jiang G, Yang F and Wang J (2006) Knockdown of mutant K-ras expression by adenovirus-mediated siRNA inhibits the in vitro and in vivo growth of lung cancer cells. Cancer Biol Ther 5:1481–1486.

Zheng JN, Pei DS, Mao LJ, Liu XY, Mei DD, Zhang BF, Shi Z, Wen RM and Sun XQ (2009) Inhibition of renal cancer cell growth in vitro and in vivo with oncolytic adenovirus armed short hairpin RNA targeting Ki-67 encoding mRNA. Cancer Gene Ther 16:20–32.

Zhou YA, Zhang T, Zhao JB, Wang XP, Jiang T, Gu ZP, Wang XN and Li XF (2010) The adenovirus-mediated transfer of PTEN inhibits the growth of esophageal cancer cells in vitro and in vivo. Biosci Biotechnol Biochem 74:736–740.
