# Supplementary material for: The use of adenoviral vectors in gene therapy and vaccine approaches
Source: Genet Mol Biol. 2022 Oct 7;45(3 Suppl 1):e20220079. doi: 10.1590/1678-4685-GMB-2022-0079 (PMC9543183; doi:10.1590/1678-4685-GMB-2022-0079)
Supplement: Table S2 - [file 1415-4757-GMB-45-3-s1-e20220079-s2.pdf]

Genetics and Molecular Biology
https://doi.org/10.1590/1678-4685-GMB-2022-0079

Supplementary Material to "The use of adenoviral vectors in gene therapy and vaccine approaches"

Table S2 - Adenoviral vectors inducing growth suppressors.

Reference	Genes	Tumor type	Cell lines/models	Results
Craig et al., 1998	p16	Different types of cancer	MCF-7, MDA-MB-231, U-2 OS (all p16 mutant or null, WT Rb); Saos-2, C33a, H358, BT549 (all pRb mutant or null, p16 WT);	Higher cytotoxic effect in mutant/null p16 with WT pRb cell lines than in p16 WT pRb mutant/null cells; cell cycle arrest was observed only in WT pRb cells;
Yang et al., 2016b	p16	Laryngeal	Hep-2 (p16 null); Hep-2 tumor bearing mice;	Proliferation and invasion inhibition; decrease in tumor volume *in vivo*; downregulation of EGFR, survivin, cyclin D1;
Campbell et al., 2000	p16	Breast	MCF-7, MDA-MB-231 (all p16 null, pRb WT); BT-549 (p16 WT, mutated pRb)	Cell cycle arrest; apoptosis induction; proliferation inhibition in cell lines with mutant p16 and WT pRB;
Allay et al., 2000	p16	Prostate	PC-3, PPC-1, LNCaP, DU145 (all p16 null);	Proliferation inhibition; tumor suppression *in vivo*; the article does not evaluate the influence of pRb;
Hu et al., 2011b	p16	Liver	MHCC, HepG2, SMMC-7721, BEL-7402 (all p16 null);	Akt/survivin pathway downregulation; cell cycle arrest and anoikis;
Rhee et al., 2003	p16	Head and neck	primary tumors (JHU012 - p16 inactivation; JHU022 - WT p16; both lineages with WT pRb)	The combination between Adp16 and radiotherapy enhanced the antitumor effect;
Grim et al., 1997	p16	Bladder	EJ, UMUC-3 (both p16 null, pRb WT); J82, TCC (both p16 positive, pRb null);	Proliferation inhibition, cell cycle arrest in EJ and UMUC-3 only; induced cisplatin and paclitaxel resistance in EJ;
Chintala et al., 1997	p16	Glioma	SNB19 (null p16);	Reduced invasion, downregulation of MMP-2 expression;
Xian et al., 2007	p14(ARF) + antisense EGFR	Laryngeal	Hep-2;	Synergistic antitumor effect;
Yang et al., 2000	p14(ARF)	Mesothelioma	H28, H513, H2052, MSTO-211H; HCT116 (2 copies of WT p53);	Increase in p53 and p21 expression; pRb dephosphorylation; growth inhibition; cell cycle arrest, apoptosis induction; higher effect in HCT116;
Deng et al., 2002	p14(ARF)	Breast	MCF-7 (WT p53);	Cell cycle arrest, apoptosis induction, increase in p53, p21 and MDM2 proteins; combination with cisplatin increased cell death;
Kim et al., 2004	p14(ARF)	Osteosarcoma	U2OS (WT p53, mutant p14 ARF);	Increase in p53, p21 and MDM2 proteins; growth inhibition, apoptosis induction;

Reference	Genes	Tumor type	Cell lines/models	Results
Lu et al., 2002	p14(ARF) + p53	Lung	A549 (p53 resistant, ARF null), H1299 (p53 sensitive, ARF positive);	Adp14ARF or Adp53 alone resulted in low p53 increase, but the combination improved p53 expression and antitumor effects;
Tango et al., 2002	p14(ARF) + p53	Lung and esophageal	TE8 (WT p53); H1299, H358 (both p53 null);	Combination with Adp53 resulted in high p53 protein level also in p53 null cell lines, high cytotoxicity in comparison with p53 alone, increase of p21, p53R2 and Noxa, tumor growth inhibition *in vivo*;
Merkel et al., 2010	p19(ARF)	Melanoma and glioma	B16 (mouse), C6 (rat) (both WTp53);	In B16: p53 activation, cell cycle alteration and viability reduction *in vitro* and necrosis increase *in vivo* were only achieved with Adp19 in combination with nutlin-3. In C6: p53 activation was achieved in both treatments but better results using the combination;
Merkel et al., 2013	p19(ARF) + IFNβ	Melanoma	B16 (mouse model);	Combination increased cell death *in vitro* and higher tumor growth inhibition *in vivo*;
Catani et al., 2016	p19(ARF) + IFNβ	Lung	LLC1 mouse model;	p19ARF could induce tumor cell death alone, but the combination resulted in immunogenic cell death and protective immune response;
Roig et al., 2004	pRb hypo phosphorylated variant	Pancreatic	NP-9, NP-18, NP-31 (all pRb WT);	Expression of truncated form of pRB gene, lacking N-terminal 112 amino acid residues (RB94); high proliferation inhibition, cell cycle arrest, apoptosis induction *in vitro* and growth suppression *in vivo* in comparison to Rb WT induction;
Riley et al., 1996	pRb WT	-	Rb (+/-) immunocompetent mice with spontaneous pituitary melanotroph tumors	Decreased proliferation; growth inhibition; increased survival rate;
Fueyo et al., 1998	pRb WT	Glioma	EFC-2 (pRb -), U-251 MG (pRb +), Saos-2 (pRb -);	Loss of neoplastic morphology, growth suppression, cell cycle arrest *in vitro*; prevented tumor formation in mice only in pRb – cell lines;
Ip et al., 2001	pRb WT	Cervical	Saos-2, HeLa (HPV+, inactivated pRb), SiHa (HPV+, inactivated pRb), C-33A (HPV -);	Cell cycle arrest, reduction in colony formation in Saos-2; cell cycle arrest in SiHa and C-33A but not in HeLa; no effect in growth and colony formation in all three cervical lines;
Lin et al., 2010	SOCS3	Lung	NCI-H460; NCI-H838; NCI-H1703, NCI-A549 WI-38, 293;	Growth inhibition, cell cycle arrest; apoptosis induction; radiosensitivity enhancement;
Sugase et al., 2018	SOCS1	gastric	GIST-T1 (imatinib-sensitive), GIST-R8 (imatinib-resistant);	Proliferation inhibition and apoptosis induction; STAT3, AKT and FAK inhibition in both cell lines;
Liu et al., 2013b	SOCS1	liver	Bel-7404, Hep3B, Huh-7, SMMC7721;	STAT3 inhibition, survivin, cyclin D1, Bcl-Xl and c-MYC downregulation, and apoptosis induction.

References:

Allay JA, Steiner MS, Zhang Y, Reed CP, Cockroft J and Lu Y (2000) Adenovirus p16 gene therapy for prostate cancer. World J Urol 18: 111-20.

Campbell I, Magliocco A, Moyana T, Zheng C and Xiang J (2000) Adenovirus-mediated p16 INK4 gene transfer significantly suppresses human breast cancer growth. Cancer Gene Ther 7:1270-8.

Catani JPP, Medrano RFV, Hunger A, Valle P Del, Adjemian S, Zanatta DB, Kroemer G, Costanzi-Strauss E and Strauss BE (2016) Intratumoral immunization by p19arf and interferon-β gene transfer in a heterotopic mouse model of lung carcinoma. Transl Oncol 9:565–574.

Chintala SK, Fueyo J, Gomez-Manzano C, Venkaiah B, Bjerkvig R, Yung A, Sawaya R, Kyritsis AP and Rao JS (1997) Adenovirus-mediated p16/CDKN2 gene transfer suppresses glioma invasion in vitro. Oncogene 15:2049-2057

Craig C, Kim M, Ohri E, Wersto R, Katayose D, Li Z, Choi YH, Mudahar B, Srivastava S, Seth P *et al.* (1998) Effects of adenovirus-mediated p16 INK4A expression on cell cycle arrest are determined by endogenous p16 and Rb status in human cancer cells. Oncogene 16:265-72.

Deng X, Kim M, Vandier D, Jung Y jin, Rikiyama T, Sgagias MK, Goldsmith M and Cowan KH (2002) Recombinant adenovirus-mediated p14ARF overexpression sensitizes human breast cancer cells to cisplatin. Biochem Biophys Res Commun 296:792–798.

Fueyo J, Gomez-Manzano C, Yung WKA, Liu T-J, Alemany R, Bruner JM, Chintala SK, Rao JS, Levin VA and Kyritsis AP (1998) Suppression of human glioma growth by adenovirus-mediated Rb gene transfer. Neurology 50:1307-1315.

Grim J, Frizelle S, Zhou J, Kratzke RA, Curiel DT, R A Ki JZ and Therapy Program IJ-G (1997) Adenovirus-mediated delivery of p16 to p16-deficient human bladder cancer cells confers chemoresistance to cisplatin and paclitaxel. Clin Cancer Res 3:2415-2423

Hu H, Li Z, Chen J, Wang D, Ma J, Wang W, Li J, Wu H, Li L, Wu M *et al.* (2011b) P16 reactivation induces anoikis and exhibits antitumour potency by downregulating Akt/survivin signalling in hepatocellular carcinoma cells. Gut 60:710–721.

Ip SM, Huang TG, Yeung WSB and Ngan HYS (2001) pRb-expressing adenovirus Ad5-Rb attenuates the p53-induced apoptosis in cervical cancer cell lines. Eur J Cancer 37:2475–2483.

Kim M, Sgagias M, Deng X, Jung YJ, Rikiyama T, Lee K, Ouellette M and Cowan K (2004) Apoptosis induced by adenovirus-mediated p14ARF expression in U2OS osteosarcoma cells is associated with increased Fas expression. Biochem Biophys Res Commun 320:138–144.

Lin YC, Lin CK, Tsai YH, Weng HH, Li YC, You L, Chen JK, Jablons DM, Yang CT (2010) Adenovirus-mediated SOCS3 gene transfer inhibits the growth and enhances the radiosensitivity of human non-small cell lung cancer cells. Oncol Rep 24: 1606-1612.

Liu L, Li W, Wei X, Cui Q, Lou W, Wang G, Hu X and Qian C (2013b) Potent antitumor activity of oncolytic adenovirus-mediated SOCS1 for hepatocellular carcinoma. Gene Ther 20:84–92.

Lu W, Lin J and Chen J (2002) Expression of p14ARF overcomes tumor resistance to p53. Cancer Res 62:1305–1310.

Merkel CA, da Silva Soares RB, de Carvalho AC V, Zanatta DB, Bajgelman MC, Fratini P, Costanzi-Strauss E and Strauss BE (2010) Activation of endogenous p53 by combined p19Arf gene transfer and nutlin-3 drug treatment modalities in the murine cell lines B16 and C6. BMC Cancer 10:316.

Merkel CA, Medrano RFV, Barauna VG and Strauss BE (2013) Combined p19Arf and interferon-beta gene transfer enhances cell death of B16 melanoma in vitro and in vivo. Cancer Gene Ther 20:317–325.

Rhee JG, Li DQ, O'Malley BW and Suntharalingam M (2003) Combination radiation and adenovirus-mediated p16INK4A gene therapy in a murine model for head and neck cancer. ORL J Otohinolaryngol Relat Spec 65:144–154.

Riley DJ, Nikitin AYu and Lee WH (1996) Adenovirus-mediated retinoblastoma gene therapy suppresses spontaneous pituitary melanotroph tumors in Rb(+/-) mice. Nat Med 2:1316–1321.

Roig JM, Molina MA, Cascante A, Calbó J, Carbó N, Wirtz U, Sreedharan S, Fillat C and Mazo A (2004) Adenovirus-Mediated Retinoblastoma 94 Gene Transfer Induces Human Pancreatic Tumor Regression in a Mouse Xenograft Model. Clin Cancer Res 10:1454-1462

Sugase T, Takahashi T, Serada S, Fujimoto M, Ohkawara T, Hiramatsu K, Nishida T, Hirota S, Saito Y, Tanaka K *et al.* (2018) SOCS1 gene therapy has antitumor effects in imatinib-resistant gastrointestinal stromal tumor cells through FAK/PI3 K signaling. Gastric Cancer 21:968–976.

Tango Y, Fujiwara T, Itoshima T, Takata Y, Katsuda K, Uno F, Ohtani S, Tani T, Roth JA and Tanaka N (2002) Adenovirus-mediated p14ARF gene transfer cooperates with Ad5CMV-p53 to induce apoptosis in human cancer cells. Hum Gene Ther 13:1373–1382.

Yang CT, You L, Yeh CC, Chang JWC, Zhang F, McCormick F and Jablons DM (2000) Adenovirus-mediated p14(ARF) gene transfer in human mesothelioma cells. J Natl Cancer Inst 92:636–641.

Yang Z, Hu J, Li D and Pan X (2016b) Adenovirus with p16 gene exerts antitumor effect on laryngeal carcinoma Hep2 cells. Mol Med Rep 14:1425–1429.

Xian, J., Lin, Y., Liu, Y., Gong, P., & Liu, S. (2007). Combined p14ARF and antisense EGFR potentiate the efficacy of adenovirus-mediated gene therapy in laryngeal squamous cell carcinoma (LSCC). *DNA and cell biology*, *26*(2), 71–79. https://doi.org/10.1089/dna.2006.0533
