# Supplementary material for: The use of adenoviral vectors in gene therapy and vaccine approaches
Source: Genet Mol Biol. 2022 Oct 7;45(3 Suppl 1):e20220079. doi: 10.1590/1678-4685-GMB-2022-0079 (PMC9543183; doi:10.1590/1678-4685-GMB-2022-0079)
Supplement: Table S3 - [file 1415-4757-GMB-45-3-s1-e20220079-s3.pdf]

Genetics and Molecular Biology
https://doi.org/10.1590/1678-4685-GMB-2022-0079

Supplementary Material to "The use of adenoviral vectors in gene therapy and vaccine approaches"

Table S3 - Adenoviral vectors inducing cell death.

Reference	Genes	Tumor type	Cell lines/models	Results
Zheng et al., 2005	Fas ligand	Gastric	SGC-7901; xenograft mouse tumor model;	Cell growth and colony-formation decrease, cell cycle arrest and apoptosis induction; tumor growth suppression *in vivo*;
Sudarshan et al., 2005	Fas ligand	Bladder	T24, J82, 5637;	Induction of cell death; high cytotoxicity; death-resistance induced by monoclonal antibody anti Fas, but AdFasL overcame resistance;
ElOjeimy et al., 2006	Fas ligand	Head and neck	SCC-1, SCC-12, SCC-14a; SCC-14a xenograft mouse model;	Death-resistance induced by monoclonal antibody anti Fas, but resistance was overcome with AdFasL; Apoptosis induction, growth suppression *in vivo*;
Yamabe et al., 1999	Caspase 3	Liver	HepG2, AH130, AH130-V (overexpression of Bcl-2); AH130 liver tumor model;	Did not promote cell death alone, only in combination with etoposide had antitumor effect *in vitro* and *in vivo*; etoposide + caspase 3 did not have death effect in a Bcl-2 overexpressing tumor;
Shinoura et al., 2000	Fas Ligand and Caspase 3	Glioma	alexander hepatoma cells, MCF-7, U251, U-373MG;	Caspase-3 did not induced apoptosis; in combination with Fas ligand, high apoptosis rates were achieved in U-373MG and in U251;
Pataer et al., 2000	BAK	Lung and Breast	H1200 (null p530, H322J (mutant p53), H460 (WT p53), A549 (WT p53), MCF-7 (WT p53, caspase 3-deficient);	Apoptosis induction *in vivo* and *in vitro* in all cell lines, except MCF-7;
Pataer et al., 2001	BAK	Mesothelioma	I-45 (p53 resistant), REN (p53 sensitive);	Apoptosis induction in both cell lines, viability reduction;
Huh et al., 2001	BAX	Cervical	HeLa, C33A, CaSki;	High cytotoxicity, apoptosis induction;
Kaliberov et al., 2002	BAX	Lung	NHBE 6043NRA, BEAS-2B, A427, A549, H322, H522, H1466.	BAX under control of VEGF promoter; apoptosis and caspase induction, growth inhibition, specificity to tumor cells, hypoxia condition increased the antitumor effect;
Lowe et al., 2001	BAX	Prostate	LNCaP;	Bax under a prostate-specific promoter; viability reduction, apoptosis induction, specificity to prostate cells;
Honda et al., 2002	BAX	Prostate	DU145 (p53 mutation, Bax deficiency), PC3, LNCaP;	Apoptosis induction, caspase-3 and PARP cleavage;
Li et al., 2001b	BAX, Pro-caspase 3, Pro-caspase 7	Prostate	LNCaP, LNCaP-Bcl-2, PC-3, Du-145, TsuPr(1);	Pro-caspase 3 overexpression did not result in autocatalytic activation in all cell lines studied; pro-caspase 7 was activated in LNCaP and LNCaP-Bcl-2 only, contributing to apoptosis induction; Bax overexpression induced apoptosis in all cell lines studied; *In vivo* studies with PC-3 in mouse model treated with AdBax demonstrated almost complete tumor regression;
Tai et al., 1999	BAX	Ovarian	SKOV-3 (intermediate DF3), OVCAR-3, 36M2 (High DF3),	BAX under control of DF3 promoter; specific cytotoxicity on DF3 positive tumor cells *in vitro* and *in vivo*; Almost complete tumor regression in *in vivo* studies;

			CAOV-3 (intermediate DF3), SW626 (low DF3);	
Tsuruta et al., 2001	BAX	Ovarian	A2780 (cisplatin-sensitive), A2780/cDDP (cisplatin-resistant, p53 mutation), OVCAR-3 (cisplatin-resistant), SK-OV-3 (cisplatin-resistant);	Higher cytotoxicity in OVCAR-3, A2780/cDDP and A2780; lower cytotoxicity in SKOV3; increased cytotoxicity in combination with cisplatin or paclitaxel in all cell lines but not in A2780/cDDP;
Wack et al., 2008	BAX and TRAIL	Pancreatic	BxPC-3, Capan-1, Panc-1, Panc-1/GFP;	Under control on hTERT promoter; apoptosis induction, sensitization to gemcitabine; the combination between AdBax, AdTRAIL and gemcitabine improved tumor suppression *in vivo*;
Arafat et al., 2000	BAX	Ovarian	SKOV3.ip1, OV-4, SW626, OVCAR3, PA-1;	Sensitization to radiation treatment *in vitro* and *in vivo*;
Li et al., 2010	BAX and IL-24	Liver	HepG2, Hep3B, PLC/PRF/5;	Apoptosis induction and tumor growth suppression; synergistic effect between IL-24 and Bax;
Tazawa et al., 2013	P53	Review	-	Reviews Ad-P53 preclinical and clinical trials in several types of cancer;
Tamura et al., 2018	P53	Review	-	Reviews Ad-p53 in prostate cancer;
Tamura et al., 2016	P53	Prostate	PC3, DU145;	Ad-P53 under control of a p53-responsive promoter (AdPGp53) leading to p53 auto regulated expression; comparison with Adp53 under control of CMV promoter; PC3 were more resistant to death induced by AdPGp53 than DU145; tumor inhibition and increased overall survival only with AdPGp53 treatment;
Tamura et al., 2017	P53	Prostate	PC3, DU145;	AdPGp53 developed in Tamura et al. 2016 with incorporation of RGD motif in the adenoviral fiber protein; Increase in p53 expression, cell death, induction of oxidants, DNA damage, tumor suppression *in vivo*;
Tamura et al., 2020	P53	Prostate	PC3, DU145;	AdRGD-PGp53 in combination to different chemotherapies; AdRGD-PGp53 increase the sensibility of tumor cells to mitoxantrone, docetaxel and cabazitaxel, but cabazitaxel conferred tumor inhibition and 100% of survival *in vivo*.
Del Valle et al., 2021	P53 + IFNβ	Colon	HCT116 (wtp53), HCT116 -/- (p53 null), HT29 (mutant p53);	ADRGD-PGp53 alone was sufficient for inducing cell death in HTC116, but not in HT29, where combination with IFNβ improved the treatment.
Zhang et al., 2018a	P53	Head and neck cancer and others types of cancer	-	Safety, high efficacy in combination with radio and chemotherapy;
Wang et al., 2012	PUMA	Pancreatic	MIA, PaCa-2, PANC-1, P3, SW1990;	Apoptosis induction; sensitization to 5-fluoracil, cisplatin, gemcitabine hydrochloride; tumor growth inhibition *in vivo*;
Lisiansky et al., 2012	PUMA	Pancreatic	Colo357, Panc1, MiaPaca;	PUMA under Ras-responsive promoter; cell growth inhibition and apoptosis induction in cells with Ras activity; higher tumor growth suppression in comparison to a conventional promoter (SV40);
Giladi et al., 2007	PUMA	Prostate	PC3 (beta-catenin/Tcf pathway active); DU145 (beta-catenin-Tcf pathway inactive);	PUMA under control of beta-catenin/T-cell factor (Tcf)-responsive promoter; cell growth inhibition only in PC-3;
Wang et al., 2006	PUMA	Esophageal	KYSE-150, KYSE-410, KYSE-510, YES-2;	Higher cytotoxicity in comparison to Adp53; increased sensibility to cisplatin, paclitaxel and 5-fluoracil;

Naumov et al., 2012	PUMA	Colon	HCT116, SW480, DLD1, RIE-Ras (hyperactive Ras pathway); HT29, RIE (low Ras activity);	Under control of Ras responsive promoter; higher apoptosis rates under Ras promoter control and tumor suppression *in vivo*;
Suzuki et al., 2009	Noxa	Breast	HBC4, HBC5, MCF7, HTB26;	Apoptosis induction only in tumor cells *in vitro* and *in vivo*; Puma induced apoptosis in cancer and normal cells;
Wang et al., 2016	HSV-Tk + Ganciclovir	Bladder	T24, BIU-87, 5637, 293, LNCaP, PC12, A498, HepG2, BGC823;	High cytotoxic effect; decrease in cell survival *in vitro*, tumor growth reduction and apoptosis induction *in vivo*;
Sutton et al., 2000	HSV-Tk + Ganciclovir	Bladder	MBT-2 in vivo mouse model;	*In vivo* study; efficacy and safety confirmation;
Sutton et al., 1997	HSV-Tk + Ganciclovir	Bladder	MBT-2;	Cell death induction; survival increase *in vivo*;
Wang et al., 2009	HSV-Tk + Ganciclovir	Colon	LoVo, HT-29, HCT-8;	Under control of Cox-2 promoter; specificity to tumor cells;
Oh et al., 2010	HSV-Tk + Ganciclovir + CRAD	Lung	NCI H460, A549;	The combination improved the Ad-HSV-Tk treatment;
Song et al., 2003	HSV-Tk + Ganciclovir	Ovarian	Wi-38, QBI-293A;	Under hTERT promoter; tumoral specificity; apoptosis induction;
Ji et al., 2016	HSV-Tk + Ganciclovir	Glioma	phase II clinical trial	47 participants; increase in survival, no treatment-related severe adverse effects;
Sangro et al., 2010	HSV-Tk + Ganciclovir	Liver	phase I clinical trial	10 participants; tumor stabilization in the majority of cases; safety confirmation;
Chiocca et al., 2011	HSV-Tk + Ganciclovir	Glioma	phase Ib clinical trial	13 participants; Combination with radiotherapy and chemotherapy (temozolomide); safety confirmation;
Van Putten et al., 2010	HSV-Tk + Ganciclovir (Cerepro)	Glioma	Preclinical, rats models;	Safety characterization; Detection of the virus in the brain, and low concentration in systemic circulation and spleen;
Luo et al., 2012	HSV-Tk + Ganciclovir / CD + 5FC	Gastric	SGC7901;	Under control of survivin promoter; specificity to tumor cells; higher efficiency in combinatory treatment than in separated ones;
Freytag et al., 2007	HSV-Tk + Ganciclovir / CD + 5FC	Pancreatic	MiaPaCa-2, PANC-1;	Oncolytic adenovirus; Radiation improved the combinatory treatment; Safety demonstration;
Lee et al., 2020	HSV-Tk + Ganciclovir / CD + 5FC + gemcitabine	Pancreatic	phase I clinical trial	11 newly diagnosed locally advanced pancreatic cancer (LAPC) patients; No dose-limiting toxicity; Safety demonstration;
Barton et al., 2008	HSV-Tk + Ganciclovir / CD + 5FC	Prostate	phase I clinical trial	High safety demonstration;
Freytag et al., 2003	HSV-Tk + Ganciclovir / CD + 5FC + radiotherapy	Prostate	phase I clinical trial	15 patients; No significant adverse effect; Safety demonstration; Higher PSA reduction in combinatory treatment;

References:

Arafat WO, Gómez-Navarro J, Xiang J, Barnes MN, Mahasreshti P, Alvarez RD, Siegal GP, Badib AO, Buchsbaum D, Curiel DT *et al.* (2000) An adenovirus encoding proapoptotic Bax induces apoptosis and enhances the radiation effect in human ovarian cancer. Mol Ther 1:545–554.

Barton KN, Stricker H, Brown SL, Elshaikh M, Aref I, Lu M, Pegg J, Zhang Y, Karvelis KC, Siddiqui F *et al.* (2008) Phase I study of noninvasive imaging of adenovirus-mediated gene expression in the human prostate. Mol Ther 16:1761–1769.

Chiocca EA, Aguilar LK, Bell SD, Kaur B, Hardcastle J, Cavaliere R, McGregor J, Lo S, Ray-Chaudhuri A, Chakravarti A *et al.* (2011) Phase IB study of gene-mediated cytotoxic immunotherapy adjuvant to up-front surgery and intensive timing radiation for malignant glioma. J Clin Oncol 29:3611–3619.

Del Valle PR, Mendonça SA, Antunes F, Hunger A, Tamura RE, Zanatta DB and Strauss BE (2021) Exploration of p53 plus interferon-beta gene transfer for the sensitization of human colorectal cancer cell lines to cell death. Cancer Biol Ther 22:301–310.

ElOjeimy S, McKillop JC, El-Zawahry AM, Holman DH, Liu X, Schwartz DA, Day TA, Dong JY and Norris JS (2006) FasL gene therapy: A new therapeutic modality for head and neck cancer. Cancer Gene Ther 13:739–745.

Freytag SO, Barton KN, Brown SL, Narra V, Zhang Y, Tyson D, Nall C, Lu M, Ajlouni M, Movsas B *et al.* (2007) Replication-competent adenovirus-mediated suicide gene therapy with radiation in a preclinical model of pancreatic cancer. Mol Ther 15:1600–1606.

Freytag SO, Stricker H, Pegg J, Paielli D, Pradhan DG, Peabody J, Deperalta-Venturina M, Xia X, Brown S, Lu M *et al.* (2003) Phase I Study of Replication-Competent Adenovirus-Mediated Double-Suicide Gene Therapy in Combination with Conventional-Dose Three-Dimensional Conformal Radiation Therapy for the Treatment of Newly Diagnosed, Intermediate- to High-Risk Prostate Cancer. Cancer Res 63:7497-506

Giladi N, Dvory-Sobol H, Sagiv E, Kazanov D, Liberman E and Arber N (2007) Gene therapy approach in prostate cancer cells using an active Wnt signal. Biomed Pharmacother 61:527–530.

Honda T, Kagawa S, Spurgers KB, Gjertsen BT, Roth JA, Fang B, Lowe SL, Norris JS, Meyn RE and McDonnell TJ (2002) A recombinant adenovirus expressing wild-type bax induces apoptosis in prostate cancer cells independently of their Bcl-2 status and androgen sensitivity. Cancer Biol Ther 1:163–167.

Huh WK, Gomez-Navarro J, Arafat WO, Xiang J, Mahasreshti PJ, Alvarez RD, Barnes MN and Curiel DT (2001) Bax-induced apoptosis as a novel gene therapy approach for carcinoma of the cervix. Gynecol Oncol 83:370–377.

Ji N, Weng D, Liu C, Gu Z, Chen S, Guo Y, Fan Z, Wang X, Chen J, Zhao Y *et al.* (2016) Adenovirus-mediated delivery of herpes simplex virus thymidine kinase administration improves outcome of recurrent high-grade glioma. Oncotarget 7:4369–4378.

Kaliberov SA, Buchsbaum DJ, Gillespie GY, Curiel DT, Arafat WO, Carpenter M and Stackhouse MA (2002) Adenovirus-mediated transfer of BAX driven by the vascular endothelial growth factor promoter induces apoptosis in lung cancer cells. Mol Ther 6:190–198.

Lee J chan, Shin DW, Park H, Kim J, Youn Y, Kim JH, Kim J and Hwang JH (2020) Tolerability and safety of EUS-injected adenovirus-mediated double-suicide gene therapy with chemotherapy in locally advanced pancreatic cancer: a phase 1 trial. Gastrointest Endosc 92:1044-1052.e1.

Li J, Shi L, Zhang X, Kang X, Wen Y, Qian H, Zhou Y, Xu W, Zhang Y, Wu M *et al.* (2010) Recombinant adenovirus IL-24-Bax promotes apoptosis of hepatocellular carcinoma cells in vitro and in vivo. Cancer Gene Ther 17:771–779.

Li X, Marani M, Yu J, Nan B, Roth JA, Kagawa S, Fang B, Denner L and Marcelli M (2001b) Adenovirus-mediated Bax overexpression for the induction of therapeutic apoptosis in prostate cancer. Cancer Res 61:186–191.

Lisiansky V, Naumov I, Shapira S, Kazanov D, Starr A, Arber N and Kraus S (2012) Gene therapy of pancreatic cancer targeting the K-Ras oncogene. Cancer Gene Ther 19:862–869.

Lowe SL, Rubinchik S, Honda T, Mcdonnell TJ, Dong J-Y and Norris JS (2001) Prostate-specific expression of Bax delivered by an adenoviral vector induces apoptosis in LNCaP prostate cancer cells. Gene Ther 8:1363-1371.

Luo XR, Li JS, Niu Y and Miao L (2012) Adenovirus-mediated double suicide gene selectively kills gastric cancer cells. Asian Pac J Cancer Prev 13:781–784.

Naumov I, Kazanov D, Lisiansky V, Starr A, Aroch I, Shapira S, Kraus S and Arber N (2012) Novel approach to abuse the hyperactive K-Ras pathway for adenoviral gene therapy of colorectal cancer. Exp Cell Res 318:160–168.

Oh JY, Park MY, Kim DR, Lee JH, Shim SH, Chung JH, Yoon H Il, Lee JH, Sung MW, Kim YS *et al.* (2010) Combination gene therapy of lung cancer with conditionally replicating adenovirus and adenovirus-herpes simplex virus thymidine kinas. Int J Mol Med 25:369–376.

Pataer A, Fang B, Yu R, Kagawa S, Hunt KK, McDonnell TJ, Roth JA and Swisher SG (2000) Adenoviral Bak overexpression mediates caspase-dependent tumor killing. Cancer Res 60:788–792.

Pataer A, Smythe WR, Yu R, Fang B, McDonnell T, Roth JA and Swisher SG (2001) Adenovirus-mediated Bak gene transfer induces apoptosis in mesothelioma cell lines. J Tho Cardiovas Surg 121:61–67.

Sangro B, Mazzolini G, Ruiz M, Ruiz J, Quiroga J, Herrero I, Qian C, Benito A, Larrache J, Olagüe C *et al.* (2010) A phase i clinical trial of thymidine kinase-based gene therapy in advanced hepatocellular carcinoma. Cancer Gene Ther 17:837–843.

Shinoura N, Muramatsu Y, Yoshida Y, Asai A, Kirino T and Hamada H (2000) Adenovirus-mediated transfer of caspase-3 with Fas ligand induces drastic apoptosis in U-373MG glioma cells. Exp Cell Res 256:423–433

Song J-S, Kim H-P, Yoon W-S, Lee K-W, Kim M-H, Kim K-T, Kim H-S and Kim YT (2003) Adenovirus-mediated Suicide Gene Therapy Using the Human Telomerase Catalytic Subunit (hTERT) Gene Promoter Induced Apoptosis of Ovarian Cancer Cell Line. Biosci Biotechnol Biochem 67:2344-2350

Sudarshan S, Holman DH, Hyer ML, Voelkel-Johnson C, Dong JY and Norris JS (2005) In vitro efficacy of Fas ligand gene therapy for the treatment of bladder cancer. Cancer Gene Ther 12:12–18.

Sutton MA, Berkman SA, Chen S-H, Block A, Dang TD, Kattan MW, Wheeler TM, Rowley DR, Woo SLC and Lerner SP (1997) Adenovirus-mediated suicide gene therapy for bladder cancer. Urology 49:173–180.

Sutton MA, Freund CTM, Berkman SA, Dang TD, Kattan MW, Wheeler TM, Rowley DR and Lerner SP (2000) In vivo adenovirus-mediated suicide gene therapy of orthotopic bladder cancer. Mol Ther 2:211–217.

Suzuki S, Nakasato M, Shibue T, Koshima I and Taniguchi T (2009) Therapeutic potential of proapoptotic molecule Noxa in the selective elimination of tumor cells. Cancer Sci 100:759–769.

Tai YT, Strobel T, Kufe D and Cannistra SA (1999) In vivo cytotoxicity of ovarian cancer cells through tumor-selective expression of the BAX gene. Cancer Res 59:2121–2126.

Tamura RE, da Silva Soares RB, Costanzi-Strauss E and Strauss BE (2016) Autoregulated expression of p53 from an adenoviral vector confers superior tumor

inhibition in a model of prostate carcinoma gene therapy. Cancer Biol Ther 17:1221–1230.

Tamura RE, de Luna IV, Lana MG and Strauss BE (2018) Improving adenoviral vectors and strategies for prostate cancer gene therapy. Clinics 73:e476s

Tamura RE, Hunger A, Fernandes DC, Laurindo FR, Costanzi-Strauss E and Strauss BE (2017) Induction of oxidants distinguishes susceptibility of prostate carcinoma cell lines to p53 gene transfer mediated by an improved adenoviral vector. Hum Gene Ther 28:639–653.

Tamura RE, Lana MG, Costanzi-Strauss E and Strauss BE (2020) Combination of cabazitaxel and p53 gene therapy abolishes prostate carcinoma tumor growth. Gene Ther 27:15–26.

Tazawa H, Kagawa S and Fujiwara T (2013) Advances in adenovirus-mediated p53 cancer gene therapy. Exp Opin Biol Ther 13:1569–1583.

Tsuruta Y, Mandai M, Konishi I, Kuroda H, Kusakari T, Yura Y, Hamid AA, Tamura I, Kariya M and Fujii S (2001) Combination effect of adenovirus-mediated pro-apoptotic bax gene transfer with cisplatin or paclitaxel treatment in ovarian cancer cell lines. Eur J Cancer 37:531-541.

van Putten EHP, Dirven CMF, van den Bent MJ, Lamfers MLM (2010) Sitimagene ceradenovec: a gene=based drug for the treatment of operable high-grade glioma. Fut Oncol. 6: 1691-1710.

Wack S, Rejiba S, Parmentier C, Aprahamian M and Hajri A (2008) Telomerase transcriptional targeting of inducible bax/TRAIL gene therapy improves gemcitabine treatment of pancreatic cancer. Mol Ther 16:252–260.

Wang DG, Zhao MJ, Liu YQ, Liu XW, Niu HT, Song YF and Tian YX (2016) Fiber-modified adenovirus-mediated suicide gene therapy can efficiently eliminate bladder cancer cells in vitro and in vivo. Oncotarget 7:71710–71717.

Wang H, Pei W, Luan Q, Ma F, Zhou S, Zhao Z, Meng X, Zhang X, Liang X, Chen Y *et al.* (2012) A feasibility study on gene therapy of pancreatic carcinoma with Ad-PUMA. Cancer Biol Ther 13:712–719.

Wang H, Qian H, Yu J, Zhang X, Zhang L, Fu M, Liang X, Zhan Q and Lin C (2006) Administration of PUMA adenovirus increases the sensitivity of esophageal cancer cells to anticancer drugs. Cancer Biol Ther 5:380–385.

Wang ZX, Bian HB, Yang JS, De W and Ji XH (2009) Adenovirus-mediated suicide gene therapy under the control of Cox-2 promoter for colorectal cancer. Cancer Biol Ther.

Yamabe K, Shimizu S, Ito T, Yoshioka Y, Nomura M, Narita M, Saito I, Kanegae Y and Matsuda H (1999) Cancer gene therapy using a pro-apoptotic gene, caspase-3. Gene Ther 6:1952-1959.

Zhang WW, Li L, Li D, Liu J, Li X, Li W, Xu X, Zhang MJ, Chandler LA, Lin H *et al.* (2018a) The First Approved Gene Therapy Product for Cancer Ad-p53 (Gendicine): 12 Years in the Clinic. Hum Gene Ther 29:160–179.

Zheng S-Y, Li D-C, Zhang Z-D, Zhao J and Ge J-F (2005) Adenovirus-mediated FasL gene transfer into human gastric carcinoma. World J Gastroenterol 11:3446–3450.
