# Supplementary material for: The use of adenoviral vectors in gene therapy and vaccine approaches
Source: Genet Mol Biol. 2022 Oct 7;45(3 Suppl 1):e20220079. doi: 10.1590/1678-4685-GMB-2022-0079 (PMC9543183; doi:10.1590/1678-4685-GMB-2022-0079)
Supplement: Table S4 - [file 1415-4757-GMB-45-3-s1-e20220079-s4.pdf]

Genetics and Molecular Biology
https://doi.org/10.1590/1678-4685-GMB-2022-0079

Supplementary Material to “The use of adenoviral vectors in gene therapy and vaccine approaches"

Table S4 - Adenoviral vectors modulating tumoral angiogenesis.

Reference	Genes	Tumor type	Cell lines/models	Results
Hajitou et al., 2002	Angiostatin and Endostatin	Breast	PVDA cells; EF43.fgf-4 cells (derived from EF43);	Neovessels formation reduction *in vitro* (mouse aortic ring assay); invasion and tumors vascularization inhibition *in vivo*;
Sauter et al., 2000	Endostatin	Breast and lung	JC, Lewis lung carcinoma (LLC);	Tumor growth suppression, reduction in tumor volume *in vivo*; prevention of the pulmonary micro metastasis formation in lung carcinoma *in vivo*; decrease in tumor blood vessels;
Chen et al., 2000	Endostatin	Colon	VEGF implant angiogenesis model; human colon/liver metastasis xenograft model; Hep3B, A549;	Inhibition of endothelial cell migration *in vitro*; partial inhibition of angiogenesis in a VEGF implant angiogenesis model; increased survival *in vivo*;
Wen et al., 2001	Endostatin	Hemangioendothelioma	EOMA tumor model;	Inhibition of lung metastasis; using high intravenous dose in mice, high toxicity was achieved only in Ad-Endo, but not in Ad control;
Zhang et al., 2004	Endostatin	Gastric	SGC-7901;	Selective replicative adenovirus in telomerase-positive cells; angiogenesis inhibition, higher distortion of pre-existing vasculature in comparison to non-replicative adenovirus;
Jin et al., 2001	Endostatin	Breast	MidT2-1, MDA-MB-231;	Using endostatin-content supernatant from cells infected with Ad-Endo: tubule formation disruption, migration and proliferation inhibition, apoptosis induction in endothelial cells; endostatin containing supernatants did not have effect on MidT2-1 proliferation *in vitro*; reduction of b-FGF *in vivo*, growth inhibition of MidT2-1 tumor mouse model, little effect in MDA-MB-231 tumor mouse model;
Li et al., 2006	Endostatin	Nasopharynx	NPC CNE-2;	Proliferation and angiogenesis inhibition of vascular endothelial cells; tumor growth and angiogenesis inhibition *in vivo*;
Li et al., 2005	Endostatin	Liver	Hep3B, HepGII;	Oncolytic adenovirus expressing endostatin; tumor growth suppression in mice; better than non-replicative adenovirus expressing endostatin;
Dkhissi et al., 2003	Endostatin	Colon, breast, lung, prostate	LLC, MDA-MB-231, C51, CAL51, DU145, HT29, MC38;	Higher tumor suppression *in vivo* in C51 and HT29; endostatin-derived from fibroblasts infected with Ad-Endo inhibited C51 and HT29 growth *in vitro*; apoptosis induction in endothelial and colon cancer cells and cell cycle arrest (not seen in LLC, MDA-MB-231, MC38, DU145, CAL51);
Feldman et al., 2000	Endostatin	Colon	MC38;	Tumor growth inhibition in mice;
Fukumoto et al., 2005	Endostatin	Oral	SQUU-B;	Proliferation and tube formation inhibition in endothelial cells *in vitro*; tumor growth suppression in mice, lymph-node metastasis inhibition; downregulation of VEGF-C in tumor cells;
Pulkkanen et al., 2002	Endostatin + HSVtk/GCV	Renal	Caki-2dsd;	Synergistic effect between treatments; The majority of tumors treated with both HSVtk and Endostatin were eradicated or remained dormant;
Tanaka et al., 1998	Angiostatin	Glioma	RT2, U87MG;	Cell growth inhibition in endothelial cells; angiogenesis inhibition *in vivo*; increased apoptosis in tumor cells; tumor growth inhibition;

Reference	Genes	Tumor type	Cell lines/models	Results
Joseph et al., 2003	Angiostatin	Neuroblastoma	IGR-N835;	No effect in tumor growth and angiogenesis in comparison to the control group in xenografts tumors;
Galaup et al., 2005	Plasminogen	Breast, prostate and melanoma	MDA-MB-231, PC3, B16-F10;	Using the fragment K1-5 (full kringles) from plasminogen; decrease in endothelial cells survival, proliferation and migration *in vivo* (better than K1-3); decrease in tumor volume and vascularization (PC3 and MDA-MB-231) *in vivo*, also better than K1-3; lung metastasis inhibition in a B16F10 tumor model;
Schmitz et al., 2007	Angiostatin and full Plasminogen	Liver	-	Angiostatin and K1-5 promoted reduction in tube formation in endothelial cells *in vitro* and reduction in endothelial cell infiltration *in vivo*; tumor growth inhibition *in vivo* with similar inhibitory effects, but K1-5 improved the survival rates;
Im et al., 2001	Antisense-VEGF	Breast	MDA-MB-231; intra mammary xenografts tumors;	Reduction of VEGF expression but not cell growth *in vitro*; *in vivo*: reduction of tumor growth, VEGF expression and microvessel density;
Wu et al., 2006a	Flk-1	Ovarian	SKOV3;	Adenovirus expressing soluble Flk-1 (VEGFR-2); *in vitro*: little effect of sFlk-1 on cell growth; *in vivo*: tumor growth and angiogenesis inhibition, apoptosis induction, increased effect in combination with DDP;
Hoshida et al., 2002	Flt-1	Pancreatic	Panc-1 (low VEGF expression), PK-8 (high VEGF expression); PK-1, PK-9, PK-45H, PK-45P, AsPC-1, BxPC-3, MIA PaCa-2;	Adenovirus expressing soluble Flt-1 (VEGFR); No influence in cell growth *in vitro*; tumor suppression *in vivo* in PK-8 and Panc-1, reduction in microvessel density, increased apoptosis;
Kong et al.,1998	Flt1	Colon	tumor models: mice with pre-established splenic CT26.CL25 tumors and liver metastasis (1); mice with pre-established lung metastasis (2); mice with primary subcutaneous tumors (CT26.CL25) (3);	(1) Minimal residual splenic and liver tumors - tumor suppression; (2) metastasis reduction only with regionally administration, not systemically; (3) tumor suppression only with regionally administration;
Yoshimura et al., 2004	Flt-1	Renal	Renca murine renal cell carcinoma lung metastasis model;	Proliferation inhibition of endothelial cells; lung metastasis inhibition *in vivo*, reduced formation of neovessels, apoptosis induction;
Schmitz et al., 2005	Flt-1	Colon	CT26;	No effect on cell proliferation *in vitro*; tumor suppression *in vivo*, no effect in survival time, reduced intra tumor microvessel density;
Takayama et al., 2000	Ftl-1	Lung	NCI-H157, NCI-H460, NCI-H1299, NCI-H322, NCI-H358;	DNA synthesis inhibition in endothelial cells; no influence on H157 cell proliferation *in vitro*; in H157 *in vivo* model, resulted in tumor formation inhibition and tumor reduction, angiogenesis inhibition, increased apoptosis; this result was similar in all four cell lines studied, except H460;
Pan et al., 2004	VEGI and Endostatin	Liver	HepG2;	Endostatin-vascular endothelial growth inhibitor fusion protein; inhibited growth inhibition in endothelial cells but not in HepG2 cells and fibroblasts; neovascularization inhibition in choriallantoic membrane assay and *in vivo*; reduction in tumor size;
Kuo et al., 2001	Angiostatin, Endostatin, Flk1, Flt1 and neutropilin	Lung, fibrosarcoma, pancreatic	LLC, T241, BxPC3;	Flk1 or Flt1 transduction resulted in 80% of tumor growth inhibition in Lewis lung carcinoma, T241 fibrosarcoma and BxPC3 pancreatic carcinoma mouse models; Ad expressing angiostatin, endostatin or neutropilin were less effective.

Reference	Genes	Tumor type	Cell lines/models	Results
Saimura et al., 2002	NK4	Pancreatic	AsPC-1;	Migration inhibition *in vitro*; prevention of tumor formation *in vivo*; decreased microvessel density and apoptosis induction in tumor cells *in vivo*; improved survival;
Heideman et al., 2004	NK4	Gastric	Kato3, COS-7, MKN28, MKN45, HM02, U118MG;	Tumor cell proliferation, migration and invasion inhibition *in vitro*; endothelial cell proliferation inhibition; angiogenesis inhibition; tumor suppression *in vivo*, decrease in microvessel density;
Maemondo et al., 2002	NK4	Lung	A549, H358;	Did not affect tumor cells proliferation *in vitro*; tumor suppression *in vivo* (both cell lines *in vivo* mouse models); angiogenesis inhibition *in vivo*;
Heideman et al., 2005	NK4	Liver	HepG2, Hep3B, HuH7;	Tumor cell proliferation and invasion inhibition *in vitro*; migration decrease in non-transduced tumor cells and proliferation inhibition in endothelial cells; Tumor suppression and microvessel density decrease *in vivo*;
Compagni et al., 2000	FGFR	Pancreatic	betaHC13T;	Endothelial cell proliferation inhibition *in vitro*, angiogenesis suppression in *ex vivo*; tumor growth and angiogenesis inhibition *in vivo*; synergistic effect in combination with soluble Flt;
Guan et al., 2007	PEDF	Prostate	PC-3;	Proliferation inhibition, apoptosis induction *in vitro*; tumor formation suppression and lower microvessel density *in vivo*;
Mahtabifard et al., 2003	PEDF	Lung	CT26, LLC, KLN205, A549;	Tumor growth inhibition, higher survival, lower microvessel density *in vivo*;
Wang et al., 2003	PEDF	Liver and lung	A549, LLC, Huh-7, 293;	Angiogenesis inhibition *in vivo*; tumor growth suppression;
Merritt et al., 2004	PEDF and Flt-1	Mesothelioma	AB-12 tumor murine model;	Tumor growth inhibition, higher survival rates and better reduction in microvessel density *in vivo* using the combination in comparison to isolated treatments;
Lin et al., 1998	Soluble Tie2	Melanoma and breast	Murine mammary carcinoma 4T1 and murine melanoma B16F10.9;	Tumor growth and metastasis inhibition *in vivo*;
Popkov et al., 2005	Anti-Tie-2	Colon, Kaposi’s' sarcoma	SLK, SW1222;	Tumor growth inhibition, reduction in microvessel density *in vivo*;
Liu et al., 2003	Thrombospondin-1	Leukemia	K562;	No effect in proliferation *in vitro*; tumor growth inhibition and reduced microvessel density *in vivo*;
Hahn et al., 2004	Thrombospondin-2	Breast	TS/A, A431;	Tumor growth inhibition and reduced microvessel density;
Bouquet et al., 2006	Angiotensinogen	Breast and melanoma	MDA-MB-231, B16F10;	Endothelial Cell proliferation inhibition *in vitro*; tumor growth and vascularity suppression *in vivo*; metastasis inhibition in melanoma mouse model;
Nguyen et al., 2007	16K hPRL	Melanoma	B16F10	Tumor growth inhibition in vivo; microvessel reduction; metastasis inhibition;
Li et al. 1999	ATF	Liver	LS174T;	Metastasis suppression *in vivo*;
Tanaka et al., 1997	sPF4	Glioma		Endothelial Cells proliferation inhibition *in vitro*; tumor growth suppression and reduced vascularity *in vivo*;

References

Bouquet C, Lamandé N, Brand M, Gasc JM, Jullienne B, Faure G, Griscelli F, Opolon P, Connault E, Perricaudet M et al. (2006) Suppression of angiogenesis, tumor growth, and metastasis by adenovirus-mediated gene transfer of human angiotensinogen. Mol Ther 14:175–182.

Compagni A, Wilgenbus P, Impagnatiello M-A, Cotten M and Christofori G (2000) Fibroblast Growth Factors Are Required for Efficient Tumor Angiogenesis. Cancer Res 60:7163-7169.

Chen CT, Lin J, Li Q, Phipps SS, Jakubczak JL, Stewart DA, Skripchenko Y, Forry-Schaudies S, Wood J, Schnell C et al. (2000) Antiangiogenic Gene Therapy for Cancer via Systemic Administration of Adenoviral Vectors Expressing Secretable Endostatin. Hum Gene Ther 11:1983-96.

Dkhissi F, Lu HE, Soria C, Opolon P, Griscelli F, Liu H, Khattar P, Mishal Z, Perricaudet M and Li H (2003) Endostatin Exhibits a Direct Antitumor Effect in Addition to Its Antiangiogenic Activity in Colon Cancer Cells. Hum Gene Ther 14:997-1008.

Feldman AL, Restifo NP, Alexander HR, Bartlett DL, Hwu P, Seth P and Libutti SK (2000) Antiangiogenic Gene Therapy of Cancer Utilizing a Recombinant Adenovirus to Elevate Systemic Endostatin Levels in Mice. Cancer Res 60:1503–1506.

Fukumoto S, Morifuji M, Katakura Y, Ohishi M and Nakamura S (2005) Endostatin inhibits lymph node metastasis by a down-regulation of the vascular endothelial growth factor C expression in tumor cells. Clin Exp Metastasis 22:31–38.

Galaup A, Magnon C, Rouffiac V, Opolon P, Opolon D, Lassau N, Tursz T, Perricaudet M and Griscelli F (2005) Full kringles of plasminogen (aa 1-566) mediate complete regression of human MDA-MB-231 breast tumor xenograted in nude mice. Gene Ther 12:831–842.

Guan M, Jiang H, Xu C, Xu R, Chen Z and Lu Y (2007) Adenovirus-mediated PEDF expression inhibits prostate cancer cell growth and results in augmented expression of PAI-2. Cancer Biol Ther 6:419–425.

Hahn W, Ho SH, Jeong JG, Hahn EY, Kim S, Yu SS, Kim S, Kim JM (2004) Viral vector-mediated transduction of a modified thrombospondin-2 cDNA inhibits tumor growth and angiogenesis.GeneTher. 2004 May;11(9):739-45

Hajitou A, Grignet C, Devy L, Berndt S, Blacher S, Deroanne CF, Bajou K, Fong T, Chiang Y, Foidart JM et al. (2002) The antitumoral effect of endostatin and angiostatin is associated with a down-regulation of vascular endothelial growth factor expression in tumor cells. FASEB J 16:1802–1804.

Heideman DAM, Overmeer RM, Van Beusechem VW, Lamers WH, Hakvoort TBM, Snijders PJF, Craanen ME, Offerhaus GJA, Meijer CJLM and Gerritsen WR (2005) Inhibition of angiogenesis and HGF-cMET-elicited malignant processes in human hepatocellular carcinoma cells using adenoviral vector-mediated NK4 gene therapy. Cancer Gene Ther 12:954–962.

Heideman DAM, Van Beusechem VW, Bloemena E, Snijders PJF, Craanen ME, Offerhaus GJA, Derksen PWB, De Bruin M, Witlox M A, Molenaar B, Meijer CJLM and Gerritsen WR (2004) Suppression of tumor growth, invasion and angiogenesis of human gastric cancer by adenovirus-mediated expression of NK4. J Gene Med 3:317-27.

Hoshida T, Sunamura M, Duda DG, Egawa S, Miyazaki S, Shineha R, Hamada H, Ohtani H, Satomi S and Matsuno S (2002) Gene Therapy for Pancreatic Cancer Using

an Adenovirus Vector Encoding Soluble flt-1 Vascular Endothelial Growth Factor Receptor. Pancreas 25:111-121.

Im SA, Kim JS, Gomez-Manzano C, Fueyo J, Liu TJ, Cho MS, Seong CM, Lee SN, Hong YK and Yung WKA (2001) Inhibition of breast cancer growth in vivo by antiangiogenesis gene therapy with adenovirus-mediated antisense-VEGF. Br J Cancer 84:1252–1257.

Jin X, Bookstein R, Wills K, Avanzini J, Tsai V, LaFace D, Terracina G, Shi B and Nielsen LL (2001) Evaluation of endostatin antiangiogenesis gene therapy in vitro and in vivo. Cancer Gene Ther 8:982–989.

Joseph JM, Bouquet C, Opolon P, Morizet J, Aubert G, Rössler J, Gross N, Griscelli F, Perricaudet M and Vassal G (2003) High level of stabilized angiostatin mediated by adenovirus delivery does not impair the growth of human neuroblastoma xenografts. Cancer Gene Ther 10:859–866 .

Kong HL, Hecht D, Song W, Kovesdi I, Hackett NR, Yayon A and Crystal RG (1998) Regional suppression of tumor growth by in vivo transfer of a cDNA encoding a secreted form of the extracellular domain of the flt-1 vascular endothelial growth factor receptor. Hum Gene Ther 9:823–833.

Kuo CJ, Farnebo F, Yu EY, Christofferson R, Swearingen RA, Carter R, Von Recum HA, Yuan J, Kamihara J, Flynn E et al. (2001) Comparative evaluation of the antitumor activity of antiangiogenic proteins delivered by gene transfer. Proc Natl Acad Sci U S A 98:4605–4610

Li H, Griscelli F, Lindenmeyer F, Opolon P, Sun L-Q, Connault E, Soria J, Soria C, Perricaudet M, Yeh P et al. (1999) Systemic Delivery of Antiangiogenic Adenovirus AdmATF Induces Liver Resistance to Metastasis and Prolongs Survival of Mice. Hum Gene Ther 10:3045-53

Li G, Sham J, Yang J, Su C, Xue H, Chua D, Sun L, Zhang Q, Cui Z, Wu M et al. (2005) Potent antitumor efficacy of an E1B 55kDa-deficient adenovirus carrying murine endostatin in hepatocellular carcinoma. Int J Cancer 113:640–648.

Li L, Liu RY, Huang JL, Liu QC, Li Y, Wu PH, Zeng YX and Huang W (2006) Adenovirus-mediated intra-tumoral delivery of the human endostatin gene inhibits tumor growth in nasopharyngeal carcinoma. Int J Cancer 118:2064–2071.

Lin P, Buxton JA, Acheson A, Radziejewski C, Maisonpierre PC, Yancopoulos GD, Channon KM, Hale LP, Dewhirst MW, George SE et al. (1998) Antiangiogenic gene therapy targeting the endothelium-specific receptor tyrosine kinase Tie2. Proc Natl Acad Sci U S A 95:8829-8834

Liu P, Wang Y, Li YH, Yang C, Zhou YL, Li B, Lu SH, Yang RC, Cai YL, Tobelem G et al. (2003) Adenovirus-mediated gene therapy with an antiangiogenic fragment of thrombospondin-1 inhibits human leukemia xenograft growth in nude mice. Leuk Res 27:701–708.

Maemondo M, Narumi K, Saijo Y, Usui K, Tahara M, Tazawa R, Hagiwara K, Matsumoto K, Nakamura T and Nukiwa T (2002) Targeting angiogenesis and HGF

function using an adenoviral vector expressing the HGF antagonist NK4 for cancer therapy. Mol Ther 5:177–185.

Mahtabifard A, Merritt RE, Yamada RE, Crystal RG and Korst RJ (2003) In vivo gene transfer of pigment epithelium-derived factor inhibits tumor growth in syngeneic murine models of thoracic malignancies. J Tho Cardiovas Surg 126:28–38.

Merritt RE, Yamada RE, Wasif N, Crystal RG and Korst RJ (2004) Effect of inhibition of multiple steps of angiogenesis in syngeneic murine pleural mesothelioma. Ann Thorac Surg 78:1042–1051.

Nguyen NQN, Cornet A, Blacher S, Tabruyn SP, Foidart JM, Noël A, Martial JA and Struman I (2007) Inhibition of tumor growth and metastasis establishment by adenovirus-mediated gene transfer delivery of the antiangiogenic factor 16K hPRL. Mol Ther 15:2094–2100.

Pan X, Wang Y, Zhang M, Pan W, Qi ZT and Cao GW (2004) Effects of endostatin-vascular endothelial growth inhibitor chimeric recombinant adenoviruses on antiangiogenesis. World J Gastroenterol 10:1409–1414.

Popkov M, Jendreyko N, Mcgavern DB, Rader C and Barbas Iii CF (2005) Targeting Tumor Angiogenesis with Adenovirus-Delivered Anti-Tie-2 Intrabody. Cancer Res 65:972-81.

Pulkkanen KJ, Laukkanen JM, Fuxe J, Rehn M, Kannatso JM, Parkkinen JJ, Kauppinen RA, Pettersson RF and Yla-Herttuala S (2002) The combination of HSV-tk and endostatin gene therapy eradicates orthotopic human renal cell carcinomas in nude mice. Cancer Gene Ther 9:908–916.

Saimura M, Nagai E, Mizumoto K, Maehara N, Okino H, Katano M, Matsumoto K, Nakamura T, Narumi K, Nukiwa T et al. (2002) Intraperitoneal injection of adenovirus-mediated NK4 gene suppresses peritoneal dissemination of pancreatic cancer cell line AsPC-1 in nude mice. Cancer Gene Ther 9:799–806.

Sauter B V, Martinet O, Zhang W-J, Mandeli J, Woo SLC and Folkman MJ (2000) Adenovirus-mediated gene transfer of endostatin in vivo results in high level of transgene expression and inhibition of tumor growth and metastases. Proc Natl Acad Sci U S A 97:4802-4807.

Schmitz V, Kornek M, Hilbert T, Dzienisowicz C, Raskopf E, Rabe C, Sauerbruch T, Qian C and Caselmann WH (2005) Treatment of metastatic colorectal carcinomas by systemic inhibition of vascular endothelial growth factor signaling in mice. World J Gastroenterol 11:4332–4336.

Schmitz V, Raskopf E, Gonzalez-Carmona MA, Vogt A, Rabe C, Leifeld L, Kornek M, Sauerbruch T and Caselmann WH (2007) Plasminogen fragment K1-5 improves survival in a murine hepatocellular carcinoma model. Gut 56:271–278

Tanaka T, Manome Y, Wen P, Kufe DW and Fine HA (1997) Viral vector-mediated transduction of a modified platelet factor 4 cDNA inhibits angiogenesis and tumor growth. Nat Med 3:437–442.

Tanaka T, Cao Y, Folkman J and Fine1 HA (1998) Viral Vector-targeted Antiangiogenic Gene Therapy Utilizing an Angiostatin Complementary DNA. Cancer Res 58:3362-9.

Takayama K, Ueno H, Nakanishi Y, Sakamoto T, Inoue K, Shimizu K, Oohashi H and Hara N (2000) Suppression of Tumor Angiogenesis and Growth by Gene Transfer of a Soluble Form of Vascular Endothelial Growth Factor Receptor into a Remote Organ. Cancer Res 60:2169-2177.Wang L, Schmitz V, Perez-Mediavilla A, Izal I, Prieto J and Qian C (2003) Suppression of angiogenesis and tumor growth by adenoviral-mediated gene transfer of pigment epithelium-derived factor. Mol Ther 8:72–79.

Wen X-Y, Bai Y and Stewart AK (2001) Adenovirus-Mediated Human Endostatin Gene Delivery Demonstrates Strain-Specific Antitumor Activity and Acute Dose-Dependent Toxicity in Mice. Human Gene Ther 12:347-358 .

Wu Y, Li Z-Y, Zhao X, Kan B and Wei Y-Q (2006a) Inhibition of Ovarian Tumor Growth by Gene Therapy with Recombinant Soluble Vascular Endothelial Growth Factor Receptor 2. Hum Gene Ther 17:941-948.

Yoshimura I, Mizuguchi Y, Miyajima A, Asano T, Tadakuma T and Hayakawa M (2004) Suppression of lung metastasis of renal cell carcinoma by the intramuscular gene transfer of a soluble form of vascular endothelial growth factor receptor I. J Urol 171:2467–2470.

Zhang Q, Nie M, Sham J, Su C, Xue H, Chua D, Wang W, Cui Z, Liu Y, Liu C et al. (2004) Effective Gene-Viral Therapy for Telomerase-Positive Cancers by Selective Replicative-Competent Adenovirus Combining with Endostatin Gene. Cancer Res 64:5390-5397.
