# Supplementary material for: The use of adenoviral vectors in gene therapy and vaccine approaches
Source: Genet Mol Biol. 2022 Oct 7;45(3 Suppl 1):e20220079. doi: 10.1590/1678-4685-GMB-2022-0079 (PMC9543183; doi:10.1590/1678-4685-GMB-2022-0079)
Supplement: Table S5 - [file 1415-4757-GMB-45-3-s1-e20220079-s5.pdf]

Genetics and Molecular Biology
https://doi.org/10.1590/1678-4685-GMB-2022-0079

Supplementary Material to "The use of adenoviral vectors in gene therapy and vaccine approaches"

Table S5 - Adenoviral vectors modulating invasion and metastasis.

Reference	Genes	Tumor type	Cell lines/models	Results
Liu et al., 2015	Cx43	Gastric	BGC-823;	Decrease in colony formation capacity, invasion and proliferation; increase in cell apoptosis and chemotherapy sensitivity;
Lee et al., 2017	shRNA CD44	Colon	HCT116;	Decrease in proliferation, migration, invasion, Akt and GSKβ phosphorylation, Bcl-2 and Bcl-XL expression; increase in apoptosis, Bax, cleaved caspase-3 and -9, and PARP expression;
Chetty et al., 2006	siRNA MMP-2	Lung	A549;	MMP-2 downregulation; migration and invasion inhibition *in vitro*; tumor growth formation inhibition *in vivo*;
Tsung et al., 2008	siRNA MMP-2	Melanoma	A2058; nude mouse spinal metastatic model;	Cell migration and invasion inhibition; suppression of vascular formation; complete retention of neurological function after treatment *in vivo*; tumor growth inhibition *in vivo*;
Ala-Aho et al., 2004	anti-MMP-13 ribozyme	Head and neck	UT-SCC-7;	Invasion inhibition *in vitro*, apoptosis induction, tumor growth suppression *in vivo*;
Li et al., 2001a	TIMP-2	Lung, colon and breast	MDA-MB-231, LLC, C51;	Invasion inhibition in tumor and endothelial cell lines, not affecting cell proliferation; inhibition of tumor formation *in vivo*; tumor growth and angiogenesis suppression;
Brand et al., 2000	TIMP-2	Liver	LS174T; colorectal liver metastasis mouse model;	Metastasis and tumor growth reduction *in vivo*;
Kim et al., 2006	TIMP-2	Breast, prostate, ovarian	MDA-MB-231, LNCaP, SKOV3.ip1, 293; MDA-MB-231 xenograft model;	Oncolytic adenovirus; tumor growth suppression and angiogenesis inhibition *in vivo*;
Miyagi et al., 2007	TIMP-1	Gastric	MKN-45P; peritoneal metastasis model in mice;	Invasion capacity inhibition *in vitro*; reduction in number and weight of peritoneal nodes *in vivo*; reduction in tumor vessels *in vivo*;
Baker et al., 1999	TIMP-3	Cervical, fibrosarcoma	HeLa, HT1080;	Invasion inhibition, cell cycle modulation and apoptosis induction *in vitro*;
Lin et al., 2012	TIMP-3	Colon	CT26;	Tumor cell growth inhibition and apoptosis induction; Adhesion, migration and invasion reduction *in vitro*; tumor growth inhibition and liver metastasis reduction *in vivo*;
Kopitz et al., 2005	Cystatin C	Fibrosarcoma	HT1080; lung and liver metastasis mouse model;	Reduction in lung metastasis formation;
Lakka et al., 2001	uPAR antisense	Lung	H1299 (high uPAR expression), A549;	Decrease in uPAR expression; reduction in Matrigel invasion capacity in both cell lines; decrease in lung metastasis *in vivo*;
Rao et al., 2005	uPAR antisense + MMP9 antisense	Lung	H1299;	Decrease in invasion capacity and capillary-like structure formation in co-culture with endothelial cells *in vitro*; tumor suppression and metastasis inhibition *in vivo*;
Lakka et al., 2003	antisense uPAR + antisense MMP-9	Glioblastoma	SNB19, U87MG;	Reduction in invasion capacity *in vitro* and in *ex vivo* assay; tumor regression in U87 tumors *in vivo*;

Reference	Genes	Tumor type	Cell lines/models	Results
Xiao et al., 2020	CD55-Smad4	Colon	HCT116, HT-29, SW620, SW480;	Oncolytic adenovirus; cell proliferation inhibition *in vitro* and *in vivo*; caspase activation, apoptosis induction; migration and invasion suppression *in vitro*; inhibition of colony and spheroids formation capacity;
Duda et al., 2003	Smad4	Pancreatic	Panc-1, MiaPaCa2 (SMAD4 WT); PK-1, BxPC3, PCI-35 (SMAD4 deleted);	Did not affected proliferation *in vitro* (all cell lines); tumor growth inhibition *in vivo*, reduction in invasion and angiogenesis, negative regulation of ETS-1 (only tested in SMAD4 deleted cell lines);

References

Ala-Aho R, Ahonen M, George SJ, Heikkilä J, Grénman R, Kallajoki M and Kähäri VM (2004) Targeted inhibition of human collagenase-3 (MMP-13) expression inhibits squamous cell carcinoma growth in vivo. Oncogene 23:5111–5123.

Baker AH, George SJ, Zaltsman AB, Murphy G and Newby AC (1999) Inhibition of invasion and induction of apoptotic cell death of cancer cell lines by overexpression of TIMP-3. Br J Cancer 79:1347–1355.

Brand K, Baker AH, Perez-Cantó A, Possling A, Sacharjat M, Geheeb M and Arnold W (2000) Treatment of Colorectal Liver Metastases by Adenoviral Transfer of Tissue Inhibitor of Metalloproteinases-2 into the Liver Tissue. Cancer Res 60:5723-5730.

Chetty C, Bhoopathi P, Joseph P, Chittivelu S, Rao JS and Lakka S (2006) Adenovirus-mediated small interfering RNA against matrix metalloproteinase-2 suppresses tumor growth and lung metastasis in mice. Mol Cancer Ther 5:2289–2299.

Duda DG, Sunamura M, Lefter LP, Furukawa T, Yokoyama T, Yatsuoka T, Abe T, Inoue H, Motoi F, Egawa SI *et al.* (2003) Restoration of SMAD4 by gene therapy reverses the invasive phenotype in pancreatic adenocarcinoma cells. Oncogene 22:6857–6864.

Kim MH, Bodenstine TM, Sumerel LA, Rivera AA, Baker AH and Douglas JT (2006) Tissue inhibitor of metalloproteinases-2 improves antitumor efficacy of a replicating adenovirus in vivo. Cancer Biol Ther 5:1647–1653.

Kopitz C, Anton M, Gansbacher B and Krüger A (2005) Reduction of experimental human fibrosarcoma lung metastasis in mice by adenovirus-mediated cystatin C overexpression in the host. Cancer Res 65:8608–8612.

Lakka SS, Gondi CS, Yanamandra N, Dinh DH, Olivero WC, Gujrati M, Rao JS, H D ND and G PM (2003) Synergistic Down-Regulation of Urokinase Plasminogen

Activator Receptor and Matrix Metalloproteinase-9 in SNB19 Glioblastoma Cells Efficiently Inhibits Glioma Cell Invasion, Angiogenesis, and Tumor Growth 1. Cancer Res 63:2454-61

Lakka SS, Rajagopal R, Rajan MK, Mohan PM, Adachi Y, Dinh DH, Olivero WC, Gujrati M, Ali-Osman F, Roth JA *et al.* (2001) Adenovirus-mediated Antisense Urokinase-Type Plasminogen Activator Receptor Gene Transfer Reduces Tumor Cell Invasion and Metastasis in Non-Small Cell Lung Cancer Cell Lines. Clin Cancer Res 7:1087-1093.

Lee SY, Kim KA, Kim CH, Kim YJ, Lee JH and Kim HR (2017) CD44-shRNA recombinant adenovirus inhibits cell proliferation, invasion, and migration, and promotes apoptosis in HCT116 colon cancer cells. Int J Oncol 50:329–336.

Li H, Lindenmeyer F, Grenet C, Opolon P, Menashi S, Soria C, Yeh P, Perricaudet M and Lu H (2001a) AdTIMP-2 inhibits tumor growth, angiogenesis, and metastasis, and prolongs survival in mice. Hum Gene Ther 12:515–526.

Lin H, Zhang Y, Wang H, Xu D, Meng X, Shao Y, Lin C, Ye Y, Qian H and Wang S (2012) Tissue inhibitor of metalloproteinases-3 transfer suppresses malignant behaviors of colorectal cancer cells. Cancer Gene Ther 19:845–851.

Liu D, Zhou H, Wu J, Liu W, Li Y, Shi G, Yue X, Sun X, Zhao Y, Hu X *et al.* (2015) Infection by Cx43 adenovirus increased chemotherapy sensitivity in human gastric cancer BGC-823 cells: Not involving in induction of cell apoptosis. Gene 574:217–224.

Miyagi M, Aoyagi K, Kato S and Shirouzu K (2007) The TIMP-1 gene transferred through adenovirus mediation shows a suppressive effect on peritoneal metastases from gastric cancer. Int J Clin Oncol 12:17–24.

Tsung AJ, Kargiotis O, Chetty C, Lakka SS, Gujrati M, Spomar DG, Dinh DH and Rao JS (2008) Downregulation of matrix metalloproteinase-2 (MMP-2) utilizing adenovirus-mediated transfer of small interfering RNA (siRNA) in a novel spinal metastatic melanoma model. Int J Oncol 32:557–564.

Xiao B, Zhang L, Liu H, Fang H, Wang C, Huang B, Liu X, Zhou X and Wang Y (2020) Oncolytic adenovirus cd55-smad4 suppresses cell proliferation, metastasis, and tumor stemness in colorectal cancer by regulating wnt/β-catenin signaling pathway. Biomedicines 8:593.
