# Supplementary material for: The use of adenoviral vectors in gene therapy and vaccine approaches
Source: Genet Mol Biol. 2022 Oct 7;45(3 Suppl 1):e20220079. doi: 10.1590/1678-4685-GMB-2022-0079 (PMC9543183; doi:10.1590/1678-4685-GMB-2022-0079)
Supplement: Table S6 - [file 1415-4757-GMB-45-3-s1-e20220079-s6.pdf]

Genetics and Molecular Biology
https://doi.org/10.1590/1678-4685-GMB-2022-0079

# Supplementary Material to "The use of adenoviral vectors in gene therapy and vaccine approaches"

**Table S6** - Adenoviral vectors modulating immune system.

| Reference | Genes | Tumor type | Cell lines/models | Results |
|---|---|---|---|---|
| Jiang et al., 2015b | TNFα | Lung and esophageal | A549, TE-1; TE-1 xenografts mouse model; | Oncolytic adenovirus; tumor cell proliferation reduction and apoptosis induction; tumor growth suppression in TE-1 *in vivo*; |
| Furukawa et al., 2011 | TNFα and NfκB inhibitor | Pancreatic | MIAPaCa-2; | Ad-TNFα in combination with nafamostat mesilate; improve in TNF apoptosis induction with NF-κB inhibitor; Higher inhibitory effect in tumor growth *in vivo* with combinatory treatment; |
| Li et al., 2014 | TRAIL | Esophageal | Eca-109; | Adenovirus transduced mesenchymal stem cells action on Eca-109 tumor cells; proliferation inhibition and apoptosis induction *in vitro*; tumor growth inhibition in xenograft mouse model; |
| Yang et al., 2015 | TRAIL | Lung | A549; | Oncolytic adenovirus; cell growth inhibition, apoptosis induction, high cytotoxicity; tumor growth inhibition *in vivo* and improved survival; |
| Zhou et al., 2017 | TRAIL | Gastric | MKN45, HGC27, SGC-7901, MKN28; xenograft peritoneal carcinomatosis mouse model; | Oncolytic adenovirus; apoptosis induction only in tumor cells, not in normal cells; metastasis inhibition and improved survival *in vivo*; |
| Shim et al., 2010 | TRAIL | Head and neck | SNU-1041, SNU-1066, SNU-1076; | Oncolytic adenovirus; Tumor cell proliferation suppression, cell cycle arrest, apoptosis induction; complete regression of tumors *in vivo* only with CRAd-TRAIL (not with AdTRAIL alone or empty oncolytic adenovirus); |
| Cao et al., 2011b | TRAIL | Liver | HepG2, Hep3B, BEL7404, BEL-7404, SNNC-7721, Bcap-37, SW620; | Oncolytic adenovirus; autophagy and apoptosis induction; simian virus 40 enhancer/alpha-fetoprotein composite promoter (AFPep); |
| Mao et al., 2014 | TRAIL | Bladder | T24; | Oncolytic adenovirus; enhanced gemcitabine effects, apoptosis induction *in vivo* and *in vitro*; |
| Ohashi et al., 2005 | IFNα | Pancreatic | Panc-1, AsPC01, BxPC-3, MIAPaCa-2; | Using human IFNα in a mouse model of human pancreatic tumor: tumor growth suppression *in vivo*, high IFNα concentration in tumor site, low quantity in blood circulation; Using mouse IFNα: tumor inhibition in all mice, natural killer cells stimulation, untreated tumors in distant sites were also affected by the treatment; |
| Hara et al., 2007 | IFNα | Pancreatic | PGHAM-1 (hamster pancreatic cells); syngeneic pancreatic cancer model in hamster; | Adenovirus expressing hamster IFNα: tumor growth suppression in injected and in non-treated distant tumors; T-cells and natural killer cells recruitment to tumor site; |
| Larocca et al., 2015 | IFNα | Pancreatic | Hap-T1, HP-1, Taka-1, PC-1, PGHam-1 (all hamster pancreatic tumor cell lines); MIA PaCa-2 (human); hamster | Oncolytic adenovirus under control of Cox2 promoter; Tumor growth suppression and increases survival *in vivo*; |

| Reference | Genes | Tumor type | Cell lines/models | Results |
|---|---|---|---|---|
| | | | pancreatic tumors *in vivo*; | |
| Cao et al., 2001 | IFNβ | Prostate | PC3MM2; orthotopic tumors in mice; | Tumor suppression *in vivo*, metastasis inhibition, microvessel reduction and proliferating cells, apoptosis induction; |
| He et al., 2008 | IFNβ | Liver | HepG2, BEL7404; | Oncolytic adenovirus; higher cytotoxic effect in comparison to empty oncolytic adenovirus and non-replicative adenovirus expressing IFNβ; |
| Park et al., 2010 | IFNβ | Lung | LLC, A549; | Oncolytic adenovirus in combination to Ad expressing IFNβ; Higher IFNβ secretion with combined treatment in comparison to Ad-IFNβ alone; media transfer from infected to non-infected cells resulted in IFNβ production; decreased tumorigenicity *in vivo*; Adding irradiation, the survival was increased; |
| Xie et al., 2013 | IFNγ | Pancreatic | Capan-2; | Tumor cell growth inhibition, apoptosis induction *in vitro*; tumor suppression *in vivo*; low systemic toxicity; |
| Zhao et al., 2007 | IFNγ | Prostate | DU-145; | Proliferation inhibition *in vitro*; tumor growth inhibition *in vivo*, low toxicity; |
| Chang et al., 2011 | IL-24 | Colon | HT-29, HT-29/oxa; | Growth inhibition and apoptosis induction *in vitro*; tumor suppression *in vivo*; induction of VEGF, GADD and MVD *in vivo*; |
| Liu et al.,2013a | IL-24 | Nasopharynx | CNE-2Z; | In combination with radiotherapy: enhanced growth inhibition, cell cycle arrest and apoptosis *in vitro* and *in vivo* mouse model; enhanced up regulation of p21 and p27 CDK inhibitors, Bax and Bcl-2, cleaved caspase-3 and downregulation of cyclin E and CDK2 *in vivo* and *in vitro*; enhanced tumor vessels reduction *in vivo*; |
| Xu et al., 2014 | IL-24 and Oncostatin M (OSM) | Melanoma | A375; | Growth suppression and apoptosis induction *in vitro* and *in vivo*; downregulation of CDK4 and cyclin D1 *in vitro* and CD34 *in vivo*; |
| Cao et al., 2011a | IL24 and SOCS3 | Liver | HepG2, Hep3B; | Oncolytic adenoviruses expressing IL-24 and SOC3; higher antitumor effect in comparison to empty oncolytic adenovirus, and isolated treatments *in vitro* and *in vivo*; |
| Zhao et al., 2013 | IL24 and inhibitor of growth family member (ING4) | Breast | MDA-MB-231; | Adenovirus expressing both genes; growth suppression, apoptosis induction, cell cycle arrest *in vitro*; combination with radiotherapy enhanced tumor suppression *in vivo*; |
| Tong et al., 2005 | IL-24 | Advanced cancer patients | Phase I Clinical Trials | 22 patients; Apoptosis induction in tumor; Increase in serum IL-6, IL-10 and TNF-α; |
| Yang et al., 2012 | IL12 + CIK cells | Liver | Hep3B, BEL7404, SMMC7721, MHCC97H, HuH-7; | Oncolytic adenovirus; Higher IL-12 expression induction comparing to non-replicative adenovirus; Specific cytotoxicity to liver cancer cells; Combination with CIK cells improved treatment *in vitro* and *in vivo*; |
| Bortolanza et al., 2009 | IL12 | Pancreatic | A549, HuH-7, BxPC-3, PANC-1, AsPC-1, RCC10, VHL53, H2T; hamster and mouse pancreatic xenografts model; | Oncolytic adenovirus; increased effect in comparison to CRAd carrying luciferase and less toxicity comparing to non-replicative adenovirus expressing IL-12; enhancement of leucocytes proliferation, indicating that an immune response was activated; |
| Freytag et al., 2013 | IL12 + CD + TK | Prostate | DU145, TRAMP-C2, B16-F10, YAC-1; prostate adenocarcinoma murine model. | Oncolytic adenovirus; IL12 + two suicide gene therapies improved metastasis inhibition and survival; Increased IL-12 and IFNγ in serum and tumor; Increased natural killer cells and cytotoxic T cells activity; antitumor immunity tumor-specific; |
| Barton et al., 2021 | IL12 + CD + TK | Pancreatic | 12 patients with metastatic pancreatic cancer; | Oncolytic adenovirus; phase I clinical trial; no serious side effect; IL12, IFNγ and CXCL10 detected in blood serum; immune activation; |
| Jiang et al., 2017 | IL12 + TGFβ inhibitor | Melanoma | B16 xenograft mouse model; | Tumor growth suppression and increased survival *in vivo*, CD4 and CD8 T cells activation, NK cells and IFNγ secretion in the tumor site; |
| Sangro et al., 2004 | IL12 | Advanced digestive tumors | Phase I clinical trial | 21 patients; 44 injections of Ad-12; Dose-limiting toxicity was not achieved, being well tolerated; Transient side |

| Reference | Genes | Tumor type | Cell lines/models | Results |
|---|---|---|---|---|
| | | | | effects (fever, malaise, sweeting and lymphopenia); Immune cells infiltration in tumors; 29% of the patients presented disease stability and one had partial remission of the tumor; |
| Chaurasiya et al., 2016 | IL2 | Breast | MT1A2, MTHJ, MDA-MB-468, T47D; | Under control of mamma globin promoter/enhancer (MPE2);Higher expression in tumor breast cells than in normal cells; specificity was similar with hTERT promoter; MPE2 promoter generated low or no toxicity; tumor growth suppression; |
| Stewart et al., 1999 | IL2 | Metastatic Breast and melanoma | Phase I Clinical Trial | Cohort of 23 patients; Minor side effects (inflammation in the site of infection); No systemic IL-2 was detected; Confirms safety; tumor regression in 24% of the patients; tumor lymphocytic infiltration, |
| Dummer et al., 2008 | IL2 | Melanoma and solid tumors | Phase I-II Clinical Trial | 35 patients; Common side effects were flu-like symptoms and reactions in the site of injection; Lymphocytic infiltration promotion in tumor; |
| Trudel et al., 2003 | IL2 | Prostate | Phase I Clinical Trial | 12 patients; Adverse effects: perineal discomfort, hematuria, flu-like symptoms (2), urinary hesitancy (1); Lymphocytic infiltration in tumor, promoting increase in IFNγ and IL-4 secretion within the tumor; decrease in PSA levels; |
| Oh et al., 2004 | IL3 | Prostate | TRAMP-C1, syngeneic murine prostate cancer model; | No effect in tumor growth, only with radiation; increased radiotherapy effect; |
| Yan et al., 2019a | IL15 | Breast | MDA-MB-231; | Oncolytic adenovirus; enhanced antitumor activity in comparison to empty oncolytic adenovirus; |
| Iida et al., 2010 | CD40L | Colon/liver | RCN9; | Tumor growth suppression *in vivo*; Immune activation; |
| Hanyu et al., 2008 | CD40L | Colon/liver | RCN9; | Tumor growth suppression *in vivo*; |
| Vardouli et al., 2009 | CD40L | Ovarian, bladder and cervical | CaSki, HeLa, EJ, T24, VM-CUB1, AGE60; | Proliferation inhibition *in vitro* in CD40 positive cell lines; tumor growth inhibition *in vivo* in bladder carcinoma murine model; |
| Mukogawa et al., 2003 | iκB α truncated + radiotherapy | Colon | HT29, HCT15; | Cell growth inhibition *in vitro* and tumor growth *in vivo*; |
| Sumitomo et al., 1999 | iκB α | Bladder | KU-19-19, KU-1, KU-7, T-24; | Growth inhibition, apoptosis induction, cytokines secretion suppression in KU-19-19; |
| Tosch et al., 2009 | PAMPs | Lung, colon, melanoma and renal | A549, B16F0-CAR, CT26, TC1, RenCa-MUC1; | Tumor growth inhibition *in vivo* in melanoma murine model (B16F0-CAR); Transduction of PAMPs in combination with tumors antigens generated higher tumor regression and acted as a vaccine; |

## References


Barton KN, Siddiqui F, Pompa R, Freytag SO, Khan G, Dobrosotskaya I, Ajlouni M, Zhang Y, Cheng J, Movsas B *et al.* (2021) Phase I trial of oncolytic adenovirus-mediated cytotoxic and interleukin-12 gene therapy for the treatment of metastatic pancreatic cancer. Mol Ther Oncolytics 20:94–104.

Bortolanza S, Bunuales M, Otano I, Gonzalez-Aseguinolaza G, Ortiz-de-Solorzano C, Perez D, Prieto J and Hernandez-Alcoceba R (2009) Treatment of pancreatic cancer with an oncolytic adenovirus expressing interleukin-12 in Syrian hamsters. Mol Ther 17:614–622.

Cao G, Su J, Lu W, Zhang F, Zhao G, Marteralli D and Dong Z (2001) Adenovirus-mediated interferon-beta gene therapy suppresses growth and metastasis of human prostate cancer in nude mice. Cancer Gene Ther 8:497-505.

Cao X, Wei R, Liu X, Zeng Y, Huang H, Ding M, Zhang K and Liu XY (2011a) Cancer targeting Gene-Viro-Therapy specific for liver cancer by α-fetoprotein-controlled oncolytic adenovirus expression of SOCS3 and IL-24. Acta Biochim Biophy Sin (Shangay) 43:813–821.

Cao X, Yang M, Wei RC, Zeng Y, Gu JF, Huang WD, Yang DQ, Li HL, Ding M, Wei N *et al.* (2011b) Cancer targeting Gene-Viro-Therapy of liver carcinoma by dual-regulated oncolytic adenovirus armed with TRAIL gene. Gene Ther 18:765–777.

Chang S, Yang J, Chen W, Xie Y and Sheng W (2011) Antitumor activity of an adenovirus harboring human IL-24 in colon cancer. Mol Biol Rep 38:395–401.

Chaurasiya S, Hew P, Crosley P, Sharon D, Potts K, Agopsowicz K, Long M, Shi C and Hitt MM (2016) Breast cancer gene therapy using an adenovirus encoding human IL-2 under control of mammaglobin promoter/enhancer sequences. Cancer Gene Ther 23:178–187.

Dummer R, Rochlitx C, Velu T, Acres B, Limacher JM, Bleuzen P, Lacoste G, Slos P, Romero P, Urosevic M (2008) Itralesional adenovirus-mediated interleukin-2 gene transfer for advanced solid cancers and melanoma. Mol Ther 16:985-94.

Freytag SO, Barton KN and Zhang Y (2013) Efficacy of oncolytic adenovirus expressing suicide genes and interleukin-12 in preclinical model of prostate cancer. Gene Ther 20:1131–1139.

Furukawa K, Ohashi T, Haruki K, Fujiwara Y, Iida T, Shiba H, Uwagawa T, Kobayashi H and Yanaga K (2011) Combination treatment using adenovirus vector-mediated tumor necrosis factor-alpha gene transfer and a NF-κB inhibitor for pancreatic cancer in mice. Cancer Lett 306:92–98.

Hanyu K, Iida T, Shiba H, Ohashi T, Eto Y and Yanaga K (2008) Immunogene therapy by adenovirus vector expressing CD40 ligand for metastatic liver cancer in rats. Anticancer Res 28:2785–2789.

Hara H, Kobayashi A, Yoshida K, Ohashi M, Ohnami S, Uchida E, Higashihara E, Yoshida T and Aoki K (2007) Local interferon-α gene therapy elicits systemic immunity in a syngeneic pancreatic cancer model in hamster. Cancer Sci 98:455–463.

He LF, Gu JF, Tang WH, Fan JK, Wei N, Zou WG, Zhang YH, Zhao LL and Liu XY (2008) Significant antitumor activity of oncolytic adenovirus expressing human interferon-β for hepatocellular carcinoma. J Gene Med 10:983–992

Iida T, Shiba H, Misawa T, Ohashi T, Eto Y and Yanaga K (2010) Immunogene therapy against colon cancer metastasis using an adenovirus vector expressing CD40 ligand. Surgery 148:925–935.

Jiang J, Zhang Y, Peng K, Wang Q, Hong X, Li H, Fan G, Zhang Z, Gong T and Sun X (2017) Combined delivery of a TGF-β inhibitor and an adenoviral vector expressing interleukin-12 potentiates cancer immunotherapy. Acta Biomater 61:114–123.

Jiang Y-Q, Zhang Z, Cai H-R and Zhou H (2015b) Killing effect of TNF-mediated by conditionally replicating adenovirus on esophageal cancer and lung cancer cell lines. 8:13785-13794

Larocca CJ, Han J, Gavrikova T, Armstrong L, Oliveira AR, Shanley R, Vickers SM, Yamamoto M and Davydova J (2015) Oncolytic adenovirus expressing interferon alpha in a syngeneic Syrian hamster model for the treatment of pancreatic cancer. Surgery (United States) 157:888–898.

Li L, Li F, Tian H, Yue W, Li S and Chen G (2014) Human mesenchymal stem cells with adenovirus-mediated TRAIL gene transduction have antitumor effects on esophageal cancer cell line Eca-109. Acta Biochim Biophys Sin 46:471–476.

Liu J, Zhang Y, Sun P, Xie Y, Xiang J and Yang J (2013a) Enhanced therapeutic efficacy of adenovirus-mediated interleukin-24 gene therapy combined with ionizing radiotherapy for nasopharyngeal carcinoma. Oncol Rep 30:1165–1174.

Mao L, Yang C, Li L, Nai L, Fan L, Wang J, Li W, Wen R, Chen J and Zheng J (2014) Replication-competent adenovirus expressing TRAIL synergistically potentiates the antitumor effect of gemcitabine in bladder cancer cells. Tumor Biol 35:5937–5944

Mukogawa T, Koyama F, Tachibana M, Takayanagi A, Shimizu N, Fujii H, Ueno M, Matsumoto H, Takeuchi T and Nakajima Y (2003) Adenovirus-mediated gene transduction of truncated IκBα α α α enhances radiosensitivity in human colon cancer cells. Cancer Sci 94:745-750.

Oh YT, Chen DWC, Dougherty GJ and McBride WH (2004) Adenoviral interleukin-3 gene-radiation therapy for prostate cancer in mouse model. Int J Radiat Oncol Biol Phys 59:579–583.

Ohashi M, Yoshida K, Kushida M, Miura Y, Ohnami S, Ikarashi Y, Kitade Y, Yoshida T and Aoki K (2005) Adenovirus-mediated interferon α gene transfer induces regional direct cytotoxicity and possible systemic immunity against pancreatic cancer. Br J Cancer 93:441–449.

Park MY, Kim DR, Jung HW, Yoon HI, Lee JH and Lee CT (2010) Genetic immunotherapy of lung cancer using conditionally replicating adenovirus and adenovirus-interferon-β. Cancer Gene Ther 17:356–364.

Sangro B, Mazzolini G, Ruiz J, Herraiz M, Quiroga J, Herrero I, Benito A, Larrache J, Pueyo J, Subtil JC, Olagüe C, Sola J, Sádaba B, Lacasa C, Melero I, Qian C, Prieto J (2004) Phase I trial of intratumoral injection of an adenovirus encoding interleukin-12 for advanced digestive tumors. J Clin Oncol 22:1389-1397.

Shim SH, Lee CT, Hun Hah J, Lee JJ, Park SW, Heo DS and Sung MW (2010) Conditionally replicating adenovirus improves gene replication efficiency and anticancer effect of E1-deleted adenovirus carrying TRAIL in head and neck squamous cell carcinoma. Cancer Sci 101:482–487.

Stewart AK, Lassam NJ, Quirt IC, Bailey DJ, Rotstein LE, Krajden M, Dessureault D, Galinger S, Cappe D, Wan Y, Addison CL, Moen RC, Gauldie J, Graham FL (1999) Adenovector-mediated gene delivery of interleukin-2 in metastatic breast cancer and melanoma: results of a phase 1 clinical trial. Gene Ther 6:350-63.

Sumitomo M, Tachibana M, Ozu C, Asakura H, Murai M, Hayakawa M, Nakamura H, Takayanagi A and Shimizu N (1999) Induction of Apoptosis of Cytokine-Producing Bladder Cancer Cells by Adenovirus-Mediated I k B a Overexpression. Hum Gene Ther 10:37-47

Tong AW, Nemunaitis J, Su D, Zhang Y, Cunningham C, Senzer N, Netto G, Rich D, Mhashilkar A, Parker K *et al.* (2005) Intratumoral injection of INGN241, a nonreplicative adenovector expressing the melanoma-differentiation associated gene-7 (mda-7/IL24) biologic outcome in advanced cancer patients. Mol Ther 11:160-72.

Tosch C, Geist M, Ledoux C, Ziller-Remi C, Paul S, Erbs P, Corvaia N, Von Hoegen P, Balloul JM and Haegel H (2009) Adenovirus-mediated gene transfer of pathogen-associated molecular patterns for cancer immunotherapy. Cancer Gene Ther 16:310–319.

Trudel S, Trachtenberg J, Toi A, Sweet L, Li ZH, Jewett M, Tchilias J, Zhuang LH, Hitt M, Wan Y, Gauldie J, Ghaham FL, Dancey J, Stewart AK (2003) A phase I trial of adenovector-mediated delivery of interleukin-2 (AdIL-2) in high-risk localized prostate câncer. Cancer Gene Ther 10:755-63.

Vardouli L, Lindqvist C, Vlahou K, Loskog ASI and Eliopoulos AG (2009) Adenovirus delivery of human CD40 ligand gene confers direct therapeutic effects on carcinomas. Cancer Gene Ther 16:848–860.

Xie FJ, Zhao P, Zhang YP, Liu FY, Nie XL, Zhu YH, Yu XM, Zheng QQ, Mao WM, Lu HY *et al.* (2013) Adenovirus-mediated interferon-γ gene therapy induced human pancreatic carcinoma Capan-2 cell apoptosis in vitro and in vivo. Anat Rec (Hoboken) 296:604–610

Xu Y, Zhang F, Qin L, Miao J, Sheng W, Xie Y, Xu X, Yang J and Qian H (2014) Enhanced in-vitro and in-vivo suppression of A375 melanoma by combined IL-24/OSM adenoviral-mediated gene therapy. Melanoma Res 24:20–27.

Yan Y, Xu H, Wang J, Wu X, Wen W, Liang Y, Wang L, Liu F and Du X (2019a) Inhibition of breast cancer cells by targeting E2F-1 gene and expressing IL15 oncolytic adenovirus. Biosci Rep 39:BSR20190384.

Yang Y, Xu H, Huang W, Ding M, Xiao J, Yang D, Li H, Liu XY and Chu L (2015) Targeting lung cancer stem-like cells with TRAIL gene armed oncolytic adenovirus. J Cell Mol Med 19:915–923.

Yang Z, Zhang Q, Xu K, Shan J, Shen J, Liu L, Xu Y, Xia F, Bie P, Zhang X *et al.* (2012) Combined Therapy with Cytokine-Induced Killer Cells and Oncolytic Adenovirus Expressing IL-12 Induce Enhanced Antitumor Activity in Liver Tumor Model. PloS One 7:e44802.

Zhao P, Zhu YH, Wu JX, Liu RY, Zhu XY, Xiao X, Li HL, Huang BJ, Xie FJ, Chen JM *et al.* (2007) Adenovirus-mediated delivery of human IFNγ gene inhibits prostate cancer growth. Life Sci 1:695–701.

Zhao Y, Li Z, Sheng W, Miao J and Yang J (2013) Radiosensitivity by ING4-IL-24 bicistronic adenovirus-mediated gene cotransfer on human breast cancer cells. Cancer Gene Ther 20:38–45.

Zhou W, Dai S, Zhu H, Song Z, Cai Y, Lee JB, Li Z, Hu X, Fang B, He C *et al.* (2017) Telomerase-specific oncolytic adenovirus expressing TRAIL suppresses peritoneal dissemination of gastric cancer. Gene Ther 24:199–207.
